# Supplementary material for: Resolving the full spectrum of human genome variation using Linked-Reads
Source: Genome Res. 2019 Apr;29(4):635–45. doi: 10.1101/gr.234443.118 (PMC6442396; doi:10.1101/gr.234443.118)
Supplement: Supplemental Material [file supp_gr.234443.118_Supplemental_longranger-2.2.zip › longranger-2.2.tar.bz2!/lr2.2-public/lib/stan/cmdstan/stan/doc/api/html/generator_8hpp_source.html]

Stan: src/stan/gm/generator.hpp Source File


|  |  |
| --- | --- |
|  | Stan  2.5.0  probability, sampling & optimization |


- Main Page
- Related Pages
- Namespaces
- Classes
- Files

- File List
- File Members

All Classes Namespaces Files Functions Variables Typedefs Enumerations Enumerator Friends Macros Pages

- src
- stan
- gm

generator.hpp

Go to the documentation of this file.

1 #ifndef STAN\_\_GM\_\_GENERATOR\_HPP

2 #define STAN\_\_GM\_\_GENERATOR\_HPP

3

4 #include <boost/variant/apply\_visitor.hpp>

5 #include <boost/lexical\_cast.hpp>

6

7 #include <cstddef>

8 #include <iostream>

9 #include <ostream>

10 #include <sstream>

11 #include <stdexcept>

12 #include <string>

13 #include <vector>

14

15 #include <stan/version.hpp>

16 #include <stan/gm/ast.hpp>

17

18 namespace stan {

19

20  namespace gm {

21

22  void generate\_expression(const expression& e, std::ostream& o);

23

24  const std::string EOL("\n");

25  const std::string EOL2("\n\n");

26  const std::string INDENT(" ");

27  const std::string INDENT2(" ");

28  const std::string INDENT3(" ");

29

30  template <typename D>

31  bool has\_lub(const D& x) {

32  return !is\_nil(x.range\_.low\_.expr\_) && !is\_nil(x.range\_.high\_.expr\_);

33  }

34  template <typename D>

35  bool has\_ub(const D& x) {

36  return is\_nil(x.range\_.low\_.expr\_) && !is\_nil(x.range\_.high\_.expr\_);

37  }

38  template <typename D>

39  bool has\_lb(const D& x) {

40  return !is\_nil(x.range\_.low\_.expr\_) && is\_nil(x.range\_.high\_.expr\_);

41  }

42

43  template <typename T>

44  std::string to\_string(T i) {

45  std::stringstream ss;

46  ss << i;

47  return ss.str();

48  }

49

50  void generate\_indent(size\_t indent, std::ostream& o) {

51  for (size\_t k = 0; k < indent; ++k)

52  o << INDENT;

53  }

54

55  void generate\_void\_statement(const std::string& name,

56  const size\_t indent,

57  std::ostream& o) {

58  generate\_indent(indent, o);

59  o << "(void) " << name << "; // dummy to suppress unused var warning";

60  o << EOL;

61  }

62

64  struct visgen {

65  typedef void result\_type;

66  std::ostream& o\_;

67  visgen(std::ostream& o) : o\_(o) { }

68  };

69

70  void generate\_start\_namespace(std::string name,

71  std::ostream& o) {

72  o << "namespace " << name << "\_namespace {" << EOL2;

73  }

74

75  void generate\_end\_namespace(std::ostream& o) {

76  o << "} // namespace" << EOL2;

77  }

78

79  void generate\_comment(std::string const& msg, int indent,

80  std::ostream& o) {

81  generate\_indent(indent,o);

82  o << "// " << msg << EOL;

83  }

84

85

86  template <bool isLHS>

87  void generate\_indexed\_expr(const std::string& expr,

88  const std::vector<expression> indexes,

89  base\_expr\_type base\_type, // may have more dims

90  size\_t e\_num\_dims, // array dims

91  std::ostream& o) {

92  // FIXME: add more get\_base1 functions and fold nested calls into API

93  // up to a given size, then default to this behavior

94  size\_t ai\_size = indexes.size();

95  if (ai\_size == 0) {

96  // no indexes

97  o << expr;

98  return;

99  }

100  if (ai\_size <= (e\_num\_dims + 1) || base\_type != MATRIX\_T) {

101  for (size\_t n = 0; n < ai\_size; ++n)

102  o << (isLHS ? "get\_base1\_lhs(" : "get\_base1(");

103  o << expr;

104  for (size\_t n = 0; n < ai\_size; ++n) {

105  o << ',';

106  generate\_expression(indexes[n],o);

107  o << ',' << '"' << expr << '"' << ',' << (n+1) << ')';

108  }

109  } else {

110  for (size\_t n = 0; n < ai\_size - 1; ++n)

111  o << (isLHS ? "get\_base1\_lhs(" : "get\_base1(");

112  o << expr;

113  for (size\_t n = 0; n < ai\_size - 2; ++n) {

114  o << ',';

115  generate\_expression(indexes[n],o);

116  o << ',' << '"' << expr << '"' << ',' << (n+1) << ')';

117  }

118  o << ',';

119  generate\_expression(indexes[ai\_size - 2U],o);

120  o << ',';

121  generate\_expression(indexes[ai\_size - 1U],o);

122  o << ',' << '"' << expr << '"' << ',' << (ai\_size-1U) << ')';

123  }

124  }

125

126  void generate\_type(const std::string& base\_type,

127  const std::vector<expression>& /\*dims\*/,

128  size\_t end,

129  std::ostream& o) {

130  for (size\_t i = 0; i < end; ++i) o << "std::vector<";

131  o << base\_type;

132  for (size\_t i = 0; i < end; ++i) {

133  if (i > 0) o << ' ';

134  o << '>';

135  }

136  }

137

138  struct expression\_visgen : public visgen {

139  expression\_visgen(std::ostream& o) : visgen(o) { }

140  void operator()(nil const& /\*x\*/) const {

141  o\_ << "nil";

142  }

143  void operator()(const int\_literal& n) const { o\_ << n.val\_; }

144  void operator()(const double\_literal& x) const {

145  std::string num\_str = boost::lexical\_cast<std::string>(x.val\_);

146  o\_ << num\_str;

147  if (num\_str.find\_first\_of("eE.") == std::string::npos)

148  o\_ << ".0"; // trailing 0 to ensure C++ makes it a double

149  }

150  void operator()(const array\_literal& x) const {

151  o\_ << "stan::math::new\_array<";

152  generate\_type("foobar",

153  x.args\_,

154  x.args\_.size(),

155  o\_);

156  o\_ << ">()";

157  for (size\_t i = 0; i < x.args\_.size(); ++i) {

158  o\_ << ".add(";

159  generate\_expression(x.args\_[i],o\_);

160  o\_ << ")";

161  }

162  o\_ << ".array()";

163  }

164  void operator()(const variable& v) const { o\_ << v.name\_; }

165  void operator()(int n) const { o\_ << static\_cast<long>(n); }

166  void operator()(double x) const { o\_ << x; }

167  void operator()(const std::string& x) const { o\_ << x; } // identifiers

168  void operator()(const index\_op& x) const {

169  std::stringstream expr\_o;

170  generate\_expression(x.expr\_,expr\_o);

171  std::string expr\_string = expr\_o.str();

172  std::vector<expression> indexes;

173  size\_t e\_num\_dims = x.expr\_.expression\_type().num\_dims\_;

174  base\_expr\_type base\_type = x.expr\_.expression\_type().base\_type\_;

175  for (size\_t i = 0; i < x.dimss\_.size(); ++i)

176  for (size\_t j = 0; j < x.dimss\_[i].size(); ++j)

177  indexes.push\_back(x.dimss\_[i][j]); // wasteful copy, could use refs

178  generate\_indexed\_expr<false>(expr\_string,indexes,base\_type,e\_num\_dims,o\_);

179  }

180  void operator()(const integrate\_ode& fx) const {

181  o\_ << "integrate\_ode("

182  << fx.system\_function\_name\_

183  << "\_functor\_\_(), ";

184

185  generate\_expression(fx.y0\_, o\_);

186  o\_ << ", ";

187

188  generate\_expression(fx.t0\_, o\_);

189  o\_ << ", ";

190

191  generate\_expression(fx.ts\_, o\_);

192  o\_ << ", ";

193

194  generate\_expression(fx.theta\_, o\_);

195  o\_ << ", ";

196

197  generate\_expression(fx.x\_, o\_);

198  o\_ << ", ";

199

200  generate\_expression(fx.x\_int\_, o\_);

201  o\_ << ", pstream\_\_)";

202  }

203  void operator()(const fun& fx) const {

204  // first test if short-circuit op (binary && and || applied to

205  // primitives; overloads are eager, not short-circuiting)

206  if (fx.name\_ == "logical\_or" || fx.name\_ == "logical\_and") {

207  o\_ << "(primitive\_value(";

208  boost::apply\_visitor(\*this, fx.args\_[0].expr\_);

209  o\_ << ") " << ((fx.name\_ == "logical\_or") ? "||" : "&&") << " primitive\_value(";

210  boost::apply\_visitor(\*this, fx.args\_[1].expr\_);

211  o\_ << "))";

212  return;

213  }

214  o\_ << fx.name\_ << '(';

215  for (size\_t i = 0; i < fx.args\_.size(); ++i) {

216  if (i > 0) o\_ << ',';

217  boost::apply\_visitor(\*this, fx.args\_[i].expr\_);

218  }

219  if (fx.args\_.size() > 0

220  && (has\_rng\_suffix(fx.name\_) || has\_lp\_suffix(fx.name\_)))

221  o\_ << ", ";

222  if (has\_rng\_suffix(fx.name\_))

223  o\_ << "base\_rng\_\_";

224  if (has\_lp\_suffix(fx.name\_))

225  o\_ << "lp\_\_, lp\_accum\_\_";

226  if (is\_user\_defined(fx)) {

227  if (fx.args\_.size() > 0

228  || has\_rng\_suffix(fx.name\_)

229  || has\_lp\_suffix(fx.name\_))

230  o\_ << ", ";

231  o\_ << "pstream\_\_";

232  }

233  o\_ << ')';

234  }

235  void operator()(const binary\_op& expr) const {

236  o\_ << '(';

237  boost::apply\_visitor(\*this, expr.left.expr\_);

238  o\_ << ' ' << expr.op << ' ';

239  boost::apply\_visitor(\*this, expr.right.expr\_);

240  o\_ << ')';

241  }

242  void operator()(const unary\_op& expr) const {

243  o\_ << expr.op << '(';

244  boost::apply\_visitor(\*this, expr.subject.expr\_);

245  o\_ << ')';

246  }

247  };

248

249  void generate\_expression(const expression& e, std::ostream& o) {

250  expression\_visgen vis(o);

251  boost::apply\_visitor(vis, e.expr\_);

252  }

253

254  static void print\_string\_literal(std::ostream& o,

255  const std::string& s) {

256  o << '"';

257  for (size\_t i = 0; i < s.size(); ++i) {

258  if (s[i] == '"' || s[i] == '\\' || s[i] == '\'' )

259  o << '\\'; // escape

260  o << s[i];

261  }

262  o << '"';

263  }

264

265  static void print\_quoted\_expression(std::ostream& o,

266  const expression& e) {

267  std::stringstream ss;

268  generate\_expression(e,ss);

269  print\_string\_literal(o,ss.str());

270  }

271

272  struct printable\_visgen : public visgen {

273  printable\_visgen(std::ostream& o) : visgen(o) { }

274  void operator()(const std::string& s) const {

275  print\_string\_literal(o\_,s);

276  }

277  void operator()(const expression& e) const {

278  generate\_expression(e,o\_);

279  // print\_quoted\_expression(o\_,e);

280  }

281  };

282

283  void generate\_printable(const printable& p, std::ostream& o) {

284  printable\_visgen vis(o);

285  boost::apply\_visitor(vis, p.printable\_);

286  }

287

288  void generate\_using(const std::string& type, std::ostream& o) {

289  o << "using " << type << ";" << EOL;

290  }

291

292  void generate\_using\_namespace(const std::string& ns, std::ostream& o) {

293  o << "using namespace " << ns << ";" << EOL;

294  }

295

296

297  void generate\_usings(std::ostream& o) {

298  generate\_using("std::istream",o);

299  generate\_using("std::string",o);

300  generate\_using("std::stringstream",o);

301  generate\_using("std::vector",o);

302  generate\_using("stan::io::dump",o);

303  generate\_using("stan::math::lgamma",o);

304  generate\_using("stan::model::prob\_grad",o);

305  generate\_using\_namespace("stan::math",o);

306  generate\_using\_namespace("stan::prob",o);

307  o << EOL;

308  }

309

310  void generate\_typedef(const std::string& type,

311  const std::string& abbrev,

312  std::ostream& o) {

313  o << "typedef" << " " << type << " " << abbrev << ";" << EOL;

314  }

315

316  void generate\_typedefs(std::ostream& o) {

317  generate\_typedef("Eigen::Matrix<double,Eigen::Dynamic,1>","vector\_d",o);

318  generate\_typedef("Eigen::Matrix<double,1,Eigen::Dynamic>","row\_vector\_d",o);

319  generate\_typedef("Eigen::Matrix<double,Eigen::Dynamic,Eigen::Dynamic>","matrix\_d",o);

320  o << EOL;

321  }

322

323  void generate\_include(const std::string& lib\_name, std::ostream& o) {

324  o << "#include" << " " << "<" << lib\_name << ">" << EOL;

325  }

326

327  void generate\_includes(std::ostream& o) {

328  generate\_include("stan/model/model\_header.hpp",o);

329  generate\_include("stan/common/command.hpp",o);

330  // generate\_include("boost/random/linear\_congruential.hpp",o);

331  o << EOL;

332  }

333

334  void generate\_version\_comment(std::ostream& o) {

335  o << "// Code generated by Stan version "

336  << stan::MAJOR\_VERSION << "." << stan::MINOR\_VERSION << EOL2;

337  }

338

339  void generate\_class\_decl(const std::string& model\_name,

340  std::ostream& o) {

341  o << "class " << model\_name << " : public prob\_grad {" << EOL;

342  }

343

344  void generate\_end\_class\_decl(std::ostream& o) {

345  o << "}; // model" << EOL2;

346  }

347

348  void generate\_initializer(std::ostream& o,

349  const std::string& base\_type,

350  const std::vector<expression>& dims,

351  const expression& type\_arg1 = expression(),

352  const expression& type\_arg2 = expression()) {

353  for (size\_t i = 0; i < dims.size(); ++i) {

354  o << '(';

355  generate\_expression(dims[i].expr\_,o);

356  o << ',';

357  generate\_type(base\_type,dims,dims.size()- i - 1,o);

358  }

359

360  o << '(';

361  if (!is\_nil(type\_arg1)) {

362  generate\_expression(type\_arg1.expr\_,o);

363  if (!is\_nil(type\_arg2)) {

364  o << ',';

365  generate\_expression(type\_arg2.expr\_,o);

366  }

367  } else if (!is\_nil(type\_arg2.expr\_)) {

368  generate\_expression(type\_arg2.expr\_,o);

369  } else {

370  o << '0';

371  }

372  o << ')';

373

374  for (size\_t i = 0; i < dims.size(); ++i)

375  o << ')';

376  o << ';' << EOL;

377  }

378

379  // only generates the test

380  void generate\_validate\_context\_size(std::ostream& o,

381  const std::string& stage,

382  const std::string& var\_name,

383  const std::string& base\_type,

384  const std::vector<expression>& dims,

385  const expression& type\_arg1 = expression(),

386  const expression& type\_arg2 = expression()) {

387  o << INDENT2

388  << "context\_\_.validate\_dims("

389  << '"' << stage << '"'

390  << ", " << '"' << var\_name << '"'

391  << ", " << '"' << base\_type << '"'

392  << ", context\_\_.to\_vec(";

393  for (size\_t i = 0; i < dims.size(); ++i) {

394  if (i > 0) o << ",";

395  generate\_expression(dims[i].expr\_,o);

396  }

397  if (!is\_nil(type\_arg1)) {

398  if (dims.size() > 0) o << ",";

399  generate\_expression(type\_arg1.expr\_,o);

400  if (!is\_nil(type\_arg2)) {

401  o << ",";

402  generate\_expression(type\_arg2.expr\_,o);

403  }

404  }

405  o << "));"

406  << EOL;

407  }

408

409  struct var\_size\_validating\_visgen : public visgen {

410  const std::string stage\_;

411  var\_size\_validating\_visgen(std::ostream& o, const std::string& stage)

412  : visgen(o),

413  stage\_(stage) {

414  }

415  void operator()(nil const& /\*x\*/) const { } // dummy

416  void operator()(int\_var\_decl const& x) const {

417  generate\_validate\_context\_size(o\_,stage\_,x.name\_,"int",x.dims\_);

418  }

419  void operator()(double\_var\_decl const& x) const {

420  generate\_validate\_context\_size(o\_,stage\_,x.name\_,"double",x.dims\_);

421  }

422  void operator()(vector\_var\_decl const& x) const {

423  generate\_validate\_context\_size(o\_,stage\_,x.name\_,"vector\_d",x.dims\_,x.M\_);

424  }

425  void operator()(row\_vector\_var\_decl const& x) const {

426  generate\_validate\_context\_size(o\_,stage\_,x.name\_,"row\_vector\_d",x.dims\_,x.N\_);

427  }

428  void operator()(unit\_vector\_var\_decl const& x) const {

429  generate\_validate\_context\_size(o\_,stage\_,x.name\_,"vector\_d",x.dims\_,x.K\_);

430  }

431  void operator()(simplex\_var\_decl const& x) const {

432  generate\_validate\_context\_size(o\_,stage\_,x.name\_,"vector\_d",x.dims\_,x.K\_);

433  }

434  void operator()(ordered\_var\_decl const& x) const {

435  generate\_validate\_context\_size(o\_,stage\_,x.name\_,"vector\_d",x.dims\_,x.K\_);

436  }

437  void operator()(positive\_ordered\_var\_decl const& x) const {

438  generate\_validate\_context\_size(o\_,stage\_,x.name\_,"vector\_d",x.dims\_,x.K\_);

439  }

440  void operator()(matrix\_var\_decl const& x) const {

441  generate\_validate\_context\_size(o\_,stage\_,x.name\_,"matrix\_d",x.dims\_,x.M\_,x.N\_);

442  }

443  void operator()(cholesky\_factor\_var\_decl const& x) const {

444  generate\_validate\_context\_size(o\_,stage\_,x.name\_,"matrix\_d",x.dims\_,x.M\_,x.N\_);

445  }

446  void operator()(cholesky\_corr\_var\_decl const& x) const {

447  generate\_validate\_context\_size(o\_,stage\_,x.name\_,"matrix\_d",x.dims\_,x.K\_,x.K\_);

448  }

449  void operator()(cov\_matrix\_var\_decl const& x) const {

450  generate\_validate\_context\_size(o\_,stage\_,x.name\_,"matrix\_d",x.dims\_,x.K\_,x.K\_);

451  }

452  void operator()(corr\_matrix\_var\_decl const& x) const {

453  generate\_validate\_context\_size(o\_,stage\_,x.name\_,"matrix\_d",x.dims\_,x.K\_,x.K\_);

454  }

455  };

456

457

458  void generate\_validate\_positive(const std::string& var\_name,

459  const expression& expr,

460  std::ostream& o) {

461  o << INDENT2;

462  o << "stan::math::validate\_non\_negative\_index(\"" << var\_name << "\", ";

463  print\_quoted\_expression(o,expr);

464  o << ", ";

465  generate\_expression(expr,o);

466  o << ");" << EOL;

467  }

468

469  void generate\_initialization(std::ostream& o,

470  const std::string& var\_name,

471  const std::string& base\_type,

472  const std::vector<expression>& dims,

473  const expression& type\_arg1 = expression(),

474  const expression& type\_arg2 = expression()) {

475  // validate all dims are positive

476  for (size\_t i = 0; i < dims.size(); ++i)

477  generate\_validate\_positive(var\_name,dims[i],o);

478  if (!is\_nil(type\_arg1))

479  generate\_validate\_positive(var\_name,type\_arg1,o);

480  if (!is\_nil(type\_arg2))

481  generate\_validate\_positive(var\_name,type\_arg2,o);

482

483  // define variable with initializer

484  o << INDENT2

485  << var\_name << " = ";

486  generate\_type(base\_type,dims,dims.size(),o);

487  generate\_initializer(o,base\_type,dims,type\_arg1,type\_arg2);

488

489  }

490

491  struct var\_resizing\_visgen : public visgen {

492  var\_resizing\_visgen(std::ostream& o)

493  : visgen(o) {

494  }

495  void operator()(nil const& /\*x\*/) const { } // dummy

496  void operator()(int\_var\_decl const& x) const {

497  generate\_initialization(o\_,x.name\_,"int",x.dims\_);

498  }

499  void operator()(double\_var\_decl const& x) const {

500  generate\_initialization(o\_,x.name\_,"double",x.dims\_);

501  }

502  void operator()(vector\_var\_decl const& x) const {

503  generate\_initialization(o\_,x.name\_,"vector\_d",x.dims\_,x.M\_);

504  }

505  void operator()(row\_vector\_var\_decl const& x) const {

506  generate\_initialization(o\_,x.name\_,"row\_vector\_d",x.dims\_,x.N\_);

507  }

508  void operator()(unit\_vector\_var\_decl const& x) const {

509  generate\_initialization(o\_,x.name\_,"vector\_d",x.dims\_,x.K\_);

510  }

511  void operator()(simplex\_var\_decl const& x) const {

512  generate\_initialization(o\_,x.name\_,"vector\_d",x.dims\_,x.K\_);

513  }

514  void operator()(ordered\_var\_decl const& x) const {

515  generate\_initialization(o\_,x.name\_,"vector\_d",x.dims\_,x.K\_);

516  }

517  void operator()(positive\_ordered\_var\_decl const& x) const {

518  generate\_initialization(o\_,x.name\_,"vector\_d",x.dims\_,x.K\_);

519  }

520  void operator()(matrix\_var\_decl const& x) const {

521  generate\_initialization(o\_,x.name\_,"matrix\_d",x.dims\_,x.M\_,x.N\_);

522  }

523  void operator()(cholesky\_factor\_var\_decl const& x) const {

524  generate\_initialization(o\_,x.name\_,"matrix\_d",x.dims\_,x.M\_,x.N\_);

525  }

526  void operator()(cholesky\_corr\_var\_decl const& x) const {

527  generate\_initialization(o\_,x.name\_,"matrix\_d",x.dims\_,x.K\_,x.K\_);

528  }

529  void operator()(cov\_matrix\_var\_decl const& x) const {

530  generate\_initialization(o\_,x.name\_,"matrix\_d",x.dims\_,x.K\_,x.K\_);

531  }

532  void operator()(corr\_matrix\_var\_decl const& x) const {

533  generate\_initialization(o\_,x.name\_,"matrix\_d",x.dims\_,x.K\_,x.K\_);

534  }

535  };

536

537  void generate\_var\_resizing(const std::vector<var\_decl>& vs,

538  std::ostream& o) {

539  var\_resizing\_visgen vis(o);

540  for (size\_t i = 0; i < vs.size(); ++i)

541  boost::apply\_visitor(vis, vs[i].decl\_);

542  }

543

544  const std::vector<expression> EMPTY\_EXP\_VECTOR(0);

545

546  struct init\_local\_var\_visgen : public visgen {

547  const bool declare\_vars\_;

548  const bool is\_var\_;

549  init\_local\_var\_visgen(bool declare\_vars,

550  bool is\_var,

551  std::ostream& o)

552  : visgen(o),

553  declare\_vars\_(declare\_vars),

554  is\_var\_(is\_var) {

555  }

556  template <typename D>

557  void generate\_initialize\_array\_bounded(const D& x, const std::string& base\_type,

558  const std::string& read\_fun\_prefix,

559  const std::vector<expression>& dim\_args) const {

560  std::vector<expression> read\_args;

561  std::string read\_fun(read\_fun\_prefix);

562  if (has\_lub(x)) {

563  read\_fun += "\_lub";

564  read\_args.push\_back(x.range\_.low\_);

565  read\_args.push\_back(x.range\_.high\_);

566  } else if (has\_lb(x)) {

567  read\_fun += "\_lb";

568  read\_args.push\_back(x.range\_.low\_);

569  } else if (has\_ub(x)) {

570  read\_fun += "\_ub";

571  read\_args.push\_back(x.range\_.high\_);

572  }

573  for (size\_t i = 0; i < dim\_args.size(); ++i)

574  read\_args.push\_back(dim\_args[i]);

575  generate\_initialize\_array(base\_type,read\_fun,read\_args,x.name\_,x.dims\_);

576  }

577  void operator()(const nil& /\*x\*/) const { }

578  void operator()(const int\_var\_decl& x) const {

579  generate\_initialize\_array("int","integer",EMPTY\_EXP\_VECTOR,x.name\_,x.dims\_);

580  }

581  void operator()(const double\_var\_decl& x) const {

582  std::vector<expression> read\_args;

583  generate\_initialize\_array\_bounded(x,is\_var\_?"T\_\_":"double","scalar",read\_args);

584  }

585  void operator()(const vector\_var\_decl& x) const {

586  std::vector<expression> read\_args;

587  read\_args.push\_back(x.M\_);

588  generate\_initialize\_array\_bounded(x,is\_var\_?"Eigen::Matrix<T\_\_,Eigen::Dynamic,1> ":"vector\_d","vector",read\_args);

589  }

590  void operator()(const row\_vector\_var\_decl& x) const {

591  std::vector<expression> read\_args;

592  read\_args.push\_back(x.N\_);

593  generate\_initialize\_array\_bounded(x,is\_var\_?"Eigen::Matrix<T\_\_,1,Eigen::Dynamic> ":"row\_vector\_d","row\_vector",read\_args);

594  }

595  void operator()(const matrix\_var\_decl& x) const {

596  std::vector<expression> read\_args;

597  read\_args.push\_back(x.M\_);

598  read\_args.push\_back(x.N\_);

599  generate\_initialize\_array\_bounded(x,is\_var\_?"Eigen::Matrix<T\_\_,Eigen::Dynamic,Eigen::Dynamic> ":"matrix\_d","matrix",read\_args);

600  }

601  void operator()(const unit\_vector\_var\_decl& x) const {

602  std::vector<expression> read\_args;

603  read\_args.push\_back(x.K\_);

604  generate\_initialize\_array(is\_var\_?"Eigen::Matrix<T\_\_,Eigen::Dynamic,1> ":"vector\_d","unit\_vector",read\_args,x.name\_,x.dims\_);

605  }

606  void operator()(const simplex\_var\_decl& x) const {

607  std::vector<expression> read\_args;

608  read\_args.push\_back(x.K\_);

609  generate\_initialize\_array(is\_var\_?"Eigen::Matrix<T\_\_,Eigen::Dynamic,1> ":"vector\_d","simplex",read\_args,x.name\_,x.dims\_);

610  }

611  void operator()(const ordered\_var\_decl& x) const {

612  std::vector<expression> read\_args;

613  read\_args.push\_back(x.K\_);

614  generate\_initialize\_array(is\_var\_?"Eigen::Matrix<T\_\_,Eigen::Dynamic,1> ":"vector\_d","ordered",read\_args,x.name\_,x.dims\_);

615  }

616  void operator()(const positive\_ordered\_var\_decl& x) const {

617  std::vector<expression> read\_args;

618  read\_args.push\_back(x.K\_);

619  generate\_initialize\_array(is\_var\_?"Eigen::Matrix<T\_\_,Eigen::Dynamic,1> ":"vector\_d","positive\_ordered",read\_args,x.name\_,x.dims\_);

620  }

621  void operator()(const cholesky\_factor\_var\_decl& x) const {

622  std::vector<expression> read\_args;

623  read\_args.push\_back(x.M\_);

624  read\_args.push\_back(x.N\_);

625  generate\_initialize\_array(is\_var\_?"Eigen::Matrix<T\_\_,Eigen::Dynamic,Eigen::Dynamic> ":"matrix\_d",

626  "cholesky\_factor",read\_args,x.name\_,x.dims\_);

627  }

628  void operator()(const cholesky\_corr\_var\_decl& x) const {

629  std::vector<expression> read\_args;

630  read\_args.push\_back(x.K\_);

631  generate\_initialize\_array(is\_var\_

632  ? "Eigen::Matrix<T\_\_,Eigen::Dynamic,Eigen::Dynamic> "

633  : "matrix\_d",

634  "cholesky\_corr",read\_args,x.name\_,x.dims\_);

635  }

636

637  void operator()(const cov\_matrix\_var\_decl& x) const {

638  std::vector<expression> read\_args;

639  read\_args.push\_back(x.K\_);

640  generate\_initialize\_array(is\_var\_?"Eigen::Matrix<T\_\_,Eigen::Dynamic,Eigen::Dynamic> ":"matrix\_d",

641  "cov\_matrix",read\_args,x.name\_,x.dims\_);

642  }

643  void operator()(const corr\_matrix\_var\_decl& x) const {

644  std::vector<expression> read\_args;

645  read\_args.push\_back(x.K\_);

646  generate\_initialize\_array(is\_var\_?"Eigen::Matrix<T\_\_,Eigen::Dynamic,Eigen::Dynamic> ":"matrix\_d",

647  "corr\_matrix",read\_args,x.name\_,x.dims\_);

648  }

649  void generate\_initialize\_array(const std::string& var\_type,

650  const std::string& read\_type,

651  const std::vector<expression>& read\_args,

652  const std::string& name,

653  const std::vector<expression>& dims) const {

654  if (declare\_vars\_) {

655  o\_ << INDENT2;

656  for (size\_t i = 0; i < dims.size(); ++i) o\_ << "vector<";

657  o\_ << var\_type;

658  for (size\_t i = 0; i < dims.size(); ++i) o\_ << "> ";

659  if (dims.size() == 0) o\_ << " ";

660  o\_ << name << ";" << EOL;

661  }

662

663  if (dims.size() == 0) {

664  generate\_void\_statement(name, 2, o\_);

665  o\_ << INDENT2 << "if (jacobian\_\_)" << EOL;

666

667  // w Jacobian

668  generate\_indent(3,o\_);

669  o\_ << name << " = in\_\_." << read\_type << "\_constrain(";

670  for (size\_t j = 0; j < read\_args.size(); ++j) {

671  if (j > 0) o\_ << ",";

672  generate\_expression(read\_args[j],o\_);

673  }

674  if (read\_args.size() > 0)

675  o\_ << ",";

676  o\_ << "lp\_\_";

677  o\_ << ");" << EOL;

678

679  o\_ << INDENT2 << "else" << EOL;

680

681  // w/o Jacobian

682  generate\_indent(3,o\_);

683  o\_ << name << " = in\_\_." << read\_type << "\_constrain(";

684  for (size\_t j = 0; j < read\_args.size(); ++j) {

685  if (j > 0) o\_ << ",";

686  generate\_expression(read\_args[j],o\_);

687  }

688  o\_ << ");" << EOL;

689

690  } else {

691  // dims > 0

692  std::string name\_dims(name);

693  for (size\_t i = 0; i < dims.size(); ++i) {

694  generate\_indent(i + 2, o\_);

695  o\_ << "size\_t dim\_" << name << "\_" << i << "\_\_ = ";

696  generate\_expression(dims[i],o\_);

697  o\_ << ";" << EOL;

698

699  if (i < dims.size() - 1) {

700  generate\_indent(i + 2, o\_);

701  o\_ << name\_dims << ".resize(dim" << "\_" << name << "\_" << i << "\_\_);"

702  << EOL;

703  name\_dims.append("[k\_").append(to\_string(i)).append("\_\_]");

704  }

705

706  generate\_indent(i + 2, o\_);

707  if (i == dims.size() - 1) {

708  o\_ << name\_dims << ".reserve(dim\_" << name << "\_" << i << "\_\_);" << EOL;

709  generate\_indent(i + 2, o\_);

710  }

711

712  o\_ << "for (size\_t k\_" << i << "\_\_ = 0;"

713  << " k\_" << i << "\_\_ < dim\_" << name << "\_" << i << "\_\_;"

714  << " ++k\_" << i << "\_\_) {" << EOL;

715

716  // if on the last loop, push read element into array

717  if (i == dims.size() - 1) {

718  generate\_indent(i + 3, o\_);

719  o\_ << "if (jacobian\_\_)" << EOL;

720

721  // w Jacobian

722

723  generate\_indent(i + 4, o\_);

724  o\_ << name\_dims << ".push\_back(in\_\_." << read\_type << "\_constrain(";

725  for (size\_t j = 0; j < read\_args.size(); ++j) {

726  if (j > 0) o\_ << ",";

727  generate\_expression(read\_args[j],o\_);

728  }

729  if (read\_args.size() > 0)

730  o\_ << ",";

731  o\_ << "lp\_\_";

732  o\_ << "));" << EOL;

733

734  generate\_indent(i + 3, o\_);

735  o\_ << "else" << EOL;

736

737  // w/o Jacobian

738

739  generate\_indent(i + 4, o\_);

740  o\_ << name\_dims << ".push\_back(in\_\_." << read\_type << "\_constrain(";

741  for (size\_t j = 0; j < read\_args.size(); ++j) {

742  if (j > 0) o\_ << ",";

743  generate\_expression(read\_args[j],o\_);

744  }

745  o\_ << "));" << EOL;

746  }

747  }

748

749  for (size\_t i = dims.size(); i > 0; --i) {

750  generate\_indent(i + 1, o\_);

751  o\_ << "}" << EOL;

752  }

753  }

754  o\_ << EOL;

755  }

756  };

757

758  void generate\_local\_var\_inits(std::vector<var\_decl> vs,

759  bool is\_var,

760  bool declare\_vars,

761  std::ostream& o) {

762  o << INDENT2

763  << "stan::io::reader<"

764  << (is\_var ? "T\_\_" : "double")

765  << "> in\_\_(params\_r\_\_,params\_i\_\_);" << EOL2;

766  init\_local\_var\_visgen vis(declare\_vars,is\_var,o);

767  for (size\_t i = 0; i < vs.size(); ++i)

768  boost::apply\_visitor(vis, vs[i].decl\_);

769  }

770

771

772

773

774  void generate\_public\_decl(std::ostream& o) {

775  o << "public:" << EOL;

776  }

777

778  void generate\_private\_decl(std::ostream& o) {

779  o << "private:" << EOL;

780  }

781

782

783  struct validate\_var\_decl\_visgen : public visgen {

784  int indents\_;

785  validate\_var\_decl\_visgen(int indents,

786  std::ostream& o)

787  : visgen(o),

788  indents\_(indents) {

789  }

790  void generate\_begin\_for\_dims(const std::vector<expression>& dims)

791  const {

792

793  for (size\_t i = 0; i < dims.size(); ++i) {

794  generate\_indent(indents\_+i,o\_);

795  o\_ << "for (int k" << i << "\_\_ = 0;"

796  << " k" << i << "\_\_ < ";

797  generate\_expression(dims[i].expr\_,o\_);

798  o\_ << ";";

799  o\_ << " ++k" << i << "\_\_) {" << EOL;

800  }

801  }

802  void generate\_end\_for\_dims(size\_t dims\_size) const {

803  for (size\_t i = 0; i < dims\_size; ++i) {

804  generate\_indent(indents\_ + dims\_size - i - 1, o\_);

805  o\_ << "}" << EOL;

806  }

807  }

808

809  void generate\_loop\_var(const std::string& name,

810  size\_t dims\_size) const {

811  o\_ << name;

812  for (size\_t i = 0; i < dims\_size; ++i)

813  o\_ << "[k" << i << "\_\_]";

814  }

815  void operator()(nil const& /\*x\*/) const { }

816  template <typename T>

817  void basic\_validate(T const& x) const {

818  if (!(x.range\_.has\_low() || x.range\_.has\_high()))

819  return; // unconstrained

820  generate\_begin\_for\_dims(x.dims\_);

821  generate\_indent(indents\_ + x.dims\_.size(),o\_);

822  o\_ << "try { " << EOL;

823  if (x.range\_.has\_low()) {

824  generate\_indent(indents\_ + 1 + x.dims\_.size(),o\_);

825  o\_ << "check\_greater\_or\_equal(function\_\_,";

826  generate\_loop\_var(x.name\_,x.dims\_.size());

827  o\_ << ",";

828  generate\_expression(x.range\_.low\_.expr\_,o\_);

829  o\_ << ",\"";

830  generate\_loop\_var(x.name\_,x.dims\_.size());

831  o\_ << "\", (double \*)0);" << EOL;

832  }

833  if (x.range\_.has\_high()) {

834  generate\_indent(indents\_ + 1 + x.dims\_.size(),o\_);

835  o\_ << "check\_less\_or\_equal(function\_\_,";

836  generate\_loop\_var(x.name\_,x.dims\_.size());

837  o\_ << ",";

838  generate\_expression(x.range\_.high\_.expr\_,o\_);

839  o\_ << ",\"";

840  generate\_loop\_var(x.name\_,x.dims\_.size());

841  o\_ << "\", (double \*)0);" << EOL;

842  }

843  generate\_indent(indents\_ + x.dims\_.size(),o\_);

844  o\_ << "} catch (const std::exception& e) { "

845  << EOL;

846  generate\_indent(indents\_ + x.dims\_.size() + 1, o\_);

847  o\_ << "throw std::domain\_error(std::string(\"Invalid value of " << x.name\_ << ": \") + std::string(e.what()));"

848  << EOL;

849  generate\_indent(indents\_ + x.dims\_.size(), o\_);

850  o\_ << "};" << EOL;

851  generate\_end\_for\_dims(x.dims\_.size());

852  }

853  void operator()(int\_var\_decl const& x) const {

854  basic\_validate(x);

855  }

856  void operator()(double\_var\_decl const& x) const {

857  basic\_validate(x);

858  }

859  void operator()(vector\_var\_decl const& x) const {

860  basic\_validate(x);

861  }

862  void operator()(row\_vector\_var\_decl const& x) const {

863  basic\_validate(x);

864  }

865  void operator()(matrix\_var\_decl const& x) const {

866  basic\_validate(x);

867  }

868  template <typename T>

869  void nonbasic\_validate(const T& x,

870  const std::string& type\_name) const {

871  generate\_begin\_for\_dims(x.dims\_);

872  generate\_indent(indents\_ + x.dims\_.size(),o\_);

873  o\_ << "try { stan::math::check\_" << type\_name << "(function\_\_,";

874  generate\_loop\_var(x.name\_,x.dims\_.size());

875  o\_ << ",\"";

876  generate\_loop\_var(x.name\_,x.dims\_.size());

877  o\_ << "\", (double \*)0); } catch (const std::exception& e) { throw std::domain\_error(std::string(\"Invalid value of " << x.name\_ << ": \") + std::string(e.what())); };" << EOL;

878  generate\_end\_for\_dims(x.dims\_.size());

879  }

880  void operator()(unit\_vector\_var\_decl const& x) const {

881  nonbasic\_validate(x,"unit\_vector");

882  }

883  void operator()(simplex\_var\_decl const& x) const {

884  nonbasic\_validate(x,"simplex");

885  }

886  void operator()(ordered\_var\_decl const& x) const {

887  nonbasic\_validate(x,"ordered");

888  }

889  void operator()(positive\_ordered\_var\_decl const& x) const {

890  nonbasic\_validate(x,"positive\_ordered");

891  }

892  void operator()(cholesky\_factor\_var\_decl const& x) const {

893  nonbasic\_validate(x,"cholesky\_factor");

894  }

895  void operator()(cholesky\_corr\_var\_decl const& x) const {

896  nonbasic\_validate(x,"cholesky\_factor\_corr");

897  }

898  void operator()(cov\_matrix\_var\_decl const& x) const {

899  nonbasic\_validate(x,"cov\_matrix");

900  }

901  void operator()(corr\_matrix\_var\_decl const& x) const {

902  nonbasic\_validate(x,"corr\_matrix");

903  }

904  };

905

906

907  void generate\_validate\_var\_decl(const var\_decl& decl,

908  int indent,

909  std::ostream& o) {

910  validate\_var\_decl\_visgen vis(indent,o);

911  boost::apply\_visitor(vis,decl.decl\_);

912  }

913

914  void generate\_validate\_var\_decls(const std::vector<var\_decl> decls,

915  int indent,

916  std::ostream& o) {

917  for (size\_t i = 0; i < decls.size(); ++i)

918  generate\_validate\_var\_decl(decls[i],indent,o);

919  }

920

921  // see \_var\_decl\_visgen cut & paste

922  struct member\_var\_decl\_visgen : public visgen {

923  int indents\_;

924  member\_var\_decl\_visgen(int indents,

925  std::ostream& o)

926  : visgen(o),

927  indents\_(indents) {

928  }

929  void operator()(nil const& /\*x\*/) const { }

930  void operator()(int\_var\_decl const& x) const {

931  declare\_array("int",x.name\_,x.dims\_.size());

932  }

933  void operator()(double\_var\_decl const& x) const {

934  declare\_array("double",x.name\_,x.dims\_.size());

935  }

936  void operator()(unit\_vector\_var\_decl const& x) const {

937  declare\_array(("vector\_d"), x.name\_, x.dims\_.size());

938  }

939  void operator()(simplex\_var\_decl const& x) const {

940  declare\_array(("vector\_d"), x.name\_, x.dims\_.size());

941  }

942  void operator()(ordered\_var\_decl const& x) const {

943  declare\_array(("vector\_d"), x.name\_, x.dims\_.size());

944  }

945  void operator()(positive\_ordered\_var\_decl const& x) const {

946  declare\_array(("vector\_d"), x.name\_, x.dims\_.size());

947  }

948  void operator()(cholesky\_factor\_var\_decl const& x) const {

949  declare\_array(("matrix\_d"), x.name\_, x.dims\_.size());

950  }

951  void operator()(const cholesky\_corr\_var\_decl& x) const {

952  declare\_array(("matrix\_d"), x.name\_, x.dims\_.size());

953  }

954  void operator()(cov\_matrix\_var\_decl const& x) const {

955  declare\_array(("matrix\_d"), x.name\_, x.dims\_.size());

956  }

957  void operator()(corr\_matrix\_var\_decl const& x) const {

958  declare\_array(("matrix\_d"), x.name\_, x.dims\_.size());

959  }

960  void operator()(vector\_var\_decl const& x) const {

961  declare\_array(("vector\_d"), x.name\_, x.dims\_.size());

962  }

963  void operator()(row\_vector\_var\_decl const& x) const {

964  declare\_array(("row\_vector\_d"), x.name\_, x.dims\_.size());

965  }

966  void operator()(matrix\_var\_decl const& x) const {

967  declare\_array(("matrix\_d"), x.name\_, x.dims\_.size());

968  }

969  void declare\_array(std::string const& type, std::string const& name,

970  size\_t size) const {

971  for (int i = 0; i < indents\_; ++i)

972  o\_ << INDENT;

973  for (size\_t i = 0; i < size; ++i) {

974  o\_ << "vector<";

975  }

976  o\_ << type;

977  if (size > 0) {

978  o\_ << ">";

979  }

980  for (size\_t i = 1; i < size; ++i) {

981  o\_ << " >";

982  }

983  o\_ << " " << name << ";" << EOL;

984  }

985  };

986

987  void generate\_member\_var\_decls(const std::vector<var\_decl>& vs,

988  int indent,

989  std::ostream& o) {

990  member\_var\_decl\_visgen vis(indent,o);

991  for (size\_t i = 0; i < vs.size(); ++i)

992  boost::apply\_visitor(vis,vs[i].decl\_);

993  }

994

995  // see member\_var\_decl\_visgen cut & paste

996  struct local\_var\_decl\_visgen : public visgen {

997  int indents\_;

998  bool is\_var\_;

999  bool is\_fun\_return\_;

1000  local\_var\_decl\_visgen(int indents,

1001  bool is\_var,

1002  bool is\_fun\_return,

1003  std::ostream& o)

1004  : visgen(o),

1005  indents\_(indents),

1006  is\_var\_(is\_var),

1007  is\_fun\_return\_(is\_fun\_return) {

1008  }

1009  void operator()(nil const& /\*x\*/) const { }

1010  void operator()(int\_var\_decl const& x) const {

1011  std::vector<expression> ctor\_args;

1012  declare\_array("int",ctor\_args,x.name\_,x.dims\_);

1013  }

1014  void operator()(double\_var\_decl const& x) const {

1015  std::vector<expression> ctor\_args;

1016  declare\_array(is\_fun\_return\_

1017  ? "fun\_scalar\_t\_\_"

1018  : ( is\_var\_ ? "T\_\_" : "double" ),

1019  ctor\_args,x.name\_,x.dims\_);

1020  }

1021  void operator()(vector\_var\_decl const& x) const {

1022  std::vector<expression> ctor\_args;

1023  ctor\_args.push\_back(x.M\_);

1024  declare\_array(is\_fun\_return\_

1025  ? "Eigen::Matrix<fun\_scalar\_t\_\_,Eigen::Dynamic,1> "

1026  : ( is\_var\_ ? "Eigen::Matrix<T\_\_,Eigen::Dynamic,1> " : "vector\_d" ),

1027  ctor\_args, x.name\_, x.dims\_);

1028  }

1029  void operator()(row\_vector\_var\_decl const& x) const {

1030  std::vector<expression> ctor\_args;

1031  ctor\_args.push\_back(x.N\_);

1032  declare\_array(is\_fun\_return\_

1033  ? "Eigen::Matrix<fun\_scalar\_t\_\_,1,Eigen::Dynamic> "

1034  : ( is\_var\_

1035  ? "Eigen::Matrix<T\_\_,1,Eigen::Dynamic> "

1036  : "row\_vector\_d" ),

1037  ctor\_args, x.name\_, x.dims\_);

1038  }

1039  void operator()(matrix\_var\_decl const& x) const {

1040  std::vector<expression> ctor\_args;

1041  ctor\_args.push\_back(x.M\_);

1042  ctor\_args.push\_back(x.N\_);

1043  declare\_array(is\_fun\_return\_

1044  ? "Eigen::Matrix<fun\_scalar\_t\_\_,Eigen::Dynamic,Eigen::Dynamic> "

1045  : ( is\_var\_

1046  ? "Eigen::Matrix<T\_\_,Eigen::Dynamic,Eigen::Dynamic> "

1047  : "matrix\_d" ),

1048  ctor\_args, x.name\_, x.dims\_);

1049  }

1050  void operator()(unit\_vector\_var\_decl const& x) const {

1051  std::vector<expression> ctor\_args;

1052  ctor\_args.push\_back(x.K\_);

1053  declare\_array(is\_fun\_return\_

1054  ? "Eigen::Matrix<fun\_scalar\_t\_\_,Eigen::Dynamic,1> "

1055  : ( is\_var\_ ? "Eigen::Matrix<T\_\_,Eigen::Dynamic,1> " : "vector\_d" ),

1056  ctor\_args, x.name\_, x.dims\_);

1057  }

1058  void operator()(simplex\_var\_decl const& x) const {

1059  std::vector<expression> ctor\_args;

1060  ctor\_args.push\_back(x.K\_);

1061  declare\_array(is\_fun\_return\_

1062  ? "Eigen::Matrix<fun\_scalar\_t\_\_,Eigen::Dynamic,1> "

1063  : ( is\_var\_ ? "Eigen::Matrix<T\_\_,Eigen::Dynamic,1> " : "vector\_d"),

1064  ctor\_args, x.name\_, x.dims\_);

1065  }

1066  void operator()(ordered\_var\_decl const& x) const {

1067  std::vector<expression> ctor\_args;

1068  ctor\_args.push\_back(x.K\_);

1069  declare\_array(is\_fun\_return\_

1070  ? "Eigen::Matrix<fun\_scalar\_t\_\_,Eigen::Dynamic,1> "

1071  : ( is\_var\_ ? "Eigen::Matrix<T\_\_,Eigen::Dynamic,1> " : "vector\_d" ),

1072  ctor\_args, x.name\_, x.dims\_);

1073  }

1074  void operator()(positive\_ordered\_var\_decl const& x) const {

1075  std::vector<expression> ctor\_args;

1076  ctor\_args.push\_back(x.K\_);

1077  declare\_array(is\_fun\_return\_

1078  ? "Eigen::Matrix<fun\_scalar\_t\_\_,Eigen::Dynamic,1> "

1079  : ( is\_var\_ ? "Eigen::Matrix<T\_\_,Eigen::Dynamic,1> " : "vector\_d" ),

1080  ctor\_args, x.name\_, x.dims\_);

1081  }

1082  void operator()(cholesky\_factor\_var\_decl const& x) const {

1083  std::vector<expression> ctor\_args;

1084  ctor\_args.push\_back(x.M\_);

1085  ctor\_args.push\_back(x.N\_);

1086  declare\_array(is\_fun\_return\_

1087  ? "Eigen::Matrix<fun\_scalar\_t\_\_,Eigen::Dynamic,Eigen::Dynamic> "

1088  : ( is\_var\_

1089  ? "Eigen::Matrix<T\_\_,Eigen::Dynamic,Eigen::Dynamic> "

1090  : "matrix\_d" ),

1091  ctor\_args, x.name\_, x.dims\_);

1092  }

1093  void operator()(const cholesky\_corr\_var\_decl& x) const {

1094  std::vector<expression> ctor\_args;

1095  ctor\_args.push\_back(x.K\_);

1096  ctor\_args.push\_back(x.K\_);

1097  declare\_array(is\_var\_

1098  ? "Eigen::Matrix<T\_\_,Eigen::Dynamic,Eigen::Dynamic> "

1099  : "matrix\_d",

1100  ctor\_args, x.name\_, x.dims\_);

1101  }

1102  void operator()(cov\_matrix\_var\_decl const& x) const {

1103  std::vector<expression> ctor\_args;

1104  ctor\_args.push\_back(x.K\_);

1105  ctor\_args.push\_back(x.K\_);

1106  declare\_array(is\_fun\_return\_

1107  ? "Eigen::Matrix<fun\_scalar\_t\_\_,Eigen::Dynamic,Eigen::Dynamic> "

1108  : ( is\_var\_

1109  ? "Eigen::Matrix<T\_\_,Eigen::Dynamic,Eigen::Dynamic> "

1110  : "matrix\_d" ),

1111  ctor\_args, x.name\_, x.dims\_);

1112  }

1113  void operator()(corr\_matrix\_var\_decl const& x) const {

1114  std::vector<expression> ctor\_args;

1115  ctor\_args.push\_back(x.K\_);

1116  ctor\_args.push\_back(x.K\_);

1117  declare\_array(is\_fun\_return\_

1118  ? "Eigen::Matrix<fun\_scalar\_t\_\_,Eigen::Dynamic,Eigen::Dynamic> "

1119  : ( is\_var\_

1120  ? "Eigen::Matrix<T\_\_,Eigen::Dynamic,Eigen::Dynamic> "

1121  : "matrix\_d" ),

1122  ctor\_args, x.name\_, x.dims\_);

1123  }

1124  void generate\_type(const std::string& type,

1125  size\_t num\_dims) const {

1126  for (size\_t i = 0; i < num\_dims; ++i)

1127  o\_ << "vector<";

1128  o\_ << type;

1129  for (size\_t i = 0; i < num\_dims; ++i) {

1130  if (i > 0) o\_ << " ";

1131  o\_ << ">";

1132  }

1133  }

1134

1135  void generate\_void\_statement(const std::string& name) const {

1136  o\_ << "(void) " << name << "; // dummy to suppress unused var warning";

1137  }

1138

1139  // var\_decl -> type[0] name init\_args[0] ;

1140  // init\_args[k] -> ctor\_args if no dims left

1141  // init\_args[k] -> ( dim[k] , ( type[k+1] init\_args[k+1] ) )

1142  void generate\_init\_args(const std::string& type,

1143  const std::vector<expression>& ctor\_args,

1144  const std::vector<expression>& dims,

1145  size\_t dim) const {

1146  if (dim < dims.size()) { // more dims left

1147  o\_ << '('; // open(1)

1148  generate\_expression(dims[dim],o\_);

1149  if ((dim + 1 < dims.size()) || ctor\_args.size() > 0) {

1150  o\_ << ", ("; // open(2)

1151  generate\_type(type,dims.size() - dim - 1);

1152  generate\_init\_args(type,ctor\_args,dims,dim + 1);

1153  o\_ << ')'; // close(2)

1154  } else if (type == "var") {

1155  o\_ << ", DUMMY\_VAR\_\_";

1156  } else if (type == "int") {

1157  o\_ << ", 0";

1158  } else if (type == "double") {

1159  o\_ << ", 0.0";

1160  } else {

1161  // shouldn't hit this

1162  }

1163  o\_ << ')'; // close(1)

1164  } else {

1165  if (ctor\_args.size() == 0) { // scalar int or real

1166  if (type == "int") {

1167  o\_ << "(0)";

1168  } else if (type == "double") {

1169  o\_ << "(0.0)";

1170  } else if (type == "var") {

1171  o\_ << "(DUMMY\_VAR\_\_)";

1172  } else {

1173  // shouldn't hit this, either

1174  }

1175  }

1176  else if (ctor\_args.size() == 1) {// vector

1177  o\_ << '(';

1178  generate\_expression(ctor\_args[0],o\_);

1179  o\_ << ')';

1180  } else if (ctor\_args.size() > 1) { // matrix

1181  o\_ << '(';

1182  generate\_expression(ctor\_args[0],o\_);

1183  o\_ << ',';

1184  generate\_expression(ctor\_args[1],o\_);

1185  o\_ << ')';

1186  }

1187  }

1188  }

1189  void declare\_array(const std::string& type,

1190  const std::vector<expression>& ctor\_args,

1191  const std::string& name,

1192  const std::vector<expression>& dims) const {

1193

1194  // require double parens to counter "most vexing parse" problem

1195

1196  generate\_indent(indents\_,o\_);

1197  generate\_type(type,dims.size());

1198  o\_ << ' ' << name;

1199  generate\_init\_args(type,ctor\_args,dims,0);

1200  o\_ << ';' << EOL;

1201  if (dims.size() == 0) {

1202  generate\_indent(indents\_,o\_);

1203  generate\_void\_statement(name);

1204  o\_ << EOL;

1205  }

1206  if (type == "Eigen::Matrix<T\_\_,Eigen::Dynamic,Eigen::Dynamic> "

1207  || type == "Eigen::Matrix<T\_\_,1,Eigen::Dynamic> "

1208  || type == "Eigen::Matrix<T\_\_,Eigen::Dynamic,1> ") {

1209  generate\_indent(indents\_,o\_);

1210  o\_ << "stan::math::fill(" << name << ",DUMMY\_VAR\_\_);" << EOL;

1211  }

1212  }

1213  };

1214

1215  void generate\_local\_var\_decls(const std::vector<var\_decl>& vs,

1216  int indent,

1217  std::ostream& o,

1218  bool is\_var,

1219  bool is\_fun\_return) {

1220  local\_var\_decl\_visgen vis(indent,is\_var,is\_fun\_return,o);

1221  for (size\_t i = 0; i < vs.size(); ++i)

1222  boost::apply\_visitor(vis,vs[i].decl\_);

1223  }

1224

1225

1226

1227  struct generate\_local\_var\_init\_nan\_visgen : public visgen {

1228  const bool declare\_vars\_;

1229  const bool is\_var\_;

1230  const bool is\_fun\_return\_;

1231  const int indent\_;

1232  generate\_local\_var\_init\_nan\_visgen(bool declare\_vars,

1233  bool is\_var,

1234  bool is\_fun\_return,

1235  int indent,

1236  std::ostream& o)

1237  : visgen(o),

1238  declare\_vars\_(declare\_vars),

1239  is\_var\_(is\_var),

1240  is\_fun\_return\_(is\_fun\_return),

1241  indent\_(indent) {

1242  }

1243  void operator()(const nil& /\*x\*/) const {

1244  // no-op

1245  }

1246  void operator()(const int\_var\_decl& x) const {

1247  // no-op; ints need no init to prevent crashes and no NaN available

1248  }

1249  void operator()(const double\_var\_decl& x) const {

1250  generate\_init(x);

1251  }

1252  void operator()(const vector\_var\_decl& x) const {

1253  generate\_init(x);

1254  }

1255  void operator()(const row\_vector\_var\_decl& x) const {

1256  generate\_init(x);

1257  }

1258  void operator()(const matrix\_var\_decl& x) const {

1259  generate\_init(x);

1260  }

1261  void operator()(const unit\_vector\_var\_decl& x) const {

1262  generate\_init(x);

1263  }

1264  void operator()(const simplex\_var\_decl& x) const {

1265  generate\_init(x);

1266  }

1267  void operator()(const ordered\_var\_decl& x) const {

1268  generate\_init(x);

1269  }

1270  void operator()(const positive\_ordered\_var\_decl& x) const {

1271  generate\_init(x);

1272  }

1273  void operator()(const cholesky\_factor\_var\_decl& x) const {

1274  generate\_init(x);

1275  }

1276  void operator()(const cholesky\_corr\_var\_decl& x) const {

1277  generate\_init(x);

1278  }

1279  void operator()(const cov\_matrix\_var\_decl& x) const {

1280  generate\_init(x);

1281  }

1282  void operator()(const corr\_matrix\_var\_decl& x) const {

1283  generate\_init(x);

1284  }

1285  template <typename T>

1286  void generate\_init(const T& x) const {

1287  generate\_indent(indent\_,o\_);

1288  o\_ << "stan::math::initialize(" << x.name\_ << ", "

1289  << (is\_var\_ ? "DUMMY\_VAR\_\_" : "std::numeric\_limits<double>::quiet\_NaN()")

1290  << ");"

1291  << EOL;

1292  }

1293  };

1294

1295  void generate\_local\_var\_init\_nan(const std::vector<var\_decl>& vs,

1296  int indent,

1297  std::ostream& o,

1298  bool is\_var,

1299  bool is\_fun\_return) {

1300  generate\_local\_var\_init\_nan\_visgen vis(indent,is\_var,is\_fun\_return,indent,o);

1301  for (size\_t i = 0; i < vs.size(); ++i)

1302  boost::apply\_visitor(vis,vs[i].decl\_);

1303  }

1304

1305

1306  // see member\_var\_decl\_visgen cut & paste

1307  struct generate\_init\_vars\_visgen : public visgen {

1308  int indent\_;

1309  generate\_init\_vars\_visgen(int indent,

1310  std::ostream& o)

1311  : visgen(o),

1312  indent\_(indent) {

1313  }

1314  void operator()(nil const& /\*x\*/) const { }

1315  void operator()(int\_var\_decl const& x) const {

1316  generate\_indent(indent\_,o\_);

1317  o\_ << "stan::math::fill(" << x.name\_ << ",DUMMY\_VAR\_\_);" << EOL;

1318  }

1319  void operator()(double\_var\_decl const& x) const {

1320  generate\_indent(indent\_,o\_);

1321  o\_ << "stan::math::fill(" << x.name\_ << ",DUMMY\_VAR\_\_);" << EOL;

1322  }

1323  void operator()(vector\_var\_decl const& x) const {

1324  generate\_indent(indent\_,o\_);

1325  o\_ << "stan::math::fill(" << x.name\_ << ",DUMMY\_VAR\_\_);" << EOL;

1326  }

1327  void operator()(row\_vector\_var\_decl const& x) const {

1328  generate\_indent(indent\_,o\_);

1329  o\_ << "stan::math::fill(" << x.name\_ << ",DUMMY\_VAR\_\_);" << EOL;

1330  }

1331  void operator()(matrix\_var\_decl const& x) const {

1332  generate\_indent(indent\_,o\_);

1333  o\_ << "stan::math::fill(" << x.name\_ << ",DUMMY\_VAR\_\_);" << EOL;

1334  }

1335  void operator()(unit\_vector\_var\_decl const& x) const {

1336  generate\_indent(indent\_,o\_);

1337  o\_ << "stan::math::fill(" << x.name\_ << ",DUMMY\_VAR\_\_);" << EOL;

1338  }

1339  void operator()(simplex\_var\_decl const& x) const {

1340  generate\_indent(indent\_,o\_);

1341  o\_ << "stan::math::fill(" << x.name\_ << ",DUMMY\_VAR\_\_);" << EOL;

1342  }

1343  void operator()(ordered\_var\_decl const& x) const {

1344  generate\_indent(indent\_,o\_);

1345  o\_ << "stan::math::fill(" << x.name\_ << ",DUMMY\_VAR\_\_);" << EOL;

1346  }

1347  void operator()(positive\_ordered\_var\_decl const& x) const {

1348  generate\_indent(indent\_,o\_);

1349  o\_ << "stan::math::fill(" << x.name\_ << ",DUMMY\_VAR\_\_);" << EOL;

1350  }

1351  void operator()(cholesky\_factor\_var\_decl const& x) const {

1352  generate\_indent(indent\_,o\_);

1353  o\_ << "stan::math::fill(" << x.name\_ << ",DUMMY\_VAR\_\_);" << EOL;

1354  }

1355  void operator()(cholesky\_corr\_var\_decl const& x) const {

1356  generate\_indent(indent\_,o\_);

1357  o\_ << "stan::math::fill(" << x.name\_ << ",DUMMY\_VAR\_\_);" << EOL;

1358  }

1359  void operator()(cov\_matrix\_var\_decl const& x) const {

1360  generate\_indent(indent\_,o\_);

1361  o\_ << "stan::math::fill(" << x.name\_ << ",DUMMY\_VAR\_\_);" << EOL;

1362  }

1363  void operator()(corr\_matrix\_var\_decl const& x) const {

1364  generate\_indent(indent\_,o\_);

1365  o\_ << "stan::math::fill(" << x.name\_ << ",DUMMY\_VAR\_\_);" << EOL;

1366  }

1367  };

1368

1369  void generate\_init\_vars(const std::vector<var\_decl>& vs,

1370  int indent,

1371  std::ostream& o) {

1372  generate\_init\_vars\_visgen vis(indent,o);

1373  o << EOL;

1374  generate\_comment("initialized transformed params to avoid seg fault on val access",

1375  indent,o);

1376  for (size\_t i = 0; i < vs.size(); ++i)

1377  boost::apply\_visitor(vis,vs[i].decl\_);

1378  }

1379

1380

1381  struct validate\_transformed\_params\_visgen : public visgen {

1382  int indents\_;

1383  validate\_transformed\_params\_visgen(int indents,

1384  std::ostream& o)

1385  : visgen(o),

1386  indents\_(indents)

1387  { }

1388  void operator()(nil const& /\*x\*/) const { }

1389  void operator()(int\_var\_decl const& x) const {

1390  std::vector<expression> dims(x.dims\_);

1391  validate\_array(x.name\_,dims,0);

1392  }

1393  void operator()(double\_var\_decl const& x) const {

1394  std::vector<expression> dims(x.dims\_);

1395  validate\_array(x.name\_,dims,0);

1396  }

1397  void operator()(vector\_var\_decl const& x) const {

1398  std::vector<expression> dims(x.dims\_);

1399  dims.push\_back(x.M\_);

1400  validate\_array(x.name\_,dims,1);

1401  }

1402  void operator()(unit\_vector\_var\_decl const& x) const {

1403  std::vector<expression> dims(x.dims\_);

1404  dims.push\_back(x.K\_);

1405  validate\_array(x.name\_,dims,1);

1406  }

1407  void operator()(simplex\_var\_decl const& x) const {

1408  std::vector<expression> dims(x.dims\_);

1409  dims.push\_back(x.K\_);

1410  validate\_array(x.name\_,dims,1);

1411  }

1412  void operator()(ordered\_var\_decl const& x) const {

1413  std::vector<expression> dims(x.dims\_);

1414  dims.push\_back(x.K\_);

1415  validate\_array(x.name\_,dims,1);

1416  }

1417  void operator()(positive\_ordered\_var\_decl const& x) const {

1418  std::vector<expression> dims(x.dims\_);

1419  dims.push\_back(x.K\_);

1420  validate\_array(x.name\_,dims,1);

1421  }

1422  void operator()(row\_vector\_var\_decl const& x) const {

1423  std::vector<expression> dims(x.dims\_);

1424  dims.push\_back(x.N\_);

1425  validate\_array(x.name\_,dims,1);

1426  }

1427  void operator()(matrix\_var\_decl const& x) const {

1428  std::vector<expression> dims(x.dims\_);

1429  dims.push\_back(x.M\_);

1430  dims.push\_back(x.N\_);

1431  validate\_array(x.name\_,dims,2);

1432  }

1433  void operator()(cholesky\_factor\_var\_decl const& x) const {

1434  std::vector<expression> dims(x.dims\_);

1435  dims.push\_back(x.M\_);

1436  dims.push\_back(x.N\_);

1437  validate\_array(x.name\_,dims,2);

1438  }

1439  void operator()(cholesky\_corr\_var\_decl const& x) const {

1440  std::vector<expression> dims(x.dims\_);

1441  dims.push\_back(x.K\_);

1442  dims.push\_back(x.K\_);

1443  validate\_array(x.name\_,dims,2);

1444  }

1445  void operator()(cov\_matrix\_var\_decl const& x) const {

1446  std::vector<expression> dims(x.dims\_);

1447  dims.push\_back(x.K\_);

1448  dims.push\_back(x.K\_);

1449  validate\_array(x.name\_,dims,2);

1450  }

1451  void operator()(corr\_matrix\_var\_decl const& x) const {

1452  std::vector<expression> dims(x.dims\_);

1453  dims.push\_back(x.K\_);

1454  dims.push\_back(x.K\_);

1455  validate\_array(x.name\_,dims,2);

1456  }

1457  void validate\_array(const std::string& name,

1458  const std::vector<expression>& dims,

1459  size\_t matrix\_dims) const {

1460

1461  size\_t non\_matrix\_dims = dims.size() - matrix\_dims;

1462

1463  for (size\_t k = 0; k < dims.size(); ++k) {

1464  generate\_indent(indents\_ + k,o\_);

1465  o\_ << "for (int i" << k << "\_\_ = 0; i" << k << "\_\_ < ";

1466  generate\_expression(dims[k],o\_);

1467  o\_ << "; ++i" << k << "\_\_) {" << EOL;

1468  }

1469

1470  generate\_indent(indents\_ + dims.size(), o\_);

1471  o\_ << "if (stan::math::is\_uninitialized(" << name;

1472  for (size\_t k = 0; k < non\_matrix\_dims; ++k)

1473  o\_ << "[i" << k << "\_\_]";

1474  if (matrix\_dims > 0) {

1475  o\_ << "(i" << non\_matrix\_dims << "\_\_";

1476  if (matrix\_dims > 1)

1477  o\_ << ",i" << (non\_matrix\_dims + 1) << "\_\_";

1478  o\_ << ')';

1479  }

1480  o\_ << ")) {" << EOL;

1481  generate\_indent(indents\_ + dims.size() + 1, o\_);

1482  o\_ << "std::stringstream msg\_\_;" << EOL;

1483  generate\_indent(indents\_ + dims.size() + 1, o\_);

1484  o\_ << "msg\_\_ << \"Undefined transformed parameter: "

1485  << name << "\"";

1486  for (size\_t k = 0; k < dims.size(); ++k) {

1487  o\_ << " << '['";

1488  o\_ << " << i" << k << "\_\_";

1489  o\_ << " << ']'";

1490  }

1491  o\_ << ';' << EOL;

1492  generate\_indent(indents\_ + dims.size() + 1, o\_);

1493  o\_ << "throw std::runtime\_error(msg\_\_.str());" << EOL;

1494

1495  generate\_indent(indents\_ + dims.size(), o\_);

1496  o\_ << "}" << EOL;

1497  for (size\_t k = 0; k < dims.size(); ++k) {

1498  generate\_indent(indents\_ + dims.size() - k - 1, o\_);

1499  o\_ << "}" << EOL;

1500  }

1501  }

1502  };

1503

1504  void generate\_validate\_transformed\_params(const std::vector<var\_decl>& vs,

1505  int indent,

1506  std::ostream& o) {

1507  generate\_comment("validate transformed parameters",indent,o);

1508  validate\_transformed\_params\_visgen vis(indent,o);

1509  for (size\_t i = 0; i < vs.size(); ++i)

1510  boost::apply\_visitor(vis,vs[i].decl\_);

1511  o << EOL;

1512  }

1513

1514  void generate\_statement(statement const& s, int indent, std::ostream& o,

1515  bool include\_sampling, bool is\_var,

1516  bool is\_fun\_return);

1517

1518  struct statement\_visgen : public visgen {

1519  size\_t indent\_;

1520  bool include\_sampling\_;

1521  bool is\_var\_;

1522  bool is\_fun\_return\_;

1523  statement\_visgen(size\_t indent,

1524  bool include\_sampling,

1525  bool is\_var,

1526  bool is\_fun\_return,

1527  std::ostream& o)

1528  : visgen(o),

1529  indent\_(indent),

1530  include\_sampling\_(include\_sampling),

1531  is\_var\_(is\_var),

1532  is\_fun\_return\_(is\_fun\_return) {

1533  }

1534  void operator()(nil const& /\*x\*/) const {

1535  }

1536  void operator()(assignment const& x) const {

1537  generate\_indent(indent\_,o\_);

1538  o\_ << "stan::math::assign(";

1539  generate\_indexed\_expr<true>(x.var\_dims\_.name\_,

1540  x.var\_dims\_.dims\_,

1541  x.var\_type\_.base\_type\_,

1542  x.var\_type\_.dims\_.size(),

1543  o\_);

1544  o\_ << ", ";

1545  generate\_expression(x.expr\_,o\_);

1546  o\_ << ");" << EOL;

1547  }

1548  void operator()(expression const& x) const {

1549  generate\_indent(indent\_,o\_);

1550  generate\_expression(x,o\_);

1551  o\_ << ";" << EOL;

1552  }

1553  void operator()(sample const& x) const {

1554  if (!include\_sampling\_) return;

1555  generate\_indent(indent\_,o\_);

1556  o\_ << "lp\_accum\_\_.add(" << x.dist\_.family\_ << "\_log<propto\_\_>(";

1557  generate\_expression(x.expr\_,o\_);

1558  for (size\_t i = 0; i < x.dist\_.args\_.size(); ++i) {

1559  o\_ << ", ";

1560  generate\_expression(x.dist\_.args\_[i],o\_);

1561  }

1562  if (is\_user\_defined\_prob\_function(x.dist\_.family\_ + "\_log",

1563  x.expr\_,

1564  x.dist\_.args\_)) {

1565  o\_ << ", pstream\_\_";

1566  }

1567  o\_ << "));" << EOL;

1568  // rest of impl is for truncation

1569  // generate bounds test

1570  if (x.truncation\_.has\_low()) {

1571  generate\_indent(indent\_,o\_);

1572  o\_ << "if (";

1573  generate\_expression(x.expr\_,o\_);

1574  o\_ << " < ";

1575  generate\_expression(x.truncation\_.low\_.expr\_,o\_); // low

1576  // bound

1577  o\_ << ") lp\_accum\_\_.add(-std::numeric\_limits<double>::infinity());" << EOL;

1578  }

1579  if (x.truncation\_.has\_high()) {

1580  generate\_indent(indent\_,o\_);

1581  if (x.truncation\_.has\_low()) o\_ << "else ";

1582  o\_ << "if (";

1583  generate\_expression(x.expr\_,o\_);

1584  o\_ << " > ";

1585  generate\_expression(x.truncation\_.high\_.expr\_,o\_); // low

1586  // bound

1587  o\_ << ") lp\_accum\_\_.add(-std::numeric\_limits<double>::infinity());" << EOL;

1588  }

1589  // generate log denominator for case where bounds test pass

1590  if (x.truncation\_.has\_low() || x.truncation\_.has\_high()) {

1591  generate\_indent(indent\_,o\_);

1592  o\_ << "else ";

1593  }

1594  if (x.truncation\_.has\_low() && x.truncation\_.has\_high()) {

1595  // T[L,U]: -log\_diff\_exp(Dist\_cdf\_log(U|params),Dist\_cdf\_log(L|Params))

1596  o\_ << "lp\_accum\_\_.add(-log\_diff\_exp(";

1597  o\_ << x.dist\_.family\_ << "\_cdf\_log(";

1598  generate\_expression(x.truncation\_.high\_.expr\_,o\_);

1599  for (size\_t i = 0; i < x.dist\_.args\_.size(); ++i) {

1600  o\_ << ", ";

1601  generate\_expression(x.dist\_.args\_[i],o\_);

1602  }

1603  o\_ << "), " << x.dist\_.family\_ << "\_cdf\_log(";

1604  generate\_expression(x.truncation\_.low\_.expr\_,o\_);

1605  for (size\_t i = 0; i < x.dist\_.args\_.size(); ++i) {

1606  o\_ << ", ";

1607  generate\_expression(x.dist\_.args\_[i],o\_);

1608  }

1609  o\_ << ")));" << EOL;

1610  } else if (!x.truncation\_.has\_low() && x.truncation\_.has\_high()) {

1611  // T[,U]; -Dist\_cdf\_log(U)

1612  o\_ << "lp\_accum\_\_.add(-";

1613  o\_ << x.dist\_.family\_ << "\_cdf\_log(";

1614  generate\_expression(x.truncation\_.high\_.expr\_,o\_);

1615  for (size\_t i = 0; i < x.dist\_.args\_.size(); ++i) {

1616  o\_ << ", ";

1617  generate\_expression(x.dist\_.args\_[i],o\_);

1618  }

1619  o\_ << "));" << EOL;

1620  } else if (x.truncation\_.has\_low() && !x.truncation\_.has\_high()) {

1621  // T[L,]: -Dist\_ccdf\_log(L)

1622  o\_ << "lp\_accum\_\_.add(-";

1623  o\_ << x.dist\_.family\_ << "\_ccdf\_log(";

1624  generate\_expression(x.truncation\_.low\_.expr\_,o\_);

1625  for (size\_t i = 0; i < x.dist\_.args\_.size(); ++i) {

1626  o\_ << ", ";

1627  generate\_expression(x.dist\_.args\_[i],o\_);

1628  }

1629  o\_ << "));" << EOL;

1630  }

1631  }

1632  void operator()(const increment\_log\_prob\_statement& x) const {

1633  generate\_indent(indent\_,o\_);

1634  o\_ << "lp\_accum\_\_.add(";

1635  generate\_expression(x.log\_prob\_,o\_);

1636  o\_ << ");" << EOL;

1637  }

1638  void operator()(const statements& x) const {

1639  bool has\_local\_vars = x.local\_decl\_.size() > 0;

1640  size\_t indent = has\_local\_vars ? (indent\_ + 1) : indent\_;

1641  if (has\_local\_vars) {

1642  generate\_indent(indent\_,o\_);

1643  o\_ << "{" << EOL;

1644  generate\_local\_var\_decls(x.local\_decl\_,indent,o\_,

1645  is\_var\_,is\_fun\_return\_);

1646  generate\_local\_var\_init\_nan(x.local\_decl\_,indent,o\_,

1647  is\_var\_,is\_fun\_return\_);

1648  }

1649

1650  for (size\_t i = 0; i < x.statements\_.size(); ++i)

1651  generate\_statement(x.statements\_[i],indent,o\_,include\_sampling\_,is\_var\_,

1652  is\_fun\_return\_);

1653

1654  if (has\_local\_vars) {

1655  generate\_indent(indent\_,o\_);

1656  o\_ << "}" << EOL;

1657  }

1658  }

1659  void operator()(const print\_statement& ps) const {

1660  generate\_indent(indent\_,o\_);

1661  o\_ << "if (pstream\_\_) {" << EOL;

1662  for (size\_t i = 0; i < ps.printables\_.size(); ++i) {

1663  generate\_indent(indent\_ + 1,o\_);

1664  o\_ << "stan\_print(pstream\_\_,";

1665  generate\_printable(ps.printables\_[i],o\_);

1666  o\_ << ");" << EOL;

1667  }

1668  generate\_indent(indent\_ + 1,o\_);

1669  o\_ << "\*pstream\_\_ << std::endl;" << EOL;

1670  generate\_indent(indent\_,o\_);

1671  o\_ << '}' << EOL;

1672  }

1673  void operator()(const reject\_statement& ps) const {

1674  generate\_indent(indent\_,o\_);

1675  o\_ << "std::stringstream errmsg\_stream\_\_;" << EOL;

1676  for (size\_t i = 0; i < ps.printables\_.size(); ++i) {

1677  generate\_indent(indent\_,o\_);

1678  o\_ << "errmsg\_stream\_\_ << ";

1679  generate\_printable(ps.printables\_[i],o\_);

1680  o\_ << ";" << EOL;

1681  }

1682  generate\_indent(indent\_,o\_);

1683  o\_ << "throw std::domain\_error(errmsg\_stream\_\_.str());" << EOL;

1684  }

1685  void operator()(const return\_statement& rs) const {

1686  generate\_indent(indent\_,o\_);

1687  o\_ << "return ";

1688  if (!rs.return\_value\_.expression\_type().is\_ill\_formed()

1689  && !rs.return\_value\_.expression\_type().is\_void()) {

1690  o\_ << "stan::math::promote\_scalar<fun\_return\_scalar\_t\_\_>(";

1691  generate\_expression(rs.return\_value\_, o\_);

1692  o\_ << ")";

1693  }

1694  o\_ << ";" << EOL;

1695  }

1696  void operator()(const for\_statement& x) const {

1697  generate\_indent(indent\_,o\_);

1698  o\_ << "for (int " << x.variable\_ << " = ";

1699  generate\_expression(x.range\_.low\_,o\_);

1700  o\_ << "; " << x.variable\_ << " <= ";

1701  generate\_expression(x.range\_.high\_,o\_);

1702  o\_ << "; ++" << x.variable\_ << ") {" << EOL;

1703  generate\_statement(x.statement\_, indent\_ + 1, o\_, include\_sampling\_,

1704  is\_var\_,is\_fun\_return\_);

1705  generate\_indent(indent\_,o\_);

1706  o\_ << "}" << EOL;

1707  }

1708  void operator()(const while\_statement& x) const {

1709  generate\_indent(indent\_,o\_);

1710  o\_ << "while (as\_bool(";

1711  generate\_expression(x.condition\_,o\_);

1712  o\_ << ")) {" << EOL;

1713  generate\_statement(x.body\_, indent\_+1, o\_, include\_sampling\_,

1714  is\_var\_,is\_fun\_return\_);

1715  generate\_indent(indent\_,o\_);

1716  o\_ << "}" << EOL;

1717  }

1718  void operator()(const conditional\_statement& x) const {

1719  for (size\_t i = 0; i < x.conditions\_.size(); ++i) {

1720  if (i == 0)

1721  generate\_indent(indent\_,o\_);

1722  else

1723  o\_ << " else ";

1724  o\_ << "if (as\_bool(";

1725  generate\_expression(x.conditions\_[i],o\_);

1726  o\_ << ")) {" << EOL;

1727  generate\_statement(x.bodies\_[i], indent\_ + 1, o\_, include\_sampling\_,

1728  is\_var\_, is\_fun\_return\_);

1729  generate\_indent(indent\_,o\_);

1730  o\_ << '}';

1731  }

1732  if (x.bodies\_.size() > x.conditions\_.size()) {

1733  o\_ << " else {" << EOL;

1734  generate\_statement(x.bodies\_[x.bodies\_.size()-1], indent\_ + 1,

1735  o\_, include\_sampling\_,

1736  is\_var\_,is\_fun\_return\_);

1737  generate\_indent(indent\_,o\_);

1738  o\_ << '}';

1739  }

1740  o\_ << EOL;

1741  }

1742  void operator()(const no\_op\_statement& /\*x\*/) const {

1743  }

1744  };

1745

1746  void generate\_statement(const statement& s,

1747  int indent,

1748  std::ostream& o,

1749  bool include\_sampling,

1750  bool is\_var,

1751  bool is\_fun\_return) {

1752  statement\_visgen vis(indent,include\_sampling,is\_var,is\_fun\_return, o);

1753  boost::apply\_visitor(vis,s.statement\_);

1754  }

1755

1756  void generate\_statements(const std::vector<statement>& ss,

1757  int indent,

1758  std::ostream& o,

1759  bool include\_sampling,

1760  bool is\_var,

1761  bool is\_fun\_return) {

1762  statement\_visgen vis(indent,include\_sampling,is\_var,is\_fun\_return,o);

1763  for (size\_t i = 0; i < ss.size(); ++i)

1764  boost::apply\_visitor(vis,ss[i].statement\_);

1765  }

1766

1767

1768  void generate\_log\_prob(program const& p,

1769  std::ostream& o) {

1770

1771

1772  o << EOL;

1773  o << INDENT << "template <bool propto\_\_, bool jacobian\_\_, typename T\_\_>" << EOL;

1774  o << INDENT << "T\_\_ log\_prob(vector<T\_\_>& params\_r\_\_," << EOL;

1775  o << INDENT << " vector<int>& params\_i\_\_," << EOL;

1776  o << INDENT << " std::ostream\* pstream\_\_ = 0) const {" << EOL2;

1777

1778  // use this dummy for inits

1779  o << INDENT2 << "T\_\_ DUMMY\_VAR\_\_(std::numeric\_limits<double>::quiet\_NaN());" << EOL;

1780  o << INDENT2 << "(void) DUMMY\_VAR\_\_; // suppress unused var warning" << EOL2;

1781

1782  o << INDENT2 << "T\_\_ lp\_\_(0.0);" << EOL;

1783  o << INDENT2 << "stan::math::accumulator<T\_\_> lp\_accum\_\_;" << EOL2;

1784

1785  bool is\_var = true;

1786  bool is\_fun\_return = false;

1787

1788  generate\_comment("model parameters",2,o);

1789  generate\_local\_var\_inits(p.parameter\_decl\_,is\_var,true,o);

1790  o << EOL;

1791

1792  generate\_comment("transformed parameters",2,o);

1793  generate\_local\_var\_decls(p.derived\_decl\_.first,2,o,is\_var,is\_fun\_return);

1794  generate\_init\_vars(p.derived\_decl\_.first,2,o);

1795

1796  o << EOL;

1797  bool include\_sampling = true;

1798  generate\_statements(p.derived\_decl\_.second,2,o,include\_sampling,

1799  is\_var,is\_fun\_return);

1800  o << EOL;

1801

1802  generate\_validate\_transformed\_params(p.derived\_decl\_.first,2,o);

1803  o << INDENT2

1804  << "const char\* function\_\_ = \"validate transformed params %1%\";"

1805  << EOL;

1806  o << INDENT2

1807  << "(void) function\_\_; // dummy to suppress unused var warning"

1808  << EOL;

1809

1810  generate\_validate\_var\_decls(p.derived\_decl\_.first,2,o);

1811

1812  generate\_comment("model body",2,o);

1813  generate\_statement(p.statement\_,2,o,include\_sampling,

1814  is\_var,is\_fun\_return);

1815  o << EOL;

1816  o << INDENT2 << "lp\_accum\_\_.add(lp\_\_);" << EOL;

1817  o << INDENT2 << "return lp\_accum\_\_.sum();" << EOL2;

1818  o << INDENT << "} // log\_prob()" << EOL2;

1819

1820  o << INDENT << "template <bool propto, bool jacobian, typename T\_>" << EOL;

1821  o << INDENT << "T\_ log\_prob(Eigen::Matrix<T\_,Eigen::Dynamic,1>& params\_r," << EOL;

1822  o << INDENT << " std::ostream\* pstream = 0) const {" << EOL;

1823  o << INDENT << " std::vector<T\_> vec\_params\_r;" << EOL;

1824  o << INDENT << " vec\_params\_r.reserve(params\_r.size());" << EOL;

1825  o << INDENT << " for (int i = 0; i < params\_r.size(); ++i)" << EOL;

1826  o << INDENT << " vec\_params\_r.push\_back(params\_r(i));" << EOL;

1827  o << INDENT << " std::vector<int> vec\_params\_i;" << EOL;

1828  o << INDENT << " return log\_prob<propto,jacobian,T\_>(vec\_params\_r, vec\_params\_i, pstream);" << EOL;

1829  o << INDENT << "}" << EOL2;

1830  }

1831

1832  struct dump\_member\_var\_visgen : public visgen {

1833  var\_resizing\_visgen var\_resizer\_;

1834  var\_size\_validating\_visgen var\_size\_validator\_;

1835  dump\_member\_var\_visgen(std::ostream& o)

1836  : visgen(o),

1837  var\_resizer\_(var\_resizing\_visgen(o)),

1838  var\_size\_validator\_(var\_size\_validating\_visgen(o,"data initialization")) {

1839  }

1840  void operator()(nil const& /\*x\*/) const { } // dummy

1841  void operator()(int\_var\_decl const& x) const {

1842  std::vector<expression> dims = x.dims\_;

1843  var\_size\_validator\_(x);

1844  var\_resizer\_(x);

1845  o\_ << INDENT2 << "vals\_i\_\_ = context\_\_.vals\_i(\"" << x.name\_ << "\");" << EOL;

1846  o\_ << INDENT2 << "pos\_\_ = 0;" << EOL;

1847  size\_t indentation = 1;

1848  for (size\_t dim\_up = 0U; dim\_up < dims.size(); ++dim\_up) {

1849  size\_t dim = dims.size() - dim\_up - 1U;

1850  ++indentation;

1851  generate\_indent(indentation,o\_);

1852  o\_ << "size\_t " << x.name\_ << "\_limit\_" << dim << "\_\_ = ";

1853  generate\_expression(dims[dim],o\_);

1854  o\_ << ";" << EOL;

1855  generate\_indent(indentation,o\_);

1856  o\_ << "for (size\_t i\_" << dim << "\_\_ = 0; i\_"

1857  << dim << "\_\_ < " << x.name\_ << "\_limit\_" << dim

1858  << "\_\_; ++i\_" << dim << "\_\_) {" << EOL;

1859  }

1860  generate\_indent(indentation+1,o\_);

1861  o\_ << x.name\_;

1862  for (size\_t dim = 0; dim < dims.size(); ++dim)

1863  o\_ << "[i\_" << dim << "\_\_]";

1864  o\_ << " = vals\_i\_\_[pos\_\_++];" << EOL;

1865  for (size\_t dim = 0; dim < dims.size(); ++dim) {

1866  generate\_indent(dims.size() + 1 - dim,o\_);

1867  o\_ << "}" << EOL;

1868  }

1869  }

1870  // minor changes to int\_var\_decl

1871  void operator()(double\_var\_decl const& x) const {

1872  std::vector<expression> dims = x.dims\_;

1873  var\_size\_validator\_(x);

1874  var\_resizer\_(x);

1875  o\_ << INDENT2 << "vals\_r\_\_ = context\_\_.vals\_r(\"" << x.name\_ << "\");" << EOL;

1876  o\_ << INDENT2 << "pos\_\_ = 0;" << EOL;

1877  size\_t indentation = 1;

1878  for (size\_t dim\_up = 0U; dim\_up < dims.size(); ++dim\_up) {

1879  size\_t dim = dims.size() - dim\_up - 1U;

1880  ++indentation;

1881  generate\_indent(indentation,o\_);

1882  o\_ << "size\_t " << x.name\_ << "\_limit\_" << dim << "\_\_ = ";

1883  generate\_expression(dims[dim],o\_);

1884  o\_ << ";" << EOL;

1885  generate\_indent(indentation,o\_);

1886  o\_ << "for (size\_t i\_" << dim << "\_\_ = 0; i\_" << dim << "\_\_ < " << x.name\_ << "\_limit\_" << dim << "\_\_; ++i\_" << dim << "\_\_) {" << EOL;

1887  }

1888  generate\_indent(indentation+1,o\_);

1889  o\_ << x.name\_;

1890  for (size\_t dim = 0; dim < dims.size(); ++dim)

1891  o\_ << "[i\_" << dim << "\_\_]";

1892  o\_ << " = vals\_r\_\_[pos\_\_++];" << EOL;

1893  for (size\_t dim = 0; dim < dims.size(); ++dim) {

1894  generate\_indent(dims.size() + 1 - dim,o\_);

1895  o\_ << "}" << EOL;

1896  }

1897  }

1898  // extra outer loop around double\_var\_decl

1899  void operator()(vector\_var\_decl const& x) const {

1900  std::vector<expression> dims = x.dims\_;

1901  var\_resizer\_(x);

1902  var\_size\_validator\_(x);

1903  o\_ << INDENT2 << "vals\_r\_\_ = context\_\_.vals\_r(\"" << x.name\_ << "\");" << EOL;

1904  o\_ << INDENT2 << "pos\_\_ = 0;" << EOL;

1905  o\_ << INDENT2 << "size\_t " << x.name\_ << "\_i\_vec\_lim\_\_ = ";

1906  generate\_expression(x.M\_,o\_);

1907  o\_ << ";" << EOL;

1908  o\_ << INDENT2 << "for (size\_t " << "i\_vec\_\_ = 0; " << "i\_vec\_\_ < " << x.name\_ << "\_i\_vec\_lim\_\_; ++i\_vec\_\_) {" << EOL;

1909  size\_t indentation = 2;

1910  for (size\_t dim\_up = 0U; dim\_up < dims.size(); ++dim\_up) {

1911  size\_t dim = dims.size() - dim\_up - 1U;

1912  ++indentation;

1913  generate\_indent(indentation,o\_);

1914  o\_ << "size\_t " << x.name\_ << "\_limit\_" << dim << "\_\_ = ";

1915  generate\_expression(dims[dim],o\_);

1916  o\_ << ";" << EOL;

1917  generate\_indent(indentation,o\_);

1918  o\_ << "for (size\_t i\_" << dim << "\_\_ = 0; i\_" << dim << "\_\_ < " << x.name\_ << "\_limit\_" << dim << "\_\_; ++i\_" << dim << "\_\_) {" << EOL;

1919  }

1920  generate\_indent(indentation+1,o\_);

1921  o\_ << x.name\_;

1922  for (size\_t dim = 0; dim < dims.size(); ++dim)

1923  o\_ << "[i\_" << dim << "\_\_]";

1924  o\_ << "[i\_vec\_\_]";

1925  o\_ << " = vals\_r\_\_[pos\_\_++];" << EOL;

1926  for (size\_t dim = 0; dim < dims.size(); ++dim) {

1927  generate\_indent(dims.size() + 2 - dim,o\_);

1928  o\_ << "}" << EOL;

1929  }

1930  o\_ << INDENT2 << "}" << EOL;

1931  }

1932  // change variable name from vector\_var\_decl

1933  void operator()(row\_vector\_var\_decl const& x) const {

1934  std::vector<expression> dims = x.dims\_;

1935  var\_size\_validator\_(x);

1936  var\_resizer\_(x);

1937  o\_ << INDENT2 << "vals\_r\_\_ = context\_\_.vals\_r(\"" << x.name\_ << "\");" << EOL;

1938  o\_ << INDENT2 << "pos\_\_ = 0;" << EOL;

1939  o\_ << INDENT2 << "size\_t " << x.name\_ << "\_i\_vec\_lim\_\_ = ";

1940  generate\_expression(x.N\_,o\_);

1941  o\_ << ";" << EOL;

1942  o\_ << INDENT2 << "for (size\_t " << "i\_vec\_\_ = 0; " << "i\_vec\_\_ < " << x.name\_ << "\_i\_vec\_lim\_\_; ++i\_vec\_\_) {" << EOL;

1943  size\_t indentation = 2;

1944  for (size\_t dim\_up = 0U; dim\_up < dims.size(); ++dim\_up) {

1945  size\_t dim = dims.size() - dim\_up - 1U;

1946  ++indentation;

1947  generate\_indent(indentation,o\_);

1948  o\_ << "size\_t " << x.name\_ << "\_limit\_" << dim << "\_\_ = ";

1949  generate\_expression(dims[dim],o\_);

1950  o\_ << ";" << EOL;

1951  generate\_indent(indentation,o\_);

1952  o\_ << "for (size\_t i\_" << dim << "\_\_ = 0; i\_" << dim << "\_\_ < " << x.name\_ << "\_limit\_" << dim << "\_\_; ++i\_" << dim << "\_\_) {" << EOL;

1953  }

1954  generate\_indent(indentation+1,o\_);

1955  o\_ << x.name\_;

1956  for (size\_t dim = 0; dim < dims.size(); ++dim)

1957  o\_ << "[i\_" << dim << "\_\_]";

1958  o\_ << "[i\_vec\_\_]";

1959  o\_ << " = vals\_r\_\_[pos\_\_++];" << EOL;

1960  for (size\_t dim = 0; dim < dims.size(); ++dim) {

1961  generate\_indent(dims.size() + 2 - dim,o\_);

1962  o\_ << "}" << EOL;

1963  }

1964  o\_ << INDENT2 << "}" << EOL;

1965  }

1966  // same as simplex

1967  void operator()(unit\_vector\_var\_decl const& x) const {

1968  std::vector<expression> dims = x.dims\_;

1969  var\_size\_validator\_(x);

1970  var\_resizer\_(x);

1971  o\_ << INDENT2 << "vals\_r\_\_ = context\_\_.vals\_r(\"" << x.name\_ << "\");" << EOL;

1972  o\_ << INDENT2 << "pos\_\_ = 0;" << EOL;

1973  o\_ << INDENT2 << "size\_t " << x.name\_ << "\_i\_vec\_lim\_\_ = ";

1974  generate\_expression(x.K\_,o\_);

1975  o\_ << ";" << EOL;

1976  o\_ << INDENT2 << "for (size\_t " << "i\_vec\_\_ = 0; " << "i\_vec\_\_ < " << x.name\_ << "\_i\_vec\_lim\_\_; ++i\_vec\_\_) {" << EOL;

1977  size\_t indentation = 2;

1978  for (size\_t dim\_up = 0U; dim\_up < dims.size(); ++dim\_up) {

1979  size\_t dim = dims.size() - dim\_up - 1U;

1980  ++indentation;

1981  generate\_indent(indentation,o\_);

1982  o\_ << "size\_t " << x.name\_ << "\_limit\_" << dim << "\_\_ = ";

1983  generate\_expression(dims[dim],o\_);

1984  o\_ << ";" << EOL;

1985  generate\_indent(indentation,o\_);

1986  o\_ << "for (size\_t i\_" << dim << "\_\_ = 0; i\_" << dim << "\_\_ < " << x.name\_ << "\_limit\_" << dim << "\_\_; ++i\_" << dim << "\_\_) {" << EOL;

1987  }

1988  generate\_indent(indentation+1,o\_);

1989  o\_ << x.name\_;

1990  for (size\_t dim = 0; dim < dims.size(); ++dim)

1991  o\_ << "[i\_" << dim << "\_\_]";

1992  o\_ << "[i\_vec\_\_]";

1993  o\_ << " = vals\_r\_\_[pos\_\_++];" << EOL;

1994  for (size\_t dim = 0; dim < dims.size(); ++dim) {

1995  generate\_indent(dims.size() + 2 - dim,o\_);

1996  o\_ << "}" << EOL;

1997  }

1998  o\_ << INDENT2 << "}" << EOL;

1999  }

2000  // diff name of dims from vector

2001  void operator()(simplex\_var\_decl const& x) const {

2002  std::vector<expression> dims = x.dims\_;

2003  var\_size\_validator\_(x);

2004  var\_resizer\_(x);

2005  o\_ << INDENT2 << "vals\_r\_\_ = context\_\_.vals\_r(\"" << x.name\_ << "\");" << EOL;

2006  o\_ << INDENT2 << "pos\_\_ = 0;" << EOL;

2007  o\_ << INDENT2 << "size\_t " << x.name\_ << "\_i\_vec\_lim\_\_ = ";

2008  generate\_expression(x.K\_,o\_);

2009  o\_ << ";" << EOL;

2010  o\_ << INDENT2 << "for (size\_t " << "i\_vec\_\_ = 0; " << "i\_vec\_\_ < " << x.name\_ << "\_i\_vec\_lim\_\_; ++i\_vec\_\_) {" << EOL;

2011  size\_t indentation = 2;

2012  for (size\_t dim\_up = 0U; dim\_up < dims.size(); ++dim\_up) {

2013  size\_t dim = dims.size() - dim\_up - 1U;

2014  ++indentation;

2015  generate\_indent(indentation,o\_);

2016  o\_ << "size\_t " << x.name\_ << "\_limit\_" << dim << "\_\_ = ";

2017  generate\_expression(dims[dim],o\_);

2018  o\_ << ";" << EOL;

2019  generate\_indent(indentation,o\_);

2020  o\_ << "for (size\_t i\_" << dim << "\_\_ = 0; i\_" << dim << "\_\_ < " << x.name\_ << "\_limit\_" << dim << "\_\_; ++i\_" << dim << "\_\_) {" << EOL;

2021  }

2022  generate\_indent(indentation+1,o\_);

2023  o\_ << x.name\_;

2024  for (size\_t dim = 0; dim < dims.size(); ++dim)

2025  o\_ << "[i\_" << dim << "\_\_]";

2026  o\_ << "[i\_vec\_\_]";

2027  o\_ << " = vals\_r\_\_[pos\_\_++];" << EOL;

2028  for (size\_t dim = 0; dim < dims.size(); ++dim) {

2029  generate\_indent(dims.size() + 2 - dim,o\_);

2030  o\_ << "}" << EOL;

2031  }

2032  o\_ << INDENT2 << "}" << EOL;

2033  }

2034  // same as simplex

2035  void operator()(ordered\_var\_decl const& x) const {

2036  std::vector<expression> dims = x.dims\_;

2037  var\_size\_validator\_(x);

2038  var\_resizer\_(x);

2039  o\_ << INDENT2 << "vals\_r\_\_ = context\_\_.vals\_r(\"" << x.name\_ << "\");" << EOL;

2040  o\_ << INDENT2 << "pos\_\_ = 0;" << EOL;

2041  o\_ << INDENT2 << "size\_t " << x.name\_ << "\_i\_vec\_lim\_\_ = ";

2042  generate\_expression(x.K\_,o\_);

2043  o\_ << ";" << EOL;

2044  o\_ << INDENT2 << "for (size\_t " << "i\_vec\_\_ = 0; " << "i\_vec\_\_ < " << x.name\_ << "\_i\_vec\_lim\_\_; ++i\_vec\_\_) {" << EOL;

2045  size\_t indentation = 2;

2046  for (size\_t dim\_up = 0U; dim\_up < dims.size(); ++dim\_up) {

2047  size\_t dim = dims.size() - dim\_up - 1U;

2048  ++indentation;

2049  generate\_indent(indentation,o\_);

2050  o\_ << "size\_t " << x.name\_ << "\_limit\_" << dim << "\_\_ = ";

2051  generate\_expression(dims[dim],o\_);

2052  o\_ << ";" << EOL;

2053  generate\_indent(indentation,o\_);

2054  o\_ << "for (size\_t i\_" << dim << "\_\_ = 0; i\_" << dim << "\_\_ < " << x.name\_ << "\_limit\_" << dim << "\_\_; ++i\_" << dim << "\_\_) {" << EOL;

2055  }

2056  generate\_indent(indentation+1,o\_);

2057  o\_ << x.name\_;

2058  for (size\_t dim = 0; dim < dims.size(); ++dim)

2059  o\_ << "[i\_" << dim << "\_\_]";

2060  o\_ << "[i\_vec\_\_]";

2061  o\_ << " = vals\_r\_\_[pos\_\_++];" << EOL;

2062  for (size\_t dim = 0; dim < dims.size(); ++dim) {

2063  generate\_indent(dims.size() + 2 - dim,o\_);

2064  o\_ << "}" << EOL;

2065  }

2066  o\_ << INDENT2 << "}" << EOL;

2067  }

2068  // same as simplex

2069  void operator()(positive\_ordered\_var\_decl const& x) const {

2070  std::vector<expression> dims = x.dims\_;

2071  var\_size\_validator\_(x);

2072  var\_resizer\_(x);

2073  o\_ << INDENT2 << "vals\_r\_\_ = context\_\_.vals\_r(\"" << x.name\_ << "\");" << EOL;

2074  o\_ << INDENT2 << "pos\_\_ = 0;" << EOL;

2075  o\_ << INDENT2 << "size\_t " << x.name\_ << "\_i\_vec\_lim\_\_ = ";

2076  generate\_expression(x.K\_,o\_);

2077  o\_ << ";" << EOL;

2078  o\_ << INDENT2 << "for (size\_t " << "i\_vec\_\_ = 0; " << "i\_vec\_\_ < " << x.name\_ << "\_i\_vec\_lim\_\_; ++i\_vec\_\_) {" << EOL;

2079  size\_t indentation = 2;

2080  for (size\_t dim\_up = 0U; dim\_up < dims.size(); ++dim\_up) {

2081  size\_t dim = dims.size() - dim\_up - 1U;

2082  ++indentation;

2083  generate\_indent(indentation,o\_);

2084  o\_ << "size\_t " << x.name\_ << "\_limit\_" << dim << "\_\_ = ";

2085  generate\_expression(dims[dim],o\_);

2086  o\_ << ";" << EOL;

2087  generate\_indent(indentation,o\_);

2088  o\_ << "for (size\_t i\_" << dim << "\_\_ = 0; i\_" << dim << "\_\_ < " << x.name\_ << "\_limit\_" << dim << "\_\_; ++i\_" << dim << "\_\_) {" << EOL;

2089  }

2090  generate\_indent(indentation+1,o\_);

2091  o\_ << x.name\_;

2092  for (size\_t dim = 0; dim < dims.size(); ++dim)

2093  o\_ << "[i\_" << dim << "\_\_]";

2094  o\_ << "[i\_vec\_\_]";

2095  o\_ << " = vals\_r\_\_[pos\_\_++];" << EOL;

2096  for (size\_t dim = 0; dim < dims.size(); ++dim) {

2097  generate\_indent(dims.size() + 2 - dim,o\_);

2098  o\_ << "}" << EOL;

2099  }

2100  o\_ << INDENT2 << "}" << EOL;

2101  }

2102  // extra loop and different accessor vs. vector

2103  void operator()(matrix\_var\_decl const& x) const {

2104  std::vector<expression> dims = x.dims\_;

2105  var\_size\_validator\_(x);

2106  var\_resizer\_(x);

2107  o\_ << INDENT2 << "vals\_r\_\_ = context\_\_.vals\_r(\"" << x.name\_ << "\");" << EOL;

2108  o\_ << INDENT2 << "pos\_\_ = 0;" << EOL;

2109  o\_ << INDENT2 << "size\_t " << x.name\_ << "\_m\_mat\_lim\_\_ = ";

2110  generate\_expression(x.M\_,o\_);

2111  o\_ << ";" << EOL;

2112  o\_ << INDENT2 << "size\_t " << x.name\_ << "\_n\_mat\_lim\_\_ = ";

2113  generate\_expression(x.N\_,o\_);

2114  o\_ << ";" << EOL;

2115  o\_ << INDENT2 << "for (size\_t " << "n\_mat\_\_ = 0; " << "n\_mat\_\_ < " << x.name\_ << "\_n\_mat\_lim\_\_; ++n\_mat\_\_) {" << EOL;

2116  o\_ << INDENT3 << "for (size\_t " << "m\_mat\_\_ = 0; " << "m\_mat\_\_ < " << x.name\_ << "\_m\_mat\_lim\_\_; ++m\_mat\_\_) {" << EOL;

2117  size\_t indentation = 3;

2118  for (size\_t dim\_up = 0U; dim\_up < dims.size(); ++dim\_up) {

2119  size\_t dim = dims.size() - dim\_up - 1U;

2120  ++indentation;

2121  generate\_indent(indentation,o\_);

2122  o\_ << "size\_t " << x.name\_ << "\_limit\_" << dim << "\_\_ = ";

2123  generate\_expression(dims[dim],o\_);

2124  o\_ << ";" << EOL;

2125  generate\_indent(indentation,o\_);

2126  o\_ << "for (size\_t i\_" << dim << "\_\_ = 0; i\_" << dim << "\_\_ < " << x.name\_ << "\_limit\_" << dim << "\_\_; ++i\_" << dim << "\_\_) {" << EOL;

2127  }

2128  generate\_indent(indentation+1,o\_);

2129  o\_ << x.name\_;

2130  for (size\_t dim = 0; dim < dims.size(); ++dim)

2131  o\_ << "[i\_" << dim << "\_\_]";

2132  o\_ << "(m\_mat\_\_,n\_mat\_\_)";

2133  o\_ << " = vals\_r\_\_[pos\_\_++];" << EOL;

2134  for (size\_t dim = 0; dim < dims.size(); ++dim) {

2135  generate\_indent(dims.size() + 2 - dim,o\_);

2136  o\_ << "}" << EOL;

2137  }

2138  o\_ << INDENT3 << "}" << EOL;

2139  o\_ << INDENT2 << "}" << EOL;

2140  }

2141  void operator()(corr\_matrix\_var\_decl const& x) const {

2142  // FIXME: cut-and-paste of cov\_matrix, very slightly different from matrix

2143  std::vector<expression> dims = x.dims\_;

2144  var\_size\_validator\_(x);

2145  var\_resizer\_(x);

2146  o\_ << INDENT2 << "vals\_r\_\_ = context\_\_.vals\_r(\"" << x.name\_ << "\");" << EOL;

2147  o\_ << INDENT2 << "pos\_\_ = 0;" << EOL;

2148  o\_ << INDENT2 << "size\_t " << x.name\_ << "\_k\_mat\_lim\_\_ = ";

2149  generate\_expression(x.K\_,o\_);

2150  o\_ << ";" << EOL;

2151  o\_ << INDENT2 << "for (size\_t " << "n\_mat\_\_ = 0; " << "n\_mat\_\_ < " << x.name\_ << "\_k\_mat\_lim\_\_; ++n\_mat\_\_) {" << EOL;

2152  o\_ << INDENT3 << "for (size\_t " << "m\_mat\_\_ = 0; " << "m\_mat\_\_ < " << x.name\_ << "\_k\_mat\_lim\_\_; ++m\_mat\_\_) {" << EOL;

2153  size\_t indentation = 3;

2154  for (size\_t dim\_up = 0U; dim\_up < dims.size(); ++dim\_up) {

2155  size\_t dim = dims.size() - dim\_up - 1U;

2156  ++indentation;

2157  generate\_indent(indentation,o\_);

2158  o\_ << "size\_t " << x.name\_ << "\_limit\_" << dim << "\_\_ = ";

2159  generate\_expression(dims[dim],o\_);

2160  o\_ << ";" << EOL;

2161  generate\_indent(indentation,o\_);

2162  o\_ << "for (size\_t i\_" << dim << "\_\_ = 0; i\_" << dim << "\_\_ < " << x.name\_ << "\_limit\_" << dim << "\_\_; ++i\_" << dim << "\_\_) {" << EOL;

2163  }

2164  generate\_indent(indentation+1,o\_);

2165  o\_ << x.name\_;

2166  for (size\_t dim = 0; dim < dims.size(); ++dim)

2167  o\_ << "[i\_" << dim << "\_\_]";

2168  o\_ << "(m\_mat\_\_,n\_mat\_\_)";

2169  o\_ << " = vals\_r\_\_[pos\_\_++];" << EOL;

2170  for (size\_t dim = 0; dim < dims.size(); ++dim) {

2171  generate\_indent(dims.size() + 2 - dim,o\_);

2172  o\_ << "}" << EOL;

2173  }

2174  o\_ << INDENT3 << "}" << EOL;

2175  o\_ << INDENT2 << "}" << EOL;

2176  }

2177  void operator()(cholesky\_factor\_var\_decl const& x) const {

2178  // FIXME: cut and paste of cov\_matrix

2179  std::vector<expression> dims = x.dims\_;

2180  var\_size\_validator\_(x);

2181  var\_resizer\_(x);

2182  o\_ << INDENT2 << "vals\_r\_\_ = context\_\_.vals\_r(\"" << x.name\_ << "\");" << EOL;

2183  o\_ << INDENT2 << "pos\_\_ = 0;" << EOL;

2184

2185  o\_ << INDENT2 << "size\_t " << x.name\_ << "\_m\_mat\_lim\_\_ = ";

2186  generate\_expression(x.M\_,o\_);

2187  o\_ << ";" << EOL;

2188

2189  o\_ << INDENT2 << "size\_t " << x.name\_ << "\_n\_mat\_lim\_\_ = ";

2190  generate\_expression(x.N\_,o\_);

2191  o\_ << ";" << EOL;

2192

2193  o\_ << INDENT2 << "for (size\_t " << "n\_mat\_\_ = 0; " << "n\_mat\_\_ < " << x.name\_ << "\_n\_mat\_lim\_\_; ++n\_mat\_\_) {" << EOL;

2194  o\_ << INDENT3 << "for (size\_t " << "m\_mat\_\_ = 0; " << "m\_mat\_\_ < " << x.name\_ << "\_m\_mat\_lim\_\_; ++m\_mat\_\_) {" << EOL;

2195

2196  size\_t indentation = 3;

2197  for (size\_t dim\_up = 0U; dim\_up < dims.size(); ++dim\_up) {

2198  size\_t dim = dims.size() - dim\_up - 1U;

2199  ++indentation;

2200  generate\_indent(indentation,o\_);

2201  o\_ << "size\_t " << x.name\_ << "\_limit\_" << dim << "\_\_ = ";

2202  generate\_expression(dims[dim],o\_);

2203  o\_ << ";" << EOL;

2204  generate\_indent(indentation,o\_);

2205  o\_ << "for (size\_t i\_" << dim << "\_\_ = 0; i\_" << dim << "\_\_ < " << x.name\_ << "\_limit\_" << dim << "\_\_; ++i\_" << dim << "\_\_) {"

2206  << EOL;

2207  }

2208  generate\_indent(indentation+1,o\_);

2209  o\_ << x.name\_;

2210  for (size\_t dim = 0; dim < dims.size(); ++dim)

2211  o\_ << "[i\_" << dim << "\_\_]";

2212  o\_ << "(m\_mat\_\_,n\_mat\_\_)";

2213  o\_ << " = vals\_r\_\_[pos\_\_++];" << EOL;

2214  for (size\_t dim = 0; dim < dims.size(); ++dim) {

2215  generate\_indent(dims.size() + 2 - dim,o\_);

2216  o\_ << "}" << EOL;

2217  }

2218

2219  o\_ << INDENT3 << "}" << EOL;

2220  o\_ << INDENT2 << "}" << EOL;

2221  }

2222  void operator()(cholesky\_corr\_var\_decl const& x) const {

2223  // FIXME: cut and paste of cholesky\_factor\_var\_decl

2224  std::vector<expression> dims = x.dims\_;

2225  var\_size\_validator\_(x);

2226  var\_resizer\_(x);

2227  o\_ << INDENT2 << "vals\_r\_\_ = context\_\_.vals\_r(\"" << x.name\_ << "\");" << EOL;

2228  o\_ << INDENT2 << "pos\_\_ = 0;" << EOL;

2229

2230  o\_ << INDENT2 << "size\_t " << x.name\_ << "\_m\_mat\_lim\_\_ = ";

2231  generate\_expression(x.K\_,o\_);

2232  o\_ << ";" << EOL;

2233

2234  o\_ << INDENT2 << "size\_t " << x.name\_ << "\_n\_mat\_lim\_\_ = ";

2235  generate\_expression(x.K\_,o\_);

2236  o\_ << ";" << EOL;

2237

2238  o\_ << INDENT2 << "for (size\_t " << "n\_mat\_\_ = 0; " << "n\_mat\_\_ < " << x.name\_ << "\_n\_mat\_lim\_\_; ++n\_mat\_\_) {" << EOL;

2239  o\_ << INDENT3 << "for (size\_t " << "m\_mat\_\_ = 0; " << "m\_mat\_\_ < " << x.name\_ << "\_m\_mat\_lim\_\_; ++m\_mat\_\_) {" << EOL;

2240

2241  size\_t indentation = 3;

2242  for (size\_t dim\_up = 0U; dim\_up < dims.size(); ++dim\_up) {

2243  size\_t dim = dims.size() - dim\_up - 1U;

2244  ++indentation;

2245  generate\_indent(indentation,o\_);

2246  o\_ << "size\_t " << x.name\_ << "\_limit\_" << dim << "\_\_ = ";

2247  generate\_expression(dims[dim],o\_);

2248  o\_ << ";" << EOL;

2249  generate\_indent(indentation,o\_);

2250  o\_ << "for (size\_t i\_" << dim << "\_\_ = 0; i\_" << dim << "\_\_ < " << x.name\_ << "\_limit\_" << dim << "\_\_; ++i\_" << dim << "\_\_) {"

2251  << EOL;

2252  }

2253  generate\_indent(indentation+1,o\_);

2254  o\_ << x.name\_;

2255  for (size\_t dim = 0; dim < dims.size(); ++dim)

2256  o\_ << "[i\_" << dim << "\_\_]";

2257  o\_ << "(m\_mat\_\_,n\_mat\_\_)";

2258  o\_ << " = vals\_r\_\_[pos\_\_++];" << EOL;

2259  for (size\_t dim = 0; dim < dims.size(); ++dim) {

2260  generate\_indent(dims.size() + 2 - dim,o\_);

2261  o\_ << "}" << EOL;

2262  }

2263

2264  o\_ << INDENT3 << "}" << EOL;

2265  o\_ << INDENT2 << "}" << EOL;

2266  }

2267  void operator()(cov\_matrix\_var\_decl const& x) const {

2268  std::vector<expression> dims = x.dims\_;

2269  var\_size\_validator\_(x);

2270  var\_resizer\_(x);

2271  o\_ << INDENT2 << "vals\_r\_\_ = context\_\_.vals\_r(\"" << x.name\_ << "\");" << EOL;

2272  o\_ << INDENT2 << "pos\_\_ = 0;" << EOL;

2273  o\_ << INDENT2 << "size\_t " << x.name\_ << "\_k\_mat\_lim\_\_ = ";

2274  generate\_expression(x.K\_,o\_);

2275  o\_ << ";" << EOL;

2276  o\_ << INDENT2 << "for (size\_t " << "n\_mat\_\_ = 0; " << "n\_mat\_\_ < " << x.name\_ << "\_k\_mat\_lim\_\_; ++n\_mat\_\_) {" << EOL;

2277  o\_ << INDENT3 << "for (size\_t " << "m\_mat\_\_ = 0; " << "m\_mat\_\_ < " << x.name\_ << "\_k\_mat\_lim\_\_; ++m\_mat\_\_) {" << EOL;

2278  size\_t indentation = 3;

2279  for (size\_t dim\_up = 0U; dim\_up < dims.size(); ++dim\_up) {

2280  size\_t dim = dims.size() - dim\_up - 1U;

2281  ++indentation;

2282  generate\_indent(indentation,o\_);

2283  o\_ << "size\_t " << x.name\_ << "\_limit\_" << dim << "\_\_ = ";

2284  generate\_expression(dims[dim],o\_);

2285  o\_ << ";" << EOL;

2286  generate\_indent(indentation,o\_);

2287  o\_ << "for (size\_t i\_" << dim << "\_\_ = 0; i\_" << dim << "\_\_ < " << x.name\_ << "\_limit\_" << dim << "\_\_; ++i\_" << dim << "\_\_) {" << EOL;

2288  }

2289  generate\_indent(indentation+1,o\_);

2290  o\_ << x.name\_;

2291  for (size\_t dim = 0; dim < dims.size(); ++dim)

2292  o\_ << "[i\_" << dim << "\_\_]";

2293  o\_ << "(m\_mat\_\_,n\_mat\_\_)";

2294  o\_ << " = vals\_r\_\_[pos\_\_++];" << EOL;

2295  for (size\_t dim = 0; dim < dims.size(); ++dim) {

2296  generate\_indent(dims.size() + 2 - dim,o\_);

2297  o\_ << "}" << EOL;

2298  }

2299  o\_ << INDENT3 << "}" << EOL;

2300  o\_ << INDENT2 << "}" << EOL;

2301  }

2302  };

2303

2304  void suppress\_warning(const std::string& indent,

2305  const std::string& var\_name,

2306  std::ostream& o) {

2307  o << indent << "(void) "

2308  << var\_name << ";"

2309  << " // dummy call to supress warning"

2310  << EOL;

2311  }

2312

2313  void generate\_member\_var\_inits(const std::vector<var\_decl>& vs,

2314  std::ostream& o) {

2315  dump\_member\_var\_visgen vis(o);

2316  for (size\_t i = 0; i < vs.size(); ++i)

2317  boost::apply\_visitor(vis, vs[i].decl\_);

2318  }

2319

2320  void generate\_destructor(const std::string& model\_name,

2321  std::ostream& o) {

2322  o << EOL

2323  << INDENT << "~" << model\_name << "() { }"

2324  << EOL2;

2325  }

2326

2327  // know all data is set and range expressions only depend on data

2328  struct set\_param\_ranges\_visgen : public visgen {

2329  set\_param\_ranges\_visgen(std::ostream& o)

2330  : visgen(o) {

2331  }

2332  void operator()(const nil& /\*x\*/) const { }

2333  void operator()(const int\_var\_decl& x) const {

2334  generate\_increment\_i(x.dims\_);

2335  // for loop for ranges

2336  for (size\_t i = 0; i < x.dims\_.size(); ++i) {

2337  generate\_indent(i + 2, o\_);

2338  o\_ << "for (size\_t i\_" << i << "\_\_ = 0; ";

2339  o\_ << "i\_" << i << "\_\_ < ";

2340  generate\_expression(x.dims\_[i],o\_);

2341  o\_ << "; ++i\_" << i << "\_\_) {" << EOL;

2342  }

2343  // add range

2344  generate\_indent(x.dims\_.size() + 2,o\_);

2345  o\_ << "param\_ranges\_i\_\_.push\_back(std::pair<int,int>(";

2346  generate\_expression(x.range\_.low\_,o\_);

2347  o\_ << ", ";

2348  generate\_expression(x.range\_.high\_,o\_);

2349  o\_ << "));" << EOL;

2350  // close for loop

2351  for (size\_t i = 0; i < x.dims\_.size(); ++i) {

2352  generate\_indent(x.dims\_.size() + 1 - i, o\_);

2353  o\_ << "}" << EOL;

2354  }

2355  }

2356  void operator()(const double\_var\_decl& x) const {

2357  generate\_increment(x.dims\_);

2358  }

2359  void operator()(const vector\_var\_decl& x) const {

2360  generate\_increment(x.M\_,x.dims\_);

2361  }

2362  void operator()(const row\_vector\_var\_decl& x) const {

2363  generate\_increment(x.N\_,x.dims\_);

2364  }

2365  void operator()(const matrix\_var\_decl& x) const {

2366  generate\_increment(x.M\_,x.N\_,x.dims\_);

2367  }

2368  void operator()(const unit\_vector\_var\_decl& x) const {

2369  // only K-1 vals

2370  o\_ << INDENT2 << "num\_params\_r\_\_ += (";

2371  generate\_expression(x.K\_,o\_);

2372  o\_ << " - 1)";

2373  for (size\_t i = 0; i < x.dims\_.size(); ++i) {

2374  o\_ << " \* ";

2375  generate\_expression(x.dims\_[i],o\_);

2376  }

2377  o\_ << ";" << EOL;

2378  }

2379  void operator()(const simplex\_var\_decl& x) const {

2380  // only K-1 vals

2381  o\_ << INDENT2 << "num\_params\_r\_\_ += (";

2382  generate\_expression(x.K\_,o\_);

2383  o\_ << " - 1)";

2384  for (size\_t i = 0; i < x.dims\_.size(); ++i) {

2385  o\_ << " \* ";

2386  generate\_expression(x.dims\_[i],o\_);

2387  }

2388  o\_ << ";" << EOL;

2389  }

2390  void operator()(const ordered\_var\_decl& x) const {

2391  generate\_increment(x.K\_,x.dims\_);

2392  }

2393  void operator()(const positive\_ordered\_var\_decl& x) const {

2394  generate\_increment(x.K\_,x.dims\_);

2395  }

2396  void operator()(const cholesky\_factor\_var\_decl& x) const {

2397  o\_ << INDENT2 << "num\_params\_r\_\_ += ((";

2398  // N \* (N + 1) / 2 + (M - N) \* M

2399  generate\_expression(x.N\_,o\_);

2400  o\_ << " \* (";

2401  generate\_expression(x.N\_,o\_);

2402  o\_ << " + 1)) / 2 + (";

2403  generate\_expression(x.M\_,o\_);

2404  o\_ << " - ";

2405  generate\_expression(x.N\_,o\_);

2406  o\_ << ") \* ";

2407  generate\_expression(x.N\_,o\_);

2408  o\_ << ")";

2409  for (size\_t i = 0; i < x.dims\_.size(); ++i) {

2410  o\_ << " \* ";

2411  generate\_expression(x.dims\_[i],o\_);

2412  }

2413  o\_ << ";" << EOL;

2414  }

2415  void operator()(const cholesky\_corr\_var\_decl& x) const {

2416  // FIXME: cut and paste ofcorr\_matrix\_var\_decl

2417  o\_ << INDENT2 << "num\_params\_r\_\_ += ((";

2418  generate\_expression(x.K\_,o\_);

2419  o\_ << " \* (";

2420  generate\_expression(x.K\_,o\_);

2421  o\_ << " - 1)) / 2)";

2422  for (size\_t i = 0; i < x.dims\_.size(); ++i) {

2423  o\_ << " \* ";

2424  generate\_expression(x.dims\_[i],o\_);

2425  }

2426  o\_ << ";" << EOL;

2427  }

2428  void operator()(const cov\_matrix\_var\_decl& x) const {

2429  // (K \* (K - 1))/2 + K ?? define fun(K) = ??

2430  o\_ << INDENT2 << "num\_params\_r\_\_ += ((";

2431  generate\_expression(x.K\_,o\_);

2432  o\_ << " \* (";

2433  generate\_expression(x.K\_,o\_);

2434  o\_ << " - 1)) / 2 + ";

2435  generate\_expression(x.K\_,o\_);

2436  o\_ << ")";

2437  for (size\_t i = 0; i < x.dims\_.size(); ++i) {

2438  o\_ << " \* ";

2439  generate\_expression(x.dims\_[i],o\_);

2440  }

2441  o\_ << ";" << EOL;

2442  }

2443  void operator()(const corr\_matrix\_var\_decl& x) const {

2444  o\_ << INDENT2 << "num\_params\_r\_\_ += ((";

2445  generate\_expression(x.K\_,o\_);

2446  o\_ << " \* (";

2447  generate\_expression(x.K\_,o\_);

2448  o\_ << " - 1)) / 2)";

2449  for (size\_t i = 0; i < x.dims\_.size(); ++i) {

2450  o\_ << " \* ";

2451  generate\_expression(x.dims\_[i],o\_);

2452  }

2453  o\_ << ";" << EOL;

2454  }

2455  // cut-and-paste from next for r

2456  void generate\_increment\_i(std::vector<expression> dims) const {

2457  if (dims.size() == 0) {

2458  o\_ << INDENT2 << "++num\_params\_i\_\_;" << EOL;

2459  return;

2460  }

2461  o\_ << INDENT2 << "num\_params\_r\_\_ += ";

2462  for (size\_t i = 0; i < dims.size(); ++i) {

2463  if (i > 0) o\_ << " \* ";

2464  generate\_expression(dims[i],o\_);

2465  }

2466  o\_ << ";" << EOL;

2467  }

2468  void generate\_increment(std::vector<expression> dims) const {

2469  if (dims.size() == 0) {

2470  o\_ << INDENT2 << "++num\_params\_r\_\_;" << EOL;

2471  return;

2472  }

2473  o\_ << INDENT2 << "num\_params\_r\_\_ += ";

2474  for (size\_t i = 0; i < dims.size(); ++i) {

2475  if (i > 0) o\_ << " \* ";

2476  generate\_expression(dims[i],o\_);

2477  }

2478  o\_ << ";" << EOL;

2479  }

2480  void generate\_increment(expression K,

2481  std::vector<expression> dims) const {

2482  o\_ << INDENT2 << "num\_params\_r\_\_ += ";

2483  generate\_expression(K,o\_);

2484  for (size\_t i = 0; i < dims.size(); ++i) {

2485  o\_ << " \* ";

2486  generate\_expression(dims[i],o\_);

2487  }

2488  o\_ << ";" << EOL;

2489

2490  }

2491  void generate\_increment(expression M, expression N,

2492  std::vector<expression> dims) const {

2493  o\_ << INDENT2 << "num\_params\_r\_\_ += ";

2494  generate\_expression(M,o\_);

2495  o\_ << " \* ";

2496  generate\_expression(N,o\_);

2497  for (size\_t i = 0; i < dims.size(); ++i) {

2498  o\_ << " \* ";

2499  generate\_expression(dims[i],o\_);

2500  }

2501  o\_ << ";" << EOL;

2502  }

2503  };

2504

2505  void generate\_set\_param\_ranges(const std::vector<var\_decl>& var\_decls,

2506  std::ostream& o) {

2507  // o << EOL;

2508  // o << INDENT << "void set\_param\_ranges() {" << EOL;

2509  o << INDENT2 << "num\_params\_r\_\_ = 0U;" << EOL;

2510  o << INDENT2 << "param\_ranges\_i\_\_.clear();" << EOL;

2511  set\_param\_ranges\_visgen vis(o);

2512  for (size\_t i = 0; i < var\_decls.size(); ++i)

2513  boost::apply\_visitor(vis,var\_decls[i].decl\_);

2514  // o << INDENT << "}" << EOL;

2515  }

2516

2517  void generate\_constructor(const program& prog,

2518  const std::string& model\_name,

2519  std::ostream& o) {

2520  o << INDENT << model\_name << "(stan::io::var\_context& context\_\_," << EOL;

2521  o << INDENT << " std::ostream\* pstream\_\_ = 0)"

2522  << EOL;

2523  o << INDENT2 << ": prob\_grad(0) {"

2524  << EOL; // resize 0 with var\_resizing

2525  o << INDENT2 << "static const char\* function\_\_ = \""

2526  << model\_name << "\_namespace::" << model\_name << "(%1%)\";" << EOL;

2527  suppress\_warning(INDENT2, "function\_\_", o);

2528  o << INDENT2 << "size\_t pos\_\_;" << EOL;

2529  suppress\_warning(INDENT2, "pos\_\_", o);

2530  o << INDENT2 << "std::vector<int> vals\_i\_\_;" << EOL;

2531  o << INDENT2 << "std::vector<double> vals\_r\_\_;" << EOL;

2532

2533  generate\_member\_var\_inits(prog.data\_decl\_,o);

2534

2535  o << EOL;

2536  generate\_comment("validate data",2,o);

2537  generate\_validate\_var\_decls(prog.data\_decl\_,2,o);

2538

2539  generate\_var\_resizing(prog.derived\_data\_decl\_.first, o);

2540  o << EOL;

2541  bool include\_sampling = false;

2542  bool is\_var = false;

2543  bool is\_fun\_return = false;

2544  for (size\_t i = 0; i < prog.derived\_data\_decl\_.second.size(); ++i)

2545  generate\_statement(prog.derived\_data\_decl\_.second[i],

2546  2,o,include\_sampling,is\_var,is\_fun\_return);

2547

2548  o << EOL;

2549  generate\_comment("validate transformed data",2,o);

2550  generate\_validate\_var\_decls(prog.derived\_data\_decl\_.first,2,o);

2551

2552  o << EOL;

2553  generate\_comment("set parameter ranges",2,o);

2554  generate\_set\_param\_ranges(prog.parameter\_decl\_,o);

2555  // o << EOL << INDENT2 << "set\_param\_ranges();" << EOL;

2556

2557  o << INDENT << "}" << EOL;

2558  }

2559

2560  struct generate\_init\_visgen : public visgen {

2561  var\_size\_validating\_visgen var\_size\_validator\_;

2562  generate\_init\_visgen(std::ostream& o)

2563  : visgen(o),

2564  var\_size\_validator\_(o,"initialization") {

2565  }

2566  void operator()(nil const& /\*x\*/) const { } // dummy

2567  void operator()(int\_var\_decl const& x) const {

2568  generate\_check\_int(x.name\_,x.dims\_.size());

2569  var\_size\_validator\_(x);

2570  generate\_declaration(x.name\_,"int",x.dims\_);

2571  generate\_buffer\_loop("i",x.name\_, x.dims\_);

2572  generate\_write\_loop("integer(",x.name\_,x.dims\_);

2573  }

2574  template <typename D>

2575  std::string function\_args(const std::string& fun\_prefix,

2576  const D& x) const {

2577  std::stringstream ss;

2578  ss << fun\_prefix;

2579  if (has\_lub(x)) {

2580  ss << "\_lub\_unconstrain(";

2581  generate\_expression(x.range\_.low\_.expr\_,ss);

2582  ss << ',';

2583  generate\_expression(x.range\_.high\_.expr\_,ss);

2584  ss << ',';

2585  } else if (has\_lb(x)) {

2586  ss << "\_lb\_unconstrain(";

2587  generate\_expression(x.range\_.low\_.expr\_,ss);

2588  ss << ',';

2589  } else if (has\_ub(x)) {

2590  ss << "\_ub\_unconstrain(";

2591  generate\_expression(x.range\_.high\_.expr\_,ss);

2592  ss << ',';

2593  } else {

2594  ss << "\_unconstrain(";

2595  }

2596  return ss.str();

2597  }

2598

2599  void operator()(double\_var\_decl const& x) const {

2600  generate\_check\_double(x.name\_,x.dims\_.size());

2601  var\_size\_validator\_(x);

2602  generate\_declaration(x.name\_,"double",x.dims\_);

2603  generate\_buffer\_loop("r",x.name\_,x.dims\_);

2604  generate\_write\_loop(function\_args("scalar",x),

2605  x.name\_, x.dims\_);

2606  }

2607  void operator()(vector\_var\_decl const& x) const {

2608  generate\_check\_double(x.name\_,x.dims\_.size() + 1);

2609  var\_size\_validator\_(x);

2610  generate\_declaration(x.name\_,"vector\_d",x.dims\_,x.M\_);

2611  generate\_buffer\_loop("r",x.name\_,x.dims\_,x.M\_);

2612  generate\_write\_loop(function\_args("vector",x),

2613  x.name\_,x.dims\_);

2614  }

2615  void operator()(row\_vector\_var\_decl const& x) const {

2616  generate\_check\_double(x.name\_,x.dims\_.size() + 1);

2617  var\_size\_validator\_(x);

2618  generate\_declaration(x.name\_,"row\_vector\_d",x.dims\_,x.N\_);

2619  generate\_buffer\_loop("r",x.name\_,x.dims\_,x.N\_);

2620  generate\_write\_loop(function\_args("row\_vector",x),

2621  x.name\_,x.dims\_);

2622  }

2623  void operator()(matrix\_var\_decl const& x) const {

2624  generate\_check\_double(x.name\_,x.dims\_.size() + 2);

2625  var\_size\_validator\_(x);

2626  generate\_declaration(x.name\_,"matrix\_d",x.dims\_,x.M\_,x.N\_);

2627  generate\_buffer\_loop("r",x.name\_,x.dims\_,x.M\_,x.N\_);

2628  generate\_write\_loop(function\_args("matrix",x),

2629  x.name\_,x.dims\_);

2630  }

2631  void operator()(unit\_vector\_var\_decl const& x) const {

2632  generate\_check\_double(x.name\_,x.dims\_.size() + 1);

2633  var\_size\_validator\_(x);

2634  generate\_declaration(x.name\_,"vector\_d",x.dims\_,x.K\_);

2635  generate\_buffer\_loop("r",x.name\_,x.dims\_,x.K\_);

2636  generate\_write\_loop("unit\_vector\_unconstrain(",x.name\_,x.dims\_);

2637  }

2638  void operator()(simplex\_var\_decl const& x) const {

2639  generate\_check\_double(x.name\_,x.dims\_.size() + 1);

2640  var\_size\_validator\_(x);

2641  generate\_declaration(x.name\_,"vector\_d",x.dims\_,x.K\_);

2642  generate\_buffer\_loop("r",x.name\_,x.dims\_,x.K\_);

2643  generate\_write\_loop("simplex\_unconstrain(",x.name\_,x.dims\_);

2644  }

2645  void operator()(ordered\_var\_decl const& x) const {

2646  generate\_check\_double(x.name\_,x.dims\_.size() + 1);

2647  var\_size\_validator\_(x);

2648  generate\_declaration(x.name\_,"vector\_d",x.dims\_,x.K\_);

2649  generate\_buffer\_loop("r",x.name\_,x.dims\_,x.K\_);

2650  generate\_write\_loop("ordered\_unconstrain(",x.name\_,x.dims\_);

2651  }

2652  void operator()(positive\_ordered\_var\_decl const& x) const {

2653  generate\_check\_double(x.name\_,x.dims\_.size() + 1);

2654  var\_size\_validator\_(x);

2655  generate\_declaration(x.name\_,"vector\_d",x.dims\_,x.K\_);

2656  generate\_buffer\_loop("r",x.name\_,x.dims\_,x.K\_);

2657  generate\_write\_loop("positive\_ordered\_unconstrain(",x.name\_,x.dims\_);

2658  }

2659  void operator()(cholesky\_factor\_var\_decl const& x) const {

2660  generate\_check\_double(x.name\_,x.dims\_.size() + 2);

2661  var\_size\_validator\_(x);

2662  generate\_declaration(x.name\_,"matrix\_d",x.dims\_,x.M\_,x.N\_);

2663  generate\_buffer\_loop("r",x.name\_,x.dims\_,x.M\_,x.N\_);

2664  generate\_write\_loop("cholesky\_factor\_unconstrain(",x.name\_,x.dims\_);

2665  }

2666  void operator()(cholesky\_corr\_var\_decl const& x) const {

2667  generate\_check\_double(x.name\_,x.dims\_.size() + 2);

2668  var\_size\_validator\_(x);

2669  generate\_declaration(x.name\_,"matrix\_d",x.dims\_,x.K\_,x.K\_);

2670  generate\_buffer\_loop("r",x.name\_,x.dims\_,x.K\_,x.K\_);

2671  generate\_write\_loop("cholesky\_corr\_unconstrain(",x.name\_,x.dims\_);

2672  }

2673  void operator()(cov\_matrix\_var\_decl const& x) const {

2674  generate\_check\_double(x.name\_,x.dims\_.size() + 2);

2675  var\_size\_validator\_(x);

2676  generate\_declaration(x.name\_,"matrix\_d",x.dims\_,x.K\_,x.K\_);

2677  generate\_buffer\_loop("r",x.name\_,x.dims\_,x.K\_,x.K\_);

2678  generate\_write\_loop("cov\_matrix\_unconstrain(",x.name\_,x.dims\_);

2679  }

2680  void operator()(corr\_matrix\_var\_decl const& x) const {

2681  generate\_check\_double(x.name\_,x.dims\_.size() + 2);

2682  var\_size\_validator\_(x);

2683  generate\_declaration(x.name\_,"matrix\_d",x.dims\_,x.K\_,x.K\_);

2684  generate\_buffer\_loop("r",x.name\_,x.dims\_,x.K\_,x.K\_);

2685  generate\_write\_loop("corr\_matrix\_unconstrain(",x.name\_,x.dims\_);

2686  }

2687  void generate\_write\_loop(const std::string& write\_method\_name,

2688  const std::string& var\_name,

2689  const std::vector<expression>& dims) const {

2690  generate\_dims\_loop\_fwd(dims);

2691  o\_ << "try { writer\_\_." << write\_method\_name;

2692  generate\_name\_dims(var\_name,dims.size());

2693  o\_ << "); } catch (const std::exception& e) { "

2694  " throw std::runtime\_error(std::string(\"Error transforming variable "

2695  << var\_name << ": \") + e.what()); }" << EOL;

2696  }

2697  void generate\_name\_dims(const std::string name,

2698  size\_t num\_dims) const {

2699  o\_ << name;

2700  for (size\_t i = 0; i < num\_dims; ++i)

2701  o\_ << "[i" << i << "\_\_]";

2702  }

2703  void generate\_declaration(const std::string& name,

2704  const std::string& base\_type,

2705  const std::vector<expression>& dims,

2706  const expression& type\_arg1 = expression(),

2707  const expression& type\_arg2 = expression()) const {

2708  o\_ << INDENT2;

2709  generate\_type(base\_type,dims,dims.size(),o\_);

2710  o\_ << ' ' << name;

2711

2712  generate\_initializer(o\_,base\_type,dims,type\_arg1,type\_arg2);

2713  }

2714  void generate\_indent\_num\_dims(size\_t base\_indent,

2715  const std::vector<expression>& dims,

2716  const expression& dim1,

2717  const expression& dim2) const {

2718  generate\_indent(dims.size() + base\_indent,o\_);

2719  if (!is\_nil(dim1)) o\_ << INDENT;

2720  if (!is\_nil(dim2)) o\_ << INDENT;

2721  }

2722  void generate\_buffer\_loop(const std::string& base\_type,

2723  const std::string& name,

2724  const std::vector<expression>& dims,

2725  const expression& dim1 = expression(),

2726  const expression& dim2 = expression(),

2727  int indent = 2U) const {

2728  size\_t size = dims.size();

2729  bool is\_matrix = !is\_nil(dim1) && !is\_nil(dim2);

2730  bool is\_vector = !is\_nil(dim1) && is\_nil(dim2);

2731  int extra\_indent = is\_matrix ? 2U : is\_vector ? 1U : 0U;

2732  if (is\_matrix) {

2733  generate\_indent(indent,o\_);

2734  o\_ << "for (int j2\_\_ = 0U; j2\_\_ < ";

2735  generate\_expression(dim2.expr\_,o\_);

2736  o\_ << "; ++j2\_\_)" << EOL;

2737

2738  generate\_indent(indent+1,o\_);

2739  o\_ << "for (int j1\_\_ = 0U; j1\_\_ < ";

2740  generate\_expression(dim1.expr\_,o\_);

2741  o\_ << "; ++j1\_\_)" << EOL;

2742  } else if (is\_vector) {

2743  generate\_indent(indent,o\_);

2744  o\_ << "for (int j1\_\_ = 0U; j1\_\_ < ";

2745  generate\_expression(dim1.expr\_,o\_);

2746  o\_ << "; ++j1\_\_)" << EOL;

2747  }

2748  for (size\_t i = 0; i < size; ++i) {

2749  size\_t idx = size - i - 1;

2750  generate\_indent(i + indent + extra\_indent, o\_);

2751  o\_ << "for (int i" << idx << "\_\_ = 0U; i" << idx << "\_\_ < ";

2752  generate\_expression(dims[idx].expr\_,o\_);

2753  o\_ << "; ++i" << idx << "\_\_)" << EOL;

2754  }

2755  generate\_indent\_num\_dims(2U,dims,dim1,dim2);

2756  o\_ << name;

2757  for (size\_t i = 0; i < dims.size(); ++i)

2758  o\_ << "[i" << i << "\_\_]";

2759  if (is\_matrix)

2760  o\_ << "(j1\_\_,j2\_\_)";

2761  else if (is\_vector)

2762  o\_ << "(j1\_\_)";

2763  o\_ << " = vals\_" << base\_type << "\_\_[pos\_\_++];" << EOL;

2764  }

2765  void generate\_dims\_loop\_fwd(const std::vector<expression>& dims,

2766  int indent = 2U) const {

2767  size\_t size = dims.size();

2768  for (size\_t i = 0; i < size; ++i) {

2769  generate\_indent(i + indent, o\_);

2770  o\_ << "for (int i" << i << "\_\_ = 0U; i" << i << "\_\_ < ";

2771  generate\_expression(dims[i].expr\_,o\_);

2772  o\_ << "; ++i" << i << "\_\_)" << EOL;

2773  }

2774  generate\_indent(2U + dims.size(),o\_);

2775  }

2776  void generate\_check\_int(const std::string& name, size\_t /\*n\*/) const {

2777  o\_ << EOL << INDENT2

2778  << "if (!(context\_\_.contains\_i(\"" << name << "\")))"

2779  << EOL << INDENT3

2780  << "throw std::runtime\_error(\"variable " << name << " missing\");" << EOL;

2781  o\_ << INDENT2 << "vals\_i\_\_ = context\_\_.vals\_i(\"" << name << "\");" << EOL;

2782  o\_ << INDENT2 << "pos\_\_ = 0U;" << EOL;

2783  }

2784  void generate\_check\_double(const std::string& name, size\_t /\*n\*/) const {

2785  o\_ << EOL << INDENT2

2786  << "if (!(context\_\_.contains\_r(\"" << name << "\")))"

2787  << EOL << INDENT3

2788  << "throw std::runtime\_error(\"variable " << name << " missing\");" << EOL;

2789  o\_ << INDENT2 << "vals\_r\_\_ = context\_\_.vals\_r(\"" << name << "\");" << EOL;

2790  o\_ << INDENT2 << "pos\_\_ = 0U;" << EOL;

2791  }

2792  };

2793

2794

2795  void generate\_init\_method(const std::vector<var\_decl>& vs,

2796  std::ostream& o) {

2797  o << EOL;

2798  o << INDENT << "void transform\_inits(const stan::io::var\_context& context\_\_," << EOL;

2799  o << INDENT << " std::vector<int>& params\_i\_\_," << EOL;

2800  o << INDENT << " std::vector<double>& params\_r\_\_) const {" << EOL;

2801  o << INDENT2 << "stan::io::writer<double> writer\_\_(params\_r\_\_,params\_i\_\_);" << EOL;

2802  o << INDENT2 << "size\_t pos\_\_;" << EOL;

2803  o << INDENT2 << "(void) pos\_\_; // dummy call to supress warning" << EOL;

2804  o << INDENT2 << "std::vector<double> vals\_r\_\_;" << EOL;

2805  o << INDENT2 << "std::vector<int> vals\_i\_\_;" << EOL;

2806  o << EOL;

2807  generate\_init\_visgen vis(o);

2808  for (size\_t i = 0; i < vs.size(); ++i)

2809  boost::apply\_visitor(vis, vs[i].decl\_);

2810

2811  o << INDENT2 << "params\_r\_\_ = writer\_\_.data\_r();" << EOL;

2812  o << INDENT2 << "params\_i\_\_ = writer\_\_.data\_i();" << EOL;

2813  o << INDENT << "}" << EOL2;

2814

2815  o << INDENT << "void transform\_inits(const stan::io::var\_context& context," << EOL;

2816  o << INDENT << " Eigen::Matrix<double,Eigen::Dynamic,1>& params\_r) const {" << EOL;

2817  o << INDENT << " std::vector<double> params\_r\_vec;" << EOL;

2818  o << INDENT << " std::vector<int> params\_i\_vec;" << EOL;

2819  o << INDENT << " transform\_inits(context, params\_i\_vec, params\_r\_vec);" << EOL;

2820  o << INDENT << " params\_r.resize(params\_r\_vec.size());" << EOL;

2821  o << INDENT << " for (int i = 0; i < params\_r.size(); ++i)" << EOL;

2822  o << INDENT << " params\_r(i) = params\_r\_vec[i];" << EOL;

2823  o << INDENT << "}" << EOL2;

2824  }

2825

2826  // see write\_csv\_visgen for similar structure

2827  struct write\_dims\_visgen : public visgen {

2828  write\_dims\_visgen(std::ostream& o)

2829  : visgen(o) {

2830  }

2831  void operator()(const nil& /\*x\*/) const { }

2832  void operator()(const int\_var\_decl& x) const {

2833  generate\_dims\_array(EMPTY\_EXP\_VECTOR,x.dims\_);

2834  }

2835  void operator()(const double\_var\_decl& x) const {

2836  generate\_dims\_array(EMPTY\_EXP\_VECTOR,x.dims\_);

2837  }

2838  void operator()(const vector\_var\_decl& x) const {

2839  std::vector<expression> matrix\_args;

2840  matrix\_args.push\_back(x.M\_);

2841  generate\_dims\_array(matrix\_args,x.dims\_);

2842  }

2843  void operator()(const row\_vector\_var\_decl& x) const {

2844  std::vector<expression> matrix\_args;

2845  matrix\_args.push\_back(x.N\_);

2846  generate\_dims\_array(matrix\_args,x.dims\_);

2847  }

2848  void operator()(const matrix\_var\_decl& x) const {

2849  std::vector<expression> matrix\_args;

2850  matrix\_args.push\_back(x.M\_);

2851  matrix\_args.push\_back(x.N\_);

2852  generate\_dims\_array(matrix\_args,x.dims\_);

2853  }

2854  void operator()(const unit\_vector\_var\_decl& x) const {

2855  std::vector<expression> matrix\_args;

2856  matrix\_args.push\_back(x.K\_);

2857  generate\_dims\_array(matrix\_args,x.dims\_);

2858  }

2859  void operator()(const simplex\_var\_decl& x) const {

2860  std::vector<expression> matrix\_args;

2861  matrix\_args.push\_back(x.K\_);

2862  generate\_dims\_array(matrix\_args,x.dims\_);

2863  }

2864  void operator()(const ordered\_var\_decl& x) const {

2865  std::vector<expression> matrix\_args;

2866  matrix\_args.push\_back(x.K\_);

2867  generate\_dims\_array(matrix\_args,x.dims\_);

2868  }

2869  void operator()(const positive\_ordered\_var\_decl& x) const {

2870  std::vector<expression> matrix\_args;

2871  matrix\_args.push\_back(x.K\_);

2872  generate\_dims\_array(matrix\_args,x.dims\_);

2873  }

2874  void operator()(const cholesky\_factor\_var\_decl& x) const {

2875  std::vector<expression> matrix\_args;

2876  matrix\_args.push\_back(x.M\_);

2877  matrix\_args.push\_back(x.N\_);

2878  generate\_dims\_array(matrix\_args,x.dims\_);

2879  }

2880  void operator()(const cholesky\_corr\_var\_decl& x) const {

2881  std::vector<expression> matrix\_args;

2882  matrix\_args.push\_back(x.K\_);

2883  matrix\_args.push\_back(x.K\_);

2884  generate\_dims\_array(matrix\_args,x.dims\_);

2885  }

2886  void operator()(const cov\_matrix\_var\_decl& x) const {

2887  std::vector<expression> matrix\_args;

2888  matrix\_args.push\_back(x.K\_);

2889  matrix\_args.push\_back(x.K\_);

2890  generate\_dims\_array(matrix\_args,x.dims\_);

2891  }

2892  void operator()(const corr\_matrix\_var\_decl& x) const {

2893  std::vector<expression> matrix\_args;

2894  matrix\_args.push\_back(x.K\_);

2895  matrix\_args.push\_back(x.K\_);

2896  generate\_dims\_array(matrix\_args,x.dims\_);

2897  }

2898  void

2899  generate\_dims\_array(const std::vector<expression>& matrix\_dims\_exprs,

2900  const std::vector<expression>& array\_dims\_exprs)

2901  const {

2902

2903  o\_ << INDENT2 << "dims\_\_.resize(0);" << EOL;

2904  for (size\_t i = 0; i < array\_dims\_exprs.size(); ++i) {

2905  o\_ << INDENT2 << "dims\_\_.push\_back(";

2906  generate\_expression(array\_dims\_exprs[i].expr\_, o\_);

2907  o\_ << ");" << EOL;

2908  }

2909  // cut and paste above with matrix\_dims\_exprs

2910  for (size\_t i = 0; i < matrix\_dims\_exprs.size(); ++i) {

2911  o\_ << INDENT2 << "dims\_\_.push\_back(";

2912  generate\_expression(matrix\_dims\_exprs[i].expr\_, o\_);

2913  o\_ << ");" << EOL;

2914  }

2915  o\_ << INDENT2 << "dimss\_\_.push\_back(dims\_\_);" << EOL;

2916  }

2917

2918  };

2919

2920  void generate\_dims\_method(const program& prog,

2921  std::ostream& o) {

2922  write\_dims\_visgen vis(o);

2923  o << EOL << INDENT

2924  << "void get\_dims(std::vector<std::vector<size\_t> >& dimss\_\_) const {"

2925  << EOL;

2926

2927  o << INDENT2 << "dimss\_\_.resize(0);" << EOL;

2928  o << INDENT2 << "std::vector<size\_t> dims\_\_;" << EOL;

2929

2930  // parameters

2931  for (size\_t i = 0; i < prog.parameter\_decl\_.size(); ++i) {

2932  boost::apply\_visitor(vis,prog.parameter\_decl\_[i].decl\_);

2933  }

2934  // transformed parameters

2935  for (size\_t i = 0; i < prog.derived\_decl\_.first.size(); ++i) {

2936  boost::apply\_visitor(vis,prog.derived\_decl\_.first[i].decl\_);

2937  }

2938  // generated quantities

2939  for (size\_t i = 0; i < prog.generated\_decl\_.first.size(); ++i) {

2940  boost::apply\_visitor(vis,prog.generated\_decl\_.first[i].decl\_);

2941  }

2942  o << INDENT << "}" << EOL2;

2943  }

2944

2945

2946

2947  // see write\_csv\_visgen for similar structure

2948  struct write\_param\_names\_visgen : public visgen {

2949  write\_param\_names\_visgen(std::ostream& o)

2950  : visgen(o) {

2951  }

2952  void operator()(const nil& /\*x\*/) const { }

2953  void operator()(const int\_var\_decl& x) const {

2954  generate\_param\_names(x.name\_);

2955  }

2956  void operator()(const double\_var\_decl& x) const {

2957  generate\_param\_names(x.name\_);

2958  }

2959  void operator()(const vector\_var\_decl& x) const {

2960  generate\_param\_names(x.name\_);

2961  }

2962  void operator()(const row\_vector\_var\_decl& x) const {

2963  generate\_param\_names(x.name\_);

2964  }

2965  void operator()(const matrix\_var\_decl& x) const {

2966  generate\_param\_names(x.name\_);

2967  }

2968  void operator()(const unit\_vector\_var\_decl& x) const {

2969  generate\_param\_names(x.name\_);

2970  }

2971  void operator()(const simplex\_var\_decl& x) const {

2972  generate\_param\_names(x.name\_);

2973  }

2974  void operator()(const ordered\_var\_decl& x) const {

2975  generate\_param\_names(x.name\_);

2976  }

2977  void operator()(const positive\_ordered\_var\_decl& x) const {

2978  generate\_param\_names(x.name\_);

2979  }

2980  void operator()(const cholesky\_factor\_var\_decl& x) const {

2981  generate\_param\_names(x.name\_);

2982  }

2983  void operator()(const cholesky\_corr\_var\_decl& x) const {

2984  generate\_param\_names(x.name\_);

2985  }

2986  void operator()(const cov\_matrix\_var\_decl& x) const {

2987  generate\_param\_names(x.name\_);

2988  }

2989  void operator()(const corr\_matrix\_var\_decl& x) const {

2990  generate\_param\_names(x.name\_);

2991  }

2992  void

2993  generate\_param\_names(const std::string& name) const {

2994  o\_ << INDENT2

2995  << "names\_\_.push\_back(\"" << name << "\");"

2996  << EOL;

2997  }

2998  };

2999

3000

3001  void generate\_param\_names\_method(const program& prog,

3002  std::ostream& o) {

3003  write\_param\_names\_visgen vis(o);

3004  o << EOL << INDENT

3005  << "void get\_param\_names(std::vector<std::string>& names\_\_) const {"

3006  << EOL;

3007

3008  o << INDENT2

3009  << "names\_\_.resize(0);"

3010  << EOL;

3011

3012  // parameters

3013  for (size\_t i = 0; i < prog.parameter\_decl\_.size(); ++i) {

3014  boost::apply\_visitor(vis,prog.parameter\_decl\_[i].decl\_);

3015  }

3016  // transformed parameters

3017  for (size\_t i = 0; i < prog.derived\_decl\_.first.size(); ++i) {

3018  boost::apply\_visitor(vis,prog.derived\_decl\_.first[i].decl\_);

3019  }

3020  // generated quantities

3021  for (size\_t i = 0; i < prog.generated\_decl\_.first.size(); ++i) {

3022  boost::apply\_visitor(vis,prog.generated\_decl\_.first[i].decl\_);

3023  }

3024

3025  o << INDENT << "}" << EOL2;

3026  }

3027

3028

3029

3030  // see write\_csv\_visgen for similar structure

3031  struct write\_csv\_header\_visgen : public visgen {

3032  write\_csv\_header\_visgen(std::ostream& o)

3033  : visgen(o) {

3034  }

3035  void operator()(const nil& /\*x\*/) const { }

3036  void operator()(const int\_var\_decl& x) const {

3037  generate\_csv\_header\_array(EMPTY\_EXP\_VECTOR,x.name\_,x.dims\_);

3038  }

3039  void operator()(const double\_var\_decl& x) const {

3040  generate\_csv\_header\_array(EMPTY\_EXP\_VECTOR,x.name\_,x.dims\_);

3041  }

3042  void operator()(const vector\_var\_decl& x) const {

3043  std::vector<expression> matrix\_args;

3044  matrix\_args.push\_back(x.M\_);

3045  generate\_csv\_header\_array(matrix\_args,x.name\_,x.dims\_);

3046  }

3047  void operator()(const row\_vector\_var\_decl& x) const {

3048  std::vector<expression> matrix\_args;

3049  matrix\_args.push\_back(x.N\_);

3050  generate\_csv\_header\_array(matrix\_args,x.name\_,x.dims\_);

3051  }

3052  void operator()(const matrix\_var\_decl& x) const {

3053  std::vector<expression> matrix\_args;

3054  matrix\_args.push\_back(x.M\_);

3055  matrix\_args.push\_back(x.N\_);

3056  generate\_csv\_header\_array(matrix\_args,x.name\_,x.dims\_);

3057  }

3058  void operator()(const unit\_vector\_var\_decl& x) const {

3059  std::vector<expression> matrix\_args;

3060  matrix\_args.push\_back(x.K\_);

3061  generate\_csv\_header\_array(matrix\_args,x.name\_,x.dims\_);

3062  }

3063  void operator()(const simplex\_var\_decl& x) const {

3064  std::vector<expression> matrix\_args;

3065  matrix\_args.push\_back(x.K\_);

3066  generate\_csv\_header\_array(matrix\_args,x.name\_,x.dims\_);

3067  }

3068  void operator()(const ordered\_var\_decl& x) const {

3069  std::vector<expression> matrix\_args;

3070  matrix\_args.push\_back(x.K\_);

3071  generate\_csv\_header\_array(matrix\_args,x.name\_,x.dims\_);

3072  }

3073  void operator()(const positive\_ordered\_var\_decl& x) const {

3074  std::vector<expression> matrix\_args;

3075  matrix\_args.push\_back(x.K\_);

3076  generate\_csv\_header\_array(matrix\_args,x.name\_,x.dims\_);

3077  }

3078  void operator()(const cholesky\_factor\_var\_decl& x) const {

3079  std::vector<expression> matrix\_args;

3080  matrix\_args.push\_back(x.M\_);

3081  matrix\_args.push\_back(x.N\_);

3082  generate\_csv\_header\_array(matrix\_args,x.name\_,x.dims\_);

3083  }

3084  void operator()(const cholesky\_corr\_var\_decl& x) const {

3085  std::vector<expression> matrix\_args;

3086  matrix\_args.push\_back(x.K\_);

3087  matrix\_args.push\_back(x.K\_);

3088  generate\_csv\_header\_array(matrix\_args,x.name\_,x.dims\_);

3089  }

3090  void operator()(const cov\_matrix\_var\_decl& x) const {

3091  std::vector<expression> matrix\_args;

3092  matrix\_args.push\_back(x.K\_);

3093  matrix\_args.push\_back(x.K\_);

3094  generate\_csv\_header\_array(matrix\_args,x.name\_,x.dims\_);

3095  }

3096  void operator()(const corr\_matrix\_var\_decl& x) const {

3097  std::vector<expression> matrix\_args;

3098  matrix\_args.push\_back(x.K\_);

3099  matrix\_args.push\_back(x.K\_);

3100  generate\_csv\_header\_array(matrix\_args,x.name\_,x.dims\_);

3101  }

3102  void

3103  generate\_csv\_header\_array(const std::vector<expression>& matrix\_dims,

3104  const std::string& name,

3105  const std::vector<expression>& dims) const {

3106

3107  // begin for loop dims

3108  std::vector<expression> combo\_dims(dims);

3109  for (size\_t i = 0; i < matrix\_dims.size(); ++i)

3110  combo\_dims.push\_back(matrix\_dims[i]);

3111

3112  for (size\_t i = combo\_dims.size(); i-- > 0; ) {

3113  generate\_indent(1 + combo\_dims.size() - i,o\_);

3114  o\_ << "for (int k\_" << i << "\_\_ = 1;"

3115  << " k\_" << i << "\_\_ <= ";

3116  generate\_expression(combo\_dims[i].expr\_,o\_);

3117  o\_ << "; ++k\_" << i << "\_\_) {" << EOL; // begin (1)

3118  }

3119

3120  // variable + indices

3121  generate\_indent(2 + combo\_dims.size(),o\_);

3122  o\_ << "writer\_\_.comma();" << EOL; // only writes comma after first call

3123

3124  generate\_indent(2 + combo\_dims.size(),o\_);

3125  o\_ << "o\_\_ << \"" << name << '"';

3126  for (size\_t i = 0; i < combo\_dims.size(); ++i)

3127  o\_ << " << '.' << k\_" << i << "\_\_";

3128  o\_ << ';' << EOL;

3129

3130  // end for loop dims

3131  for (size\_t i = 0; i < combo\_dims.size(); ++i) {

3132  generate\_indent(1 + combo\_dims.size() - i,o\_);

3133  o\_ << "}" << EOL; // end (1)

3134  }

3135  }

3136  // void

3137  // generate\_csv\_header\_array(const std::vector<expression>& matrix\_dims,

3138  // const std::string& name,

3139  // const std::vector<expression>& dims) const {

3140

3141  // // begin for loop dims

3142  // std::vector<expression> combo\_dims(dims);

3143  // for (size\_t i = 0; i < matrix\_dims.size(); ++i)

3144  // combo\_dims.push\_back(matrix\_dims[i]);

3145

3146  // for (size\_t i = 0; i < combo\_dims.size(); ++i) {

3147  // generate\_indent(2 + i,o\_);

3148  // o\_ << "for (int k\_" << i << "\_\_ = 1;"

3149  // << " k\_" << i << "\_\_ <= ";

3150  // generate\_expression(combo\_dims[i].expr\_,o\_);

3151  // o\_ << "; ++k\_" << i << "\_\_) {" << EOL; // begin (1)

3152  // }

3153

3154  // // variable + indices

3155  // generate\_indent(2 + combo\_dims.size(),o\_);

3156  // o\_ << "writer\_\_.comma();" << EOL; // only writes comma after first call

3157

3158  // generate\_indent(2 + combo\_dims.size(),o\_);

3159  // o\_ << "o\_\_ << \"" << name << '"';

3160  // for (size\_t i = 0; i < combo\_dims.size(); ++i)

3161  // o\_ << " << '.' << k\_" << i << "\_\_";

3162  // o\_ << ';' << EOL;

3163

3164  // // end for loop dims

3165  // for (size\_t i = 0; i < combo\_dims.size(); ++i) {

3166  // generate\_indent(1 + combo\_dims.size() - i,o\_);

3167  // o\_ << "}" << EOL; // end (1)

3168  // }

3169  // }

3170  };

3171

3172

3173  void generate\_write\_csv\_header\_method(const program& prog,

3174  std::ostream& o) {

3175  write\_csv\_header\_visgen vis(o);

3176  o << EOL << INDENT << "void write\_csv\_header(std::ostream& o\_\_) const {" << EOL;

3177  o << INDENT2 << "stan::io::csv\_writer writer\_\_(o\_\_);" << EOL;

3178

3179  // parameters

3180  for (size\_t i = 0; i < prog.parameter\_decl\_.size(); ++i) {

3181  boost::apply\_visitor(vis,prog.parameter\_decl\_[i].decl\_);

3182  }

3183  // transformed parameters

3184  for (size\_t i = 0; i < prog.derived\_decl\_.first.size(); ++i) {

3185  boost::apply\_visitor(vis,prog.derived\_decl\_.first[i].decl\_);

3186  }

3187  // generated quantities

3188  for (size\_t i = 0; i < prog.generated\_decl\_.first.size(); ++i) {

3189  boost::apply\_visitor(vis,prog.generated\_decl\_.first[i].decl\_);

3190  }

3191  o << INDENT2 << "writer\_\_.newline();" << EOL;

3192  o << INDENT << "}" << EOL2;

3193  }

3194

3195  // see write\_csv\_visgen for similar structure

3196  struct constrained\_param\_names\_visgen : public visgen {

3197  constrained\_param\_names\_visgen(std::ostream& o)

3198  : visgen(o) {

3199  }

3200  void operator()(const nil& /\*x\*/) const { }

3201  void operator()(const int\_var\_decl& x) const {

3202  generate\_param\_names\_array(EMPTY\_EXP\_VECTOR,x.name\_,x.dims\_);

3203  }

3204  void operator()(const double\_var\_decl& x) const {

3205  generate\_param\_names\_array(EMPTY\_EXP\_VECTOR,x.name\_,x.dims\_);

3206  }

3207  void operator()(const vector\_var\_decl& x) const {

3208  std::vector<expression> matrix\_args;

3209  matrix\_args.push\_back(x.M\_);

3210  generate\_param\_names\_array(matrix\_args,x.name\_,x.dims\_);

3211  }

3212  void operator()(const row\_vector\_var\_decl& x) const {

3213  std::vector<expression> matrix\_args;

3214  matrix\_args.push\_back(x.N\_);

3215  generate\_param\_names\_array(matrix\_args,x.name\_,x.dims\_);

3216  }

3217  void operator()(const matrix\_var\_decl& x) const {

3218  std::vector<expression> matrix\_args;

3219  matrix\_args.push\_back(x.M\_);

3220  matrix\_args.push\_back(x.N\_);

3221  generate\_param\_names\_array(matrix\_args,x.name\_,x.dims\_);

3222  }

3223  void operator()(const unit\_vector\_var\_decl& x) const {

3224  std::vector<expression> matrix\_args;

3225  matrix\_args.push\_back(x.K\_);

3226  generate\_param\_names\_array(matrix\_args,x.name\_,x.dims\_);

3227  }

3228  void operator()(const simplex\_var\_decl& x) const {

3229  std::vector<expression> matrix\_args;

3230  matrix\_args.push\_back(x.K\_);

3231  generate\_param\_names\_array(matrix\_args,x.name\_,x.dims\_);

3232  }

3233  void operator()(const ordered\_var\_decl& x) const {

3234  std::vector<expression> matrix\_args;

3235  matrix\_args.push\_back(x.K\_);

3236  generate\_param\_names\_array(matrix\_args,x.name\_,x.dims\_);

3237  }

3238  void operator()(const positive\_ordered\_var\_decl& x) const {

3239  std::vector<expression> matrix\_args;

3240  matrix\_args.push\_back(x.K\_);

3241  generate\_param\_names\_array(matrix\_args,x.name\_,x.dims\_);

3242  }

3243  void operator()(const cholesky\_factor\_var\_decl& x) const {

3244  std::vector<expression> matrix\_args;

3245  matrix\_args.push\_back(x.M\_);

3246  matrix\_args.push\_back(x.N\_);

3247  generate\_param\_names\_array(matrix\_args,x.name\_,x.dims\_);

3248  }

3249  void operator()(const cholesky\_corr\_var\_decl& x) const {

3250  std::vector<expression> matrix\_args;

3251  matrix\_args.push\_back(x.K\_);

3252  matrix\_args.push\_back(x.K\_);

3253  generate\_param\_names\_array(matrix\_args,x.name\_,x.dims\_);

3254  }

3255  void operator()(const cov\_matrix\_var\_decl& x) const {

3256  std::vector<expression> matrix\_args;

3257  matrix\_args.push\_back(x.K\_);

3258  matrix\_args.push\_back(x.K\_);

3259  generate\_param\_names\_array(matrix\_args,x.name\_,x.dims\_);

3260  }

3261  void operator()(const corr\_matrix\_var\_decl& x) const {

3262  std::vector<expression> matrix\_args;

3263  matrix\_args.push\_back(x.K\_);

3264  matrix\_args.push\_back(x.K\_);

3265  generate\_param\_names\_array(matrix\_args,x.name\_,x.dims\_);

3266  }

3267  void

3268  generate\_param\_names\_array(const std::vector<expression>& matrix\_dims,

3269  const std::string& name,

3270  const std::vector<expression>& dims) const {

3271

3272  // begin for loop dims

3273  std::vector<expression> combo\_dims(dims);

3274  for (size\_t i = 0; i < matrix\_dims.size(); ++i)

3275  combo\_dims.push\_back(matrix\_dims[i]);

3276

3277  for (size\_t i = combo\_dims.size(); i-- > 0; ) {

3278  generate\_indent(1 + combo\_dims.size() - i,o\_);

3279  o\_ << "for (int k\_" << i << "\_\_ = 1;"

3280  << " k\_" << i << "\_\_ <= ";

3281  generate\_expression(combo\_dims[i].expr\_,o\_);

3282  o\_ << "; ++k\_" << i << "\_\_) {" << EOL; // begin (1)

3283  }

3284

3285  generate\_indent(2 + combo\_dims.size(),o\_);

3286  o\_ << "param\_name\_stream\_\_.str(std::string());" << EOL;

3287

3288  generate\_indent(2 + combo\_dims.size(),o\_);

3289  o\_ << "param\_name\_stream\_\_ << \"" << name << '"';

3290

3291  for (size\_t i = 0; i < combo\_dims.size(); ++i)

3292  o\_ << " << '.' << k\_" << i << "\_\_";

3293  o\_ << ';' << EOL;

3294

3295  generate\_indent(2 + combo\_dims.size(),o\_);

3296  o\_ << "param\_names\_\_.push\_back(param\_name\_stream\_\_.str());" << EOL;

3297

3298  // end for loop dims

3299  for (size\_t i = 0; i < combo\_dims.size(); ++i) {

3300  generate\_indent(1 + combo\_dims.size() - i,o\_);

3301  o\_ << "}" << EOL; // end (1)

3302  }

3303  }

3304

3305  };

3306

3307

3308  void generate\_constrained\_param\_names\_method(const program& prog,

3309  std::ostream& o) {

3310  o << EOL << INDENT

3311  << "void constrained\_param\_names(std::vector<std::string>& param\_names\_\_,"

3312  << EOL << INDENT

3313  << " bool include\_tparams\_\_ = true,"

3314  << EOL << INDENT

3315  << " bool include\_gqs\_\_ = true) const {"

3316  << EOL << INDENT2

3317  << "std::stringstream param\_name\_stream\_\_;" << EOL;

3318

3319  constrained\_param\_names\_visgen vis(o);

3320  // parameters

3321  for (size\_t i = 0; i < prog.parameter\_decl\_.size(); ++i) {

3322  boost::apply\_visitor(vis,prog.parameter\_decl\_[i].decl\_);

3323  }

3324

3325  o << EOL << INDENT2

3326  << "if (!include\_gqs\_\_ && !include\_tparams\_\_) return;"

3327  << EOL;

3328

3329  // transformed parameters

3330  for (size\_t i = 0; i < prog.derived\_decl\_.first.size(); ++i) {

3331  boost::apply\_visitor(vis,prog.derived\_decl\_.first[i].decl\_);

3332  }

3333

3334  o << EOL << INDENT2

3335  << "if (!include\_gqs\_\_) return;"

3336  << EOL;

3337

3338  // generated quantities

3339  for (size\_t i = 0; i < prog.generated\_decl\_.first.size(); ++i) {

3340  boost::apply\_visitor(vis,prog.generated\_decl\_.first[i].decl\_);

3341  }

3342

3343  o << INDENT << "}" << EOL2;

3344  }

3345

3346  // see write\_csv\_visgen for similar structure

3347  struct unconstrained\_param\_names\_visgen : public visgen {

3348  unconstrained\_param\_names\_visgen(std::ostream& o)

3349  : visgen(o) {

3350  }

3351  void operator()(const nil& /\*x\*/) const { }

3352  void operator()(const int\_var\_decl& x) const {

3353  generate\_param\_names\_array(EMPTY\_EXP\_VECTOR,x.name\_,x.dims\_);

3354  }

3355  void operator()(const double\_var\_decl& x) const {

3356  generate\_param\_names\_array(EMPTY\_EXP\_VECTOR,x.name\_,x.dims\_);

3357  }

3358  void operator()(const vector\_var\_decl& x) const {

3359  std::vector<expression> matrix\_args;

3360  matrix\_args.push\_back(x.M\_);

3361  generate\_param\_names\_array(matrix\_args,x.name\_,x.dims\_);

3362  }

3363  void operator()(const row\_vector\_var\_decl& x) const {

3364  std::vector<expression> matrix\_args;

3365  matrix\_args.push\_back(x.N\_);

3366  generate\_param\_names\_array(matrix\_args,x.name\_,x.dims\_);

3367  }

3368  void operator()(const matrix\_var\_decl& x) const {

3369  std::vector<expression> matrix\_args;

3370  matrix\_args.push\_back(x.M\_);

3371  matrix\_args.push\_back(x.N\_);

3372  generate\_param\_names\_array(matrix\_args,x.name\_,x.dims\_);

3373  }

3374  void operator()(const unit\_vector\_var\_decl& x) const {

3375  std::vector<expression> matrix\_args;

3376  matrix\_args.push\_back(binary\_op(x.K\_,"-",int\_literal(1)));

3377  generate\_param\_names\_array(matrix\_args,x.name\_,x.dims\_);

3378  }

3379  void operator()(const simplex\_var\_decl& x) const {

3380  std::vector<expression> matrix\_args;

3381  matrix\_args.push\_back(binary\_op(x.K\_,"-",int\_literal(1)));

3382  generate\_param\_names\_array(matrix\_args,x.name\_,x.dims\_);

3383  }

3384  void operator()(const ordered\_var\_decl& x) const {

3385  std::vector<expression> matrix\_args;

3386  matrix\_args.push\_back(x.K\_);

3387  generate\_param\_names\_array(matrix\_args,x.name\_,x.dims\_);

3388  }

3389  void operator()(const positive\_ordered\_var\_decl& x) const {

3390  std::vector<expression> matrix\_args;

3391  matrix\_args.push\_back(x.K\_);

3392  generate\_param\_names\_array(matrix\_args,x.name\_,x.dims\_);

3393  }

3394  void operator()(const cholesky\_factor\_var\_decl& x) const {

3395  // FIXME: cut-and-paste of cov\_matrix

3396  std::vector<expression> matrix\_args;

3397  // (N \* (N + 1)) / 2 + (M - N) \* N

3398  matrix\_args.push\_back(binary\_op(binary\_op(binary\_op(x.N\_,

3399  "\*",

3400  binary\_op(x.N\_,

3401  "+",

3402  int\_literal(1))),

3403  "/",

3404  int\_literal(2)),

3405  "+",

3406  binary\_op(binary\_op(x.M\_,

3407  "-",

3408  x.N\_),

3409  "\*",

3410  x.N\_)));

3411  generate\_param\_names\_array(matrix\_args,x.name\_,x.dims\_);

3412  }

3413  void operator()(const cholesky\_corr\_var\_decl& x) const {

3414  // FIXME: cut-and-paste of corr\_matrix

3415  std::vector<expression> matrix\_args;

3416  // (K \* (K - 1)) / 2

3417  matrix\_args.push\_back(binary\_op(binary\_op(x.K\_,

3418  "\*",

3419  binary\_op(x.K\_,

3420  "-",

3421  int\_literal(1))),

3422  "/",

3423  int\_literal(2)));

3424  generate\_param\_names\_array(matrix\_args,x.name\_,x.dims\_);

3425  }

3426  void operator()(const cov\_matrix\_var\_decl& x) const {

3427  std::vector<expression> matrix\_args;

3428  matrix\_args.push\_back(binary\_op(x.K\_,

3429  "+",

3430  binary\_op(binary\_op(x.K\_,

3431  "\*",

3432  binary\_op(x.K\_,

3433  "-",

3434  int\_literal(1))),

3435  "/",

3436  int\_literal(2))));

3437  generate\_param\_names\_array(matrix\_args,x.name\_,x.dims\_);

3438  }

3439  void operator()(const corr\_matrix\_var\_decl& x) const {

3440  std::vector<expression> matrix\_args;

3441  matrix\_args.push\_back(binary\_op(binary\_op(x.K\_,

3442  "\*",

3443  binary\_op(x.K\_,

3444  "-",

3445  int\_literal(1))),

3446  "/",

3447  int\_literal(2)));

3448  generate\_param\_names\_array(matrix\_args,x.name\_,x.dims\_);

3449  }

3450  // FIXME: sharing instead of cut-and-paste from constrained

3451  void

3452  generate\_param\_names\_array(const std::vector<expression>& matrix\_dims,

3453  const std::string& name,

3454  const std::vector<expression>& dims) const {

3455

3456  // begin for loop dims

3457  std::vector<expression> combo\_dims(dims);

3458  for (size\_t i = 0; i < matrix\_dims.size(); ++i)

3459  combo\_dims.push\_back(matrix\_dims[i]);

3460

3461  for (size\_t i = combo\_dims.size(); i-- > 0; ) {

3462  generate\_indent(1 + combo\_dims.size() - i,o\_);

3463  o\_ << "for (int k\_" << i << "\_\_ = 1;"

3464  << " k\_" << i << "\_\_ <= ";

3465  generate\_expression(combo\_dims[i].expr\_,o\_);

3466  o\_ << "; ++k\_" << i << "\_\_) {" << EOL; // begin (1)

3467  }

3468

3469  generate\_indent(2 + combo\_dims.size(),o\_);

3470  o\_ << "param\_name\_stream\_\_.str(std::string());" << EOL;

3471

3472  generate\_indent(2 + combo\_dims.size(),o\_);

3473  o\_ << "param\_name\_stream\_\_ << \"" << name << '"';

3474

3475  for (size\_t i = 0; i < combo\_dims.size(); ++i)

3476  o\_ << " << '.' << k\_" << i << "\_\_";

3477  o\_ << ';' << EOL;

3478

3479  generate\_indent(2 + combo\_dims.size(),o\_);

3480  o\_ << "param\_names\_\_.push\_back(param\_name\_stream\_\_.str());" << EOL;

3481

3482  // end for loop dims

3483  for (size\_t i = 0; i < combo\_dims.size(); ++i) {

3484  generate\_indent(1 + combo\_dims.size() - i,o\_);

3485  o\_ << "}" << EOL; // end (1)

3486  }

3487  }

3488  };

3489

3490

3491  void generate\_unconstrained\_param\_names\_method(const program& prog,

3492  std::ostream& o) {

3493  o << EOL << INDENT

3494  << "void unconstrained\_param\_names(std::vector<std::string>& param\_names\_\_,"

3495  << EOL << INDENT

3496  << " bool include\_tparams\_\_ = true,"

3497  << EOL << INDENT

3498  << " bool include\_gqs\_\_ = true) const {"

3499  << EOL << INDENT2

3500  << "std::stringstream param\_name\_stream\_\_;" << EOL;

3501

3502  unconstrained\_param\_names\_visgen vis(o);

3503  // parameters

3504  for (size\_t i = 0; i < prog.parameter\_decl\_.size(); ++i) {

3505  boost::apply\_visitor(vis,prog.parameter\_decl\_[i].decl\_);

3506  }

3507

3508  o << EOL << INDENT2

3509  << "if (!include\_gqs\_\_ && !include\_tparams\_\_) return;"

3510  << EOL;

3511

3512  // transformed parameters

3513  for (size\_t i = 0; i < prog.derived\_decl\_.first.size(); ++i) {

3514  boost::apply\_visitor(vis,prog.derived\_decl\_.first[i].decl\_);

3515  }

3516

3517  o << EOL << INDENT2

3518  << "if (!include\_gqs\_\_) return;"

3519  << EOL;

3520

3521  // generated quantities

3522  for (size\_t i = 0; i < prog.generated\_decl\_.first.size(); ++i) {

3523  boost::apply\_visitor(vis,prog.generated\_decl\_.first[i].decl\_);

3524  }

3525

3526  o << INDENT << "}" << EOL2;

3527  }

3528

3529

3530  // see init\_member\_var\_visgen for cut & paste

3531  struct write\_csv\_visgen : public visgen {

3532  write\_csv\_visgen(std::ostream& o)

3533  : visgen(o) {

3534  }

3535  template <typename D>

3536  void generate\_initialize\_array\_bounded(const D& x, const std::string& base\_type,

3537  const std::string& read\_fun\_prefix,

3538  const std::vector<expression>& dim\_args) const {

3539  std::vector<expression> read\_args;

3540  std::string read\_fun(read\_fun\_prefix);

3541  if (has\_lub(x)) {

3542  read\_fun += "\_lub";

3543  read\_args.push\_back(x.range\_.low\_);

3544  read\_args.push\_back(x.range\_.high\_);

3545  } else if (has\_lb(x)) {

3546  read\_fun += "\_lb";

3547  read\_args.push\_back(x.range\_.low\_);

3548  } else if (has\_ub(x)) {

3549  read\_fun += "\_ub";

3550  read\_args.push\_back(x.range\_.high\_);

3551  }

3552  for (size\_t i = 0; i < dim\_args.size(); ++i)

3553  read\_args.push\_back(dim\_args[i]);

3554  generate\_initialize\_array(base\_type,read\_fun,read\_args,x.name\_,x.dims\_);

3555  }

3556  void operator()(const nil& /\*x\*/) const { }

3557  void operator()(const int\_var\_decl& x) const {

3558  generate\_initialize\_array("int","integer",EMPTY\_EXP\_VECTOR,

3559  x.name\_,x.dims\_);

3560  }

3561  void operator()(const double\_var\_decl& x) const {

3562  std::vector<expression> read\_args;

3563  generate\_initialize\_array\_bounded(x,"double","scalar",read\_args);

3564  }

3565  void operator()(const vector\_var\_decl& x) const {

3566  std::vector<expression> read\_args;

3567  read\_args.push\_back(x.M\_);

3568  generate\_initialize\_array\_bounded(x,"vector\_d","vector",read\_args);

3569  }

3570  void operator()(const row\_vector\_var\_decl& x) const {

3571  std::vector<expression> read\_args;

3572  read\_args.push\_back(x.N\_);

3573  generate\_initialize\_array\_bounded(x,"row\_vector\_d","row\_vector",read\_args);

3574  }

3575  void operator()(const matrix\_var\_decl& x) const {

3576  std::vector<expression> read\_args;

3577  read\_args.push\_back(x.M\_);

3578  read\_args.push\_back(x.N\_);

3579  generate\_initialize\_array\_bounded(x,"matrix\_d","matrix",read\_args);

3580  }

3581  void operator()(const unit\_vector\_var\_decl& x) const {

3582  std::vector<expression> read\_args;

3583  read\_args.push\_back(x.K\_);

3584  generate\_initialize\_array("vector\_d","unit\_vector",read\_args,x.name\_,x.dims\_);

3585  }

3586  void operator()(const simplex\_var\_decl& x) const {

3587  std::vector<expression> read\_args;

3588  read\_args.push\_back(x.K\_);

3589  generate\_initialize\_array("vector\_d","simplex",read\_args,x.name\_,x.dims\_);

3590  }

3591  void operator()(const ordered\_var\_decl& x) const {

3592  std::vector<expression> read\_args;

3593  read\_args.push\_back(x.K\_);

3594  generate\_initialize\_array("vector\_d","ordered",read\_args,x.name\_,x.dims\_);

3595  }

3596  void operator()(const positive\_ordered\_var\_decl& x) const {

3597  std::vector<expression> read\_args;

3598  read\_args.push\_back(x.K\_);

3599  generate\_initialize\_array("vector\_d","positive\_ordered",read\_args,x.name\_,x.dims\_);

3600  }

3601  void operator()(const cholesky\_factor\_var\_decl& x) const {

3602  std::vector<expression> read\_args;

3603  read\_args.push\_back(x.M\_);

3604  read\_args.push\_back(x.N\_);

3605  generate\_initialize\_array("matrix\_d","cholesky\_factor",read\_args,x.name\_,x.dims\_);

3606  }

3607  void operator()(const cholesky\_corr\_var\_decl& x) const {

3608  std::vector<expression> read\_args;

3609  read\_args.push\_back(x.K\_);

3610  generate\_initialize\_array("matrix\_d","cholesky\_corr",read\_args,x.name\_,x.dims\_);

3611  }

3612  void operator()(const cov\_matrix\_var\_decl& x) const {

3613  std::vector<expression> read\_args;

3614  read\_args.push\_back(x.K\_);

3615  generate\_initialize\_array("matrix\_d","cov\_matrix",read\_args,x.name\_,x.dims\_);

3616  }

3617  void operator()(const corr\_matrix\_var\_decl& x) const {

3618  std::vector<expression> read\_args;

3619  read\_args.push\_back(x.K\_);

3620  generate\_initialize\_array("matrix\_d","corr\_matrix",read\_args,x.name\_,x.dims\_);

3621  }

3622  void generate\_initialize\_array(const std::string& var\_type,

3623  const std::string& read\_type,

3624  const std::vector<expression>& read\_args,

3625  const std::string& name,

3626  const std::vector<expression>& dims) const {

3627  if (dims.size() == 0) {

3628  generate\_indent(2,o\_);

3629  o\_ << var\_type << " ";

3630  o\_ << name << " = in\_\_." << read\_type << "\_constrain(";

3631  for (size\_t j = 0; j < read\_args.size(); ++j) {

3632  if (j > 0) o\_ << ",";

3633  generate\_expression(read\_args[j],o\_);

3634  }

3635  o\_ << ");" << EOL;

3636  o\_ << INDENT2 << "writer\_\_.write(" << name << ");" << EOL;

3637  return;

3638  }

3639  o\_ << INDENT2;

3640  for (size\_t i = 0; i < dims.size(); ++i) o\_ << "vector<";

3641  o\_ << var\_type;

3642  for (size\_t i = 0; i < dims.size(); ++i) o\_ << "> ";

3643  o\_ << name << ";" << EOL;

3644  std::string name\_dims(name);

3645  for (size\_t i = 0; i < dims.size(); ++i) {

3646  generate\_indent(i + 2, o\_);

3647  o\_ << "size\_t dim\_" << name << "\_" << i << "\_\_ = ";

3648  generate\_expression(dims[i],o\_);

3649  o\_ << ";" << EOL;

3650  if (i < dims.size() - 1) {

3651  generate\_indent(i + 2, o\_);

3652  o\_ << name\_dims << ".resize(dim\_" << name << "\_" << i << "\_\_);"

3653  << EOL;

3654  name\_dims.append("[k\_").append(to\_string(i)).append("\_\_]");

3655  }

3656  generate\_indent(i + 2, o\_);

3657  o\_ << "for (size\_t k\_" << i << "\_\_ = 0;"

3658  << " k\_" << i << "\_\_ < dim\_" << name << "\_" << i << "\_\_;"

3659  << " ++k\_" << i << "\_\_) {" << EOL;

3660  if (i == dims.size() - 1) {

3661  generate\_indent(i + 3, o\_);

3662  o\_ << name\_dims << ".push\_back(in\_\_." << read\_type << "\_constrain(";

3663  for (size\_t j = 0; j < read\_args.size(); ++j) {

3664  if (j > 0) o\_ << ",";

3665  generate\_expression(read\_args[j],o\_);

3666  }

3667  o\_ << "));" << EOL;

3668  }

3669  }

3670  generate\_indent(dims.size() + 2, o\_);

3671  o\_ << "writer\_\_.write(" << name;

3672  if (dims.size() > 0) {

3673  o\_ << '[';

3674  for (size\_t i = 0; i < dims.size(); ++i) {

3675  if (i > 0) o\_ << "][";

3676  o\_ << "k\_" << i << "\_\_";

3677  }

3678  o\_ << ']';

3679  }

3680  o\_ << ");" << EOL;

3681

3682  for (size\_t i = dims.size(); i > 0; --i) {

3683  generate\_indent(i + 1, o\_);

3684  o\_ << "}" << EOL;

3685  }

3686  }

3687  };

3688

3689

3690

3691

3692  struct write\_csv\_vars\_visgen : public visgen {

3693  write\_csv\_vars\_visgen(std::ostream& o)

3694  : visgen(o) {

3695  }

3696  void operator()(const nil& /\*x\*/) const { }

3697  // FIXME: template these out

3698  void operator()(const int\_var\_decl& x) const {

3699  write\_array(x.name\_,x.dims\_);

3700  }

3701  void operator()(const double\_var\_decl& x) const {

3702  write\_array(x.name\_,x.dims\_);

3703  }

3704  void operator()(const vector\_var\_decl& x) const {

3705  write\_array(x.name\_,x.dims\_);

3706  }

3707  void operator()(const row\_vector\_var\_decl& x) const {

3708  write\_array(x.name\_,x.dims\_);

3709  }

3710  void operator()(const matrix\_var\_decl& x) const {

3711  write\_array(x.name\_,x.dims\_);

3712  }

3713  void operator()(const unit\_vector\_var\_decl& x) const {

3714  write\_array(x.name\_,x.dims\_);

3715  }

3716  void operator()(const simplex\_var\_decl& x) const {

3717  write\_array(x.name\_,x.dims\_);

3718  }

3719  void operator()(const ordered\_var\_decl& x) const {

3720  write\_array(x.name\_,x.dims\_);

3721  }

3722  void operator()(const positive\_ordered\_var\_decl& x) const {

3723  write\_array(x.name\_,x.dims\_);

3724  }

3725  void operator()(const cholesky\_factor\_var\_decl& x) const {

3726  write\_array(x.name\_,x.dims\_);

3727  }

3728  void operator()(const cholesky\_corr\_var\_decl& x) const {

3729  write\_array(x.name\_,x.dims\_);

3730  }

3731  void operator()(const cov\_matrix\_var\_decl& x) const {

3732  write\_array(x.name\_,x.dims\_);

3733  }

3734  void operator()(const corr\_matrix\_var\_decl& x) const {

3735  write\_array(x.name\_,x.dims\_);

3736  }

3737  void write\_array(const std::string& name,

3738  const std::vector<expression>& dims) const {

3739  if (dims.size() == 0) {

3740  o\_ << INDENT2 << "writer\_\_.write(" << name << ");" << EOL;

3741  return;

3742  }

3743  for (size\_t i = 0; i < dims.size(); ++i) {

3744  generate\_indent(i + 2, o\_);

3745  o\_ << "for (int k\_" << i << "\_\_ = 0;"

3746  << " k\_" << i << "\_\_ < ";

3747  generate\_expression(dims[i],o\_);

3748  o\_ << "; ++k\_" << i << "\_\_) {" << EOL;

3749  }

3750

3751  generate\_indent(dims.size() + 2, o\_);

3752  o\_ << "writer\_\_.write(" << name;

3753  if (dims.size() > 0) {

3754  o\_ << '[';

3755  for (size\_t i = 0; i < dims.size(); ++i) {

3756  if (i > 0) o\_ << "][";

3757  o\_ << "k\_" << i << "\_\_";

3758  }

3759  o\_ << ']';

3760  }

3761  o\_ << ");" << EOL;

3762

3763  for (size\_t i = dims.size(); i > 0; --i) {

3764  generate\_indent(i + 1, o\_);

3765  o\_ << "}" << EOL;

3766  }

3767  }

3768  };

3769

3770

3771  void generate\_write\_csv\_method(const program& prog,

3772  const std::string& model\_name,

3773  std::ostream& o) {

3774  o << INDENT << "template <typename RNG>" << EOL;

3775  o << INDENT << "void write\_csv(RNG& base\_rng\_\_," << EOL;

3776  o << INDENT << " std::vector<double>& params\_r\_\_," << EOL;

3777  o << INDENT << " std::vector<int>& params\_i\_\_," << EOL;

3778  o << INDENT << " std::ostream& o\_\_," << EOL;

3779  o << INDENT << " std::ostream\* pstream\_\_ = 0) const {" << EOL;

3780  o << INDENT2 << "stan::io::reader<double> in\_\_(params\_r\_\_,params\_i\_\_);"

3781  << EOL;

3782  o << INDENT2 << "stan::io::csv\_writer writer\_\_(o\_\_);" << EOL;

3783  o << INDENT2 << "static const char\* function\_\_ = \""

3784  << model\_name << "\_namespace::write\_csv(%1%)\";" << EOL;

3785  suppress\_warning(INDENT2, "function\_\_", o);

3786

3787  // declares, reads, and writes parameters

3788  generate\_comment("read-transform, write parameters",2,o);

3789  write\_csv\_visgen vis(o);

3790  for (size\_t i = 0; i < prog.parameter\_decl\_.size(); ++i)

3791  boost::apply\_visitor(vis,prog.parameter\_decl\_[i].decl\_);

3792

3793  // this is for all other values

3794  write\_csv\_vars\_visgen vis\_writer(o);

3795

3796  // parameters are guaranteed to satisfy constraints

3797

3798  o << EOL;

3799  generate\_comment("declare, define and validate transformed parameters",

3800  2,o);

3801  o << INDENT2 << "double lp\_\_ = 0.0;" << EOL;

3802  suppress\_warning(INDENT2, "lp\_\_", o);

3803  o << INDENT2 << "stan::math::accumulator<double> lp\_accum\_\_;" << EOL2;

3804  bool is\_var = false;

3805  bool is\_fun\_return = false;

3806  generate\_local\_var\_decls(prog.derived\_decl\_.first,2,o,

3807  is\_var,is\_fun\_return);

3808  o << EOL;

3809  bool include\_sampling = false;

3810  generate\_statements(prog.derived\_decl\_.second,2,o,include\_sampling,

3811  is\_var,is\_fun\_return);

3812  o << EOL;

3813

3814  generate\_validate\_var\_decls(prog.derived\_decl\_.first,2,o);

3815  o << EOL;

3816

3817  generate\_comment("write transformed parameters",2,o);

3818  for (size\_t i = 0; i < prog.derived\_decl\_.first.size(); ++i)

3819  boost::apply\_visitor(vis\_writer, prog.derived\_decl\_.first[i].decl\_);

3820  o << EOL;

3821

3822  generate\_comment("declare and define generated quantities",2,o);

3823  generate\_local\_var\_decls(prog.generated\_decl\_.first,2,o,is\_var,is\_fun\_return);

3824  o << EOL;

3825  generate\_statements(prog.generated\_decl\_.second,2,o,include\_sampling,

3826  is\_var,is\_fun\_return);

3827  o << EOL;

3828

3829  generate\_comment("validate generated quantities",2,o);

3830  generate\_validate\_var\_decls(prog.generated\_decl\_.first,2,o);

3831  o << EOL;

3832

3833  generate\_comment("write generated quantities",2,o);

3834  for (size\_t i = 0; i < prog.generated\_decl\_.first.size(); ++i)

3835  boost::apply\_visitor(vis\_writer, prog.generated\_decl\_.first[i].decl\_);

3836  if (prog.generated\_decl\_.first.size() > 0)

3837  o << EOL;

3838

3839  o << INDENT2 << "writer\_\_.newline();" << EOL;

3840  o << INDENT << "}" << EOL2;

3841

3842  o << INDENT << "template <typename RNG>" << EOL;

3843  o << INDENT << "void write\_csv(RNG& base\_rng," << EOL;

3844  o << INDENT << " Eigen::Matrix<double,Eigen::Dynamic,1>& params\_r," << EOL;

3845  o << INDENT << " std::ostream& o," << EOL;

3846  o << INDENT << " std::ostream\* pstream = 0) const {" << EOL;

3847  o << INDENT << " std::vector<double> params\_r\_vec(params\_r.size());" << EOL;

3848  o << INDENT << " for (int i = 0; i < params\_r.size(); ++i)" << EOL;

3849  o << INDENT << " params\_r\_vec[i] = params\_r(i);" << EOL;

3850  o << INDENT << " std::vector<int> params\_i\_vec; // dummy" << EOL;

3851  o << INDENT << " write\_csv(base\_rng, params\_r\_vec, params\_i\_vec, o, pstream);" << EOL;

3852  o << INDENT << "}" << EOL2;

3853  }

3854

3855

3856  // see init\_member\_var\_visgen for cut & paste

3857  struct write\_array\_visgen : public visgen {

3858  write\_array\_visgen(std::ostream& o)

3859  : visgen(o) {

3860  }

3861  void operator()(const nil& /\*x\*/) const { }

3862  void operator()(const int\_var\_decl& x) const {

3863  generate\_initialize\_array("int","integer",EMPTY\_EXP\_VECTOR,

3864  x.name\_,x.dims\_);

3865  }

3866  // fixme -- reuse cut-and-pasted from other lub reader case

3867  template <typename D>

3868  void generate\_initialize\_array\_bounded(const D& x, const std::string& base\_type,

3869  const std::string& read\_fun\_prefix,

3870  const std::vector<expression>& dim\_args) const {

3871  std::vector<expression> read\_args;

3872  std::string read\_fun(read\_fun\_prefix);

3873  if (has\_lub(x)) {

3874  read\_fun += "\_lub";

3875  read\_args.push\_back(x.range\_.low\_);

3876  read\_args.push\_back(x.range\_.high\_);

3877  } else if (has\_lb(x)) {

3878  read\_fun += "\_lb";

3879  read\_args.push\_back(x.range\_.low\_);

3880  } else if (has\_ub(x)) {

3881  read\_fun += "\_ub";

3882  read\_args.push\_back(x.range\_.high\_);

3883  }

3884  for (size\_t i = 0; i < dim\_args.size(); ++i)

3885  read\_args.push\_back(dim\_args[i]);

3886  generate\_initialize\_array(base\_type,read\_fun,read\_args,x.name\_,x.dims\_);

3887  }

3888

3889  void operator()(const double\_var\_decl& x) const {

3890  std::vector<expression> read\_args;

3891  generate\_initialize\_array\_bounded(x,"double","scalar",read\_args);

3892  }

3893  void operator()(const vector\_var\_decl& x) const {

3894  std::vector<expression> read\_args;

3895  read\_args.push\_back(x.M\_);

3896  generate\_initialize\_array\_bounded(x,"vector\_d","vector",read\_args);

3897  }

3898  void operator()(const row\_vector\_var\_decl& x) const {

3899  std::vector<expression> read\_args;

3900  read\_args.push\_back(x.N\_);

3901  generate\_initialize\_array\_bounded(x,"row\_vector\_d","row\_vector",read\_args);

3902  }

3903  void operator()(const matrix\_var\_decl& x) const {

3904  std::vector<expression> read\_args;

3905  read\_args.push\_back(x.M\_);

3906  read\_args.push\_back(x.N\_);

3907  generate\_initialize\_array\_bounded(x,"matrix\_d","matrix",read\_args);

3908  }

3909  void operator()(const unit\_vector\_var\_decl& x) const {

3910  std::vector<expression> read\_args;

3911  read\_args.push\_back(x.K\_);

3912  generate\_initialize\_array("vector\_d","unit\_vector",read\_args,x.name\_,x.dims\_);

3913  }

3914  void operator()(const simplex\_var\_decl& x) const {

3915  std::vector<expression> read\_args;

3916  read\_args.push\_back(x.K\_);

3917  generate\_initialize\_array("vector\_d","simplex",read\_args,x.name\_,x.dims\_);

3918  }

3919  void operator()(const ordered\_var\_decl& x) const {

3920  std::vector<expression> read\_args;

3921  read\_args.push\_back(x.K\_);

3922  generate\_initialize\_array("vector\_d","ordered",read\_args,x.name\_,x.dims\_);

3923  }

3924  void operator()(const positive\_ordered\_var\_decl& x) const {

3925  std::vector<expression> read\_args;

3926  read\_args.push\_back(x.K\_);

3927  generate\_initialize\_array("vector\_d","positive\_ordered",read\_args,x.name\_,x.dims\_);

3928  }

3929  void operator()(const cholesky\_factor\_var\_decl& x) const {

3930  std::vector<expression> read\_args;

3931  read\_args.push\_back(x.M\_);

3932  read\_args.push\_back(x.N\_);

3933  generate\_initialize\_array("matrix\_d","cholesky\_factor",read\_args,x.name\_,x.dims\_);

3934  }

3935  void operator()(const cholesky\_corr\_var\_decl& x) const {

3936  std::vector<expression> read\_args;

3937  read\_args.push\_back(x.K\_);

3938  generate\_initialize\_array("matrix\_d","cholesky\_corr",read\_args,x.name\_,x.dims\_);

3939  }

3940  void operator()(const cov\_matrix\_var\_decl& x) const {

3941  std::vector<expression> read\_args;

3942  read\_args.push\_back(x.K\_);

3943  generate\_initialize\_array("matrix\_d","cov\_matrix",read\_args,x.name\_,x.dims\_);

3944  }

3945  void operator()(const corr\_matrix\_var\_decl& x) const {

3946  std::vector<expression> read\_args;

3947  read\_args.push\_back(x.K\_);

3948  generate\_initialize\_array("matrix\_d","corr\_matrix",read\_args,x.name\_,x.dims\_);

3949  }

3950  void generate\_initialize\_array(const std::string& var\_type,

3951  const std::string& read\_type,

3952  const std::vector<expression>& read\_args,

3953  const std::string& name,

3954  const std::vector<expression>& dims) const {

3955  if (dims.size() == 0) {

3956  generate\_indent(2,o\_);

3957  o\_ << var\_type << " ";

3958  o\_ << name << " = in\_\_." << read\_type << "\_constrain(";

3959  for (size\_t j = 0; j < read\_args.size(); ++j) {

3960  if (j > 0) o\_ << ",";

3961  generate\_expression(read\_args[j],o\_);

3962  }

3963  o\_ << ");" << EOL;

3964  return;

3965  }

3966  o\_ << INDENT2;

3967  for (size\_t i = 0; i < dims.size(); ++i) o\_ << "vector<";

3968  o\_ << var\_type;

3969  for (size\_t i = 0; i < dims.size(); ++i) o\_ << "> ";

3970  o\_ << name << ";" << EOL;

3971  std::string name\_dims(name);

3972  for (size\_t i = 0; i < dims.size(); ++i) {

3973  generate\_indent(i + 2, o\_);

3974  o\_ << "size\_t dim\_" << name << "\_" << i << "\_\_ = ";

3975  generate\_expression(dims[i],o\_);

3976  o\_ << ";" << EOL;

3977  if (i < dims.size() - 1) {

3978  generate\_indent(i + 2, o\_);

3979  o\_ << name\_dims << ".resize(dim\_" << name << "\_" << i << "\_\_);"

3980  << EOL;

3981  name\_dims.append("[k\_").append(to\_string(i)).append("\_\_]");

3982  }

3983  generate\_indent(i + 2, o\_);

3984  o\_ << "for (size\_t k\_" << i << "\_\_ = 0;"

3985  << " k\_" << i << "\_\_ < dim\_" << name << "\_" << i << "\_\_;"

3986  << " ++k\_" << i << "\_\_) {" << EOL;

3987  if (i == dims.size() - 1) {

3988  generate\_indent(i + 3, o\_);

3989  o\_ << name\_dims << ".push\_back(in\_\_." << read\_type << "\_constrain(";

3990  for (size\_t j = 0; j < read\_args.size(); ++j) {

3991  if (j > 0) o\_ << ",";

3992  generate\_expression(read\_args[j],o\_);

3993  }

3994  o\_ << "));" << EOL;

3995  }

3996  }

3997

3998  for (size\_t i = dims.size(); i > 0; --i) {

3999  generate\_indent(i + 1, o\_);

4000  o\_ << "}" << EOL;

4001  }

4002

4003

4004  }

4005  };

4006

4007

4008

4009

4010  struct write\_array\_vars\_visgen : public visgen {

4011  write\_array\_vars\_visgen(std::ostream& o)

4012  : visgen(o) {

4013  }

4014  void operator()(const nil& /\*x\*/) const { }

4015  // FIXME: template these out

4016  void operator()(const int\_var\_decl& x) const {

4017  write\_array(x.name\_,x.dims\_,EMPTY\_EXP\_VECTOR);

4018  }

4019  void operator()(const double\_var\_decl& x) const {

4020  write\_array(x.name\_,x.dims\_,EMPTY\_EXP\_VECTOR);

4021  }

4022  void operator()(const vector\_var\_decl& x) const {

4023  std::vector<expression> dims(x.dims\_);

4024  dims.push\_back(x.M\_);

4025  write\_array(x.name\_,dims, EMPTY\_EXP\_VECTOR);

4026  }

4027  void operator()(const row\_vector\_var\_decl& x) const {

4028  std::vector<expression> dims(x.dims\_);

4029  dims.push\_back(x.N\_);

4030  write\_array(x.name\_,dims, EMPTY\_EXP\_VECTOR);

4031  }

4032  void operator()(const matrix\_var\_decl& x) const {

4033  std::vector<expression> matdims;

4034  matdims.push\_back(x.M\_);

4035  matdims.push\_back(x.N\_);

4036  write\_array(x.name\_,x.dims\_,matdims);

4037  }

4038  void operator()(const unit\_vector\_var\_decl& x) const {

4039  std::vector<expression> dims(x.dims\_);

4040  dims.push\_back(x.K\_);

4041  write\_array(x.name\_,dims,EMPTY\_EXP\_VECTOR);

4042  }

4043  void operator()(const simplex\_var\_decl& x) const {

4044  std::vector<expression> dims(x.dims\_);

4045  dims.push\_back(x.K\_);

4046  write\_array(x.name\_,dims,EMPTY\_EXP\_VECTOR);

4047  }

4048  void operator()(const ordered\_var\_decl& x) const {

4049  std::vector<expression> dims(x.dims\_);

4050  dims.push\_back(x.K\_);

4051  write\_array(x.name\_,dims,EMPTY\_EXP\_VECTOR);

4052  }

4053  void operator()(const positive\_ordered\_var\_decl& x) const {

4054  std::vector<expression> dims(x.dims\_);

4055  dims.push\_back(x.K\_);

4056  write\_array(x.name\_,dims,EMPTY\_EXP\_VECTOR);

4057  }

4058  void operator()(const cholesky\_factor\_var\_decl& x) const {

4059  std::vector<expression> matdims;

4060  matdims.push\_back(x.M\_);

4061  matdims.push\_back(x.N\_);

4062  write\_array(x.name\_,x.dims\_,matdims);

4063  }

4064  void operator()(const cholesky\_corr\_var\_decl& x) const {

4065  std::vector<expression> matdims;

4066  matdims.push\_back(x.K\_);

4067  matdims.push\_back(x.K\_);

4068  write\_array(x.name\_,x.dims\_,matdims);

4069  }

4070  void operator()(const cov\_matrix\_var\_decl& x) const {

4071  std::vector<expression> matdims;

4072  matdims.push\_back(x.K\_);

4073  matdims.push\_back(x.K\_);

4074  write\_array(x.name\_,x.dims\_,matdims);

4075  }

4076  void operator()(const corr\_matrix\_var\_decl& x) const {

4077  std::vector<expression> matdims;

4078  matdims.push\_back(x.K\_);

4079  matdims.push\_back(x.K\_);

4080  write\_array(x.name\_,x.dims\_,matdims);

4081  }

4082  void write\_array(const std::string& name,

4083  const std::vector<expression>& arraydims,

4084  const std::vector<expression>& matdims) const {

4085

4086  std::vector<expression> dims(arraydims);

4087  for (size\_t i = 0; i < matdims.size(); ++i)

4088  dims.push\_back(matdims[i]);

4089

4090  if (dims.size() == 0) {

4091  o\_ << INDENT2 << "vars\_\_.push\_back(" << name << ");" << EOL;

4092  return;

4093  }

4094

4095  // for (size\_t i = 0; i < dims.size(); ++i) {

4096  for (size\_t i = dims.size(); i > 0; ) {

4097  --i;

4098  generate\_indent((dims.size() - i) + 1, o\_);

4099  o\_ << "for (int k\_" << i << "\_\_ = 0;"

4100  << " k\_" << i << "\_\_ < ";

4101  generate\_expression(dims[i],o\_);

4102  o\_ << "; ++k\_" << i << "\_\_) {" << EOL;

4103  }

4104

4105  generate\_indent(dims.size() + 2, o\_);

4106  o\_ << "vars\_\_.push\_back(" << name;

4107  if (arraydims.size() > 0) {

4108  o\_ << '[';

4109  for (size\_t i = 0; i < arraydims.size(); ++i) {

4110  if (i > 0) o\_ << "][";

4111  o\_ << "k\_" << i << "\_\_";

4112  }

4113  o\_ << ']';

4114  }

4115  if (matdims.size() > 0) {

4116  o\_ << "(k\_" << arraydims.size() << "\_\_";

4117  if (matdims.size() > 1)

4118  o\_ << ", k\_" << (arraydims.size() + 1) << "\_\_";

4119  o\_ << ")";

4120  }

4121  o\_ << ");" << EOL;

4122

4123  for (size\_t i = dims.size(); i > 0; --i) {

4124  generate\_indent(i + 1, o\_);

4125  o\_ << "}" << EOL;

4126  }

4127  }

4128  };

4129

4130

4131  void generate\_write\_array\_method(const program& prog,

4132  const std::string& model\_name,

4133  std::ostream& o) {

4134  o << INDENT << "template <typename RNG>" << EOL;

4135  o << INDENT << "void write\_array(RNG& base\_rng\_\_," << EOL;

4136  o << INDENT << " std::vector<double>& params\_r\_\_," << EOL;

4137  o << INDENT << " std::vector<int>& params\_i\_\_," << EOL;

4138  o << INDENT << " std::vector<double>& vars\_\_," << EOL;

4139  o << INDENT << " bool include\_tparams\_\_ = true," << EOL;

4140  o << INDENT << " bool include\_gqs\_\_ = true," << EOL;

4141  o << INDENT << " std::ostream\* pstream\_\_ = 0) const {" << EOL;

4142  o << INDENT2 << "vars\_\_.resize(0);" << EOL;

4143  o << INDENT2 << "stan::io::reader<double> in\_\_(params\_r\_\_,params\_i\_\_);" << EOL;

4144  o << INDENT2 << "static const char\* function\_\_ = \""

4145  << model\_name << "\_namespace::write\_array(%1%)\";" << EOL;

4146  suppress\_warning(INDENT2, "function\_\_", o);

4147

4148  // declares, reads, and sets parameters

4149  generate\_comment("read-transform, write parameters",2,o);

4150  write\_array\_visgen vis(o);

4151  for (size\_t i = 0; i < prog.parameter\_decl\_.size(); ++i)

4152  boost::apply\_visitor(vis,prog.parameter\_decl\_[i].decl\_);

4153

4154  // this is for all other values

4155  write\_array\_vars\_visgen vis\_writer(o);

4156

4157  // writes parameters

4158  for (size\_t i = 0; i < prog.parameter\_decl\_.size(); ++i)

4159  boost::apply\_visitor(vis\_writer,prog.parameter\_decl\_[i].decl\_);

4160  o << EOL;

4161

4162  o << INDENT2 << "if (!include\_tparams\_\_) return;"

4163  << EOL;

4164  generate\_comment("declare and define transformed parameters",2,o);

4165  o << INDENT2 << "double lp\_\_ = 0.0;" << EOL;

4166  suppress\_warning(INDENT2, "lp\_\_", o);

4167  o << INDENT2 << "stan::math::accumulator<double> lp\_accum\_\_;" << EOL2;

4168  bool is\_var = false;

4169  bool is\_fun\_return = false;

4170  generate\_local\_var\_decls(prog.derived\_decl\_.first,2,o,is\_var,is\_fun\_return);

4171  o << EOL;

4172  bool include\_sampling = false;

4173  generate\_statements(prog.derived\_decl\_.second,2,o,include\_sampling,

4174  is\_var,is\_fun\_return);

4175  o << EOL;

4176

4177  generate\_comment("validate transformed parameters",2,o);

4178  generate\_validate\_var\_decls(prog.derived\_decl\_.first,2,o);

4179  o << EOL;

4180

4181  generate\_comment("write transformed parameters",2,o);

4182  for (size\_t i = 0; i < prog.derived\_decl\_.first.size(); ++i)

4183  boost::apply\_visitor(vis\_writer, prog.derived\_decl\_.first[i].decl\_);

4184  o << EOL;

4185

4186  o << INDENT2 << "if (!include\_gqs\_\_) return;"

4187  << EOL;

4188  generate\_comment("declare and define generated quantities",2,o);

4189  generate\_local\_var\_decls(prog.generated\_decl\_.first,2,o,

4190  is\_var,is\_fun\_return);

4191  o << EOL;

4192  generate\_statements(prog.generated\_decl\_.second,2,o,include\_sampling,

4193  is\_var,is\_fun\_return);

4194  o << EOL;

4195

4196  generate\_comment("validate generated quantities",2,o);

4197  generate\_validate\_var\_decls(prog.generated\_decl\_.first,2,o);

4198  o << EOL;

4199

4200  generate\_comment("write generated quantities",2,o);

4201  for (size\_t i = 0; i < prog.generated\_decl\_.first.size(); ++i)

4202  boost::apply\_visitor(vis\_writer, prog.generated\_decl\_.first[i].decl\_);

4203  if (prog.generated\_decl\_.first.size() > 0)

4204  o << EOL;

4205

4206  o << INDENT << "}" << EOL2;

4207

4208  o << INDENT << "template <typename RNG>" << EOL;

4209  o << INDENT << "void write\_array(RNG& base\_rng," << EOL;

4210  o << INDENT << " Eigen::Matrix<double,Eigen::Dynamic,1>& params\_r," << EOL;

4211  o << INDENT << " Eigen::Matrix<double,Eigen::Dynamic,1>& vars," << EOL;

4212  o << INDENT << " bool include\_tparams = true," << EOL;

4213  o << INDENT << " bool include\_gqs = true," << EOL;

4214  o << INDENT << " std::ostream\* pstream = 0) const {" << EOL;

4215  o << INDENT << " std::vector<double> params\_r\_vec(params\_r.size());" << EOL;

4216  o << INDENT << " for (int i = 0; i < params\_r.size(); ++i)" << EOL;

4217  o << INDENT << " params\_r\_vec[i] = params\_r(i);" << EOL;

4218  o << INDENT << " std::vector<double> vars\_vec;" << EOL;

4219  o << INDENT << " std::vector<int> params\_i\_vec;" << EOL;

4220  o << INDENT << " write\_array(base\_rng,params\_r\_vec,params\_i\_vec,vars\_vec,include\_tparams,include\_gqs,pstream);" << EOL;

4221  o << INDENT << " vars.resize(vars\_vec.size());" << EOL;

4222  o << INDENT << " for (int i = 0; i < vars.size(); ++i)" << EOL;

4223  o << INDENT << " vars(i) = vars\_vec[i];" << EOL;

4224  o << INDENT << "}" << EOL2;

4225

4226  }

4227

4228  void generate\_model\_name\_method(const std::string& model\_name,

4229  std::ostream& out) {

4230  out << INDENT << "static std::string model\_name() {" << EOL

4231  << INDENT2 << "return \"" << model\_name << "\";" << EOL

4232  << INDENT << "}" << EOL2;

4233  }

4234

4235  void generate\_model\_typedef(const std::string& model\_name,

4236  std::ostream& out) {

4237  out << "typedef " << model\_name << "\_namespace::" << model\_name

4238  << " stan\_model;" <<EOL2;

4239  }

4240

4241  void generate\_bare\_type(const expr\_type& t,

4242  const std::string& scalar\_t\_name,

4243  std::ostream& out) {

4244  for (size\_t d = 0; d < t.num\_dims\_; ++d)

4245  out << "std::vector<";

4246

4247  bool is\_template\_type = false;

4248  switch (t.base\_type\_) {

4249  case INT\_T :

4250  out << "int";

4251  is\_template\_type = false;

4252  break;

4253  case DOUBLE\_T:

4254  out << scalar\_t\_name;

4255  is\_template\_type = false;

4256  break;

4257  case VECTOR\_T:

4258  out << "Eigen::Matrix<"

4259  << scalar\_t\_name

4260  << ", Eigen::Dynamic,1>";

4261  is\_template\_type = true;

4262  break;

4263  case ROW\_VECTOR\_T:

4264  out << "Eigen::Matrix<"

4265  << scalar\_t\_name

4266  << ", 1,Eigen::Dynamic>";

4267  is\_template\_type = true;

4268  break;

4269  case MATRIX\_T:

4270  out << "Eigen::Matrix<"

4271  << scalar\_t\_name

4272  << ", Eigen::Dynamic,Eigen::Dynamic>";

4273  is\_template\_type = true;

4274  break;

4275  case VOID\_T:

4276  out << "void";

4277  break;

4278  default:

4279  out << "UNKNOWN TYPE";

4280  }

4281

4282  for (size\_t d = 0; d < t.num\_dims\_; ++d) {

4283  if (d > 0 || is\_template\_type)

4284  out << " ";

4285  out << ">";

4286  }

4287  }

4288

4289  void generate\_arg\_decl(bool gen\_const,

4290  bool gen\_ref,

4291  const arg\_decl& decl,

4292  const std::string& scalar\_t\_name,

4293  std::ostream& out) {

4294  if (gen\_const)

4295  out << "const ";

4296  generate\_bare\_type(decl.arg\_type\_,scalar\_t\_name,out);

4297  if (gen\_ref)

4298  out << "&";

4299  out << " " << decl.name\_;

4300  }

4301

4302  bool has\_only\_int\_args(const function\_decl\_def& fun) {

4303  for (size\_t i = 0; i < fun.arg\_decls\_.size(); ++i)

4304  if (fun.arg\_decls\_[i].arg\_type\_.base\_type\_ != INT\_T)

4305  return false;

4306  return true;

4307  }

4308

4309  std::string fun\_scalar\_type(const function\_decl\_def& fun,

4310  bool is\_lp) {

4311  size\_t num\_args = fun.arg\_decls\_.size();

4312  // nullary, non-lp

4313  if (has\_only\_int\_args(fun) && !is\_lp)

4314  return "double";

4315

4316  // need template metaprogram to construct return

4317  std::stringstream ss;

4318  ss << "typename boost::math::tools::promote\_args<";

4319  int num\_open\_brackets = 1;

4320  int num\_generated\_params = 0;

4321  for (size\_t i = 0; i < num\_args; ++i) {

4322  if (fun.arg\_decls\_[i].arg\_type\_.base\_type\_ != INT\_T) {

4323  // two conditionals cut and pasted below

4324  if (num\_generated\_params > 0)

4325  ss << ", ";

4326  if (num\_generated\_params == 4) {

4327  ss << "typename boost::math::tools::promote\_args<";

4328  num\_generated\_params = 0;

4329  ++num\_open\_brackets;

4330  }

4331  ss << "T" << i << "\_\_";

4332  ++num\_generated\_params;

4333  }

4334  }

4335  if (is\_lp) {

4336  if (num\_generated\_params > 0)

4337  ss << ", ";

4338  // set threshold at 4 so always room for one more param at end

4339  ss << "T\_lp\_\_";

4340  }

4341  for (int i = 0; i < num\_open\_brackets; ++i)

4342  ss << ">::type";

4343  return ss.str();

4344  }

4345

4346  bool needs\_template\_params(const function\_decl\_def& fun) {

4347  for (size\_t i = 0; i < fun.arg\_decls\_.size(); ++i) {

4348  if (fun.arg\_decls\_[i].arg\_type\_.base\_type\_ != INT\_T) {

4349  return true;

4350  }

4351  }

4352  return false;

4353  }

4354

4355

4356  void generate\_function\_template\_parameters(const function\_decl\_def& fun,

4357  bool is\_rng,

4358  bool is\_lp,

4359  bool is\_log,

4360  std::ostream& out) {

4361  if (needs\_template\_params(fun)) {

4362  out << "template <";

4363  bool continuing\_tps = false;

4364  if (is\_log) {

4365  out << "bool propto";

4366  continuing\_tps = true;

4367  }

4368  for (size\_t i = 0; i < fun.arg\_decls\_.size(); ++i) {

4369  // no template parameter for int-based args

4370  if (fun.arg\_decls\_[i].arg\_type\_.base\_type\_ != INT\_T) {

4371  if (continuing\_tps)

4372  out << ", ";

4373  out << "typename T" << i << "\_\_";

4374  continuing\_tps = true;

4375  }

4376  }

4377  if (is\_rng) {

4378  if (continuing\_tps)

4379  out << ", ";

4380  out << "class RNG";

4381  continuing\_tps = true;

4382  }

4383  else if (is\_lp) {

4384  if (continuing\_tps)

4385  out << ", ";

4386  out << "typename T\_lp\_\_, typename T\_lp\_accum\_\_";

4387  continuing\_tps = true;

4388  }

4389  out << ">" << EOL;

4390  } else { // no-arg function

4391  if (is\_rng) {

4392  // nullary RNG case

4393  out << "template <class RNG>" << EOL;

4394  } else if (is\_lp) {

4395  out << "template <typename T\_lp\_\_, typename T\_lp\_accum\_\_>"

4396  << EOL;

4397  } else if (is\_log) {

4398  out << "template <bool propto>"

4399  << EOL;

4400  }

4401  }

4402  }

4403

4404  void generate\_function\_inline\_return\_type(const function\_decl\_def& fun,

4405  const std::string& scalar\_t\_name,

4406  std::ostream& out) {

4407  out << "inline" << EOL;

4408  generate\_bare\_type(fun.return\_type\_,scalar\_t\_name,out);

4409  out << EOL;

4410  }

4411

4412  void generate\_function\_name(const function\_decl\_def& fun,

4413  std::ostream& out) {

4414  out << fun.name\_;

4415  }

4416

4417

4418  void generate\_function\_arguments(const function\_decl\_def& fun,

4419  bool is\_rng,

4420  bool is\_lp,

4421  bool is\_log,

4422  std::ostream& out) {

4423  // arguments

4424  out << "(";

4425  for (size\_t i = 0; i < fun.arg\_decls\_.size(); ++i) {

4426  std::string template\_type\_i

4427  = "T" + boost::lexical\_cast<std::string>(i) + "\_\_";

4428  generate\_arg\_decl(true,true,fun.arg\_decls\_[i],template\_type\_i,out);

4429  if (i + 1 < fun.arg\_decls\_.size()) {

4430  out << "," << EOL << INDENT;

4431  for (size\_t i = 0; i <= fun.name\_.size(); ++i)

4432  out << " ";

4433  }

4434  }

4435  if ((is\_rng || is\_lp) && fun.arg\_decls\_.size() > 0)

4436  out << ", ";

4437  if (is\_rng)

4438  out << "RNG& base\_rng\_\_";

4439  else if (is\_lp)

4440  out << "T\_lp\_\_& lp\_\_, T\_lp\_accum\_\_& lp\_accum\_\_";

4441  if (is\_rng || is\_lp || fun.arg\_decls\_.size() > 0)

4442  out << ", ";

4443  out << "std::ostream\* pstream\_\_";

4444  out << ")";

4445  }

4446

4447  void generate\_functor\_arguments(const function\_decl\_def& fun,

4448  bool is\_rng,

4449  bool is\_lp,

4450  bool is\_log,

4451  std::ostream& out) {

4452  // arguments

4453  out << "(";

4454  for (size\_t i = 0; i < fun.arg\_decls\_.size(); ++i) {

4455  if (i > 0)

4456  out << ", ";

4457  out << fun.arg\_decls\_[i].name\_;

4458  }

4459  if ((is\_rng || is\_lp) && fun.arg\_decls\_.size() > 0)

4460  out << ", ";

4461  if (is\_rng)

4462  out << "base\_rng\_\_";

4463  else if (is\_lp)

4464  out << "lp\_\_, lp\_accum\_\_";

4465  if (is\_rng || is\_lp || fun.arg\_decls\_.size() > 0)

4466  out << ", ";

4467  out << "pstream\_\_";

4468  out << ")";

4469  }

4470

4471

4472

4473  void generate\_function\_body(const function\_decl\_def& fun,

4474  const std::string& scalar\_t\_name,

4475  std::ostream& out) {

4476  // no-op body

4477  if (fun.body\_.is\_no\_op\_statement()) {

4478  out << ";" << EOL;

4479  return;

4480  }

4481  out << " {" << EOL;

4482  out << INDENT

4483  << "typedef " << scalar\_t\_name << " fun\_scalar\_t\_\_;"

4484  << EOL;

4485  out << INDENT

4486  << "typedef "

4487  << ((fun.return\_type\_.base\_type\_ == INT\_T)

4488  ? "int" : "fun\_scalar\_t\_\_")

4489  << " fun\_return\_scalar\_t\_\_;"

4490  << EOL;

4491  out << INDENT

4492  << "const static bool propto\_\_ = true;"

4493  << EOL

4494  << INDENT

4495  << "(void) propto\_\_;"

4496  << EOL;

4497  bool is\_var = false;

4498  bool is\_fun\_return = true;

4499  bool include\_sampling = true;

4500  generate\_statement(fun.body\_,1,out,

4501  include\_sampling,is\_var,is\_fun\_return);

4502  out << "}"

4503  << EOL;

4504  }

4505  void generate\_propto\_default\_function\_body(const function\_decl\_def& fun,

4506  std::ostream& out) {

4507  out << " {" << EOL;

4508  out << INDENT << "return ";

4509  out << fun.name\_ << "<false>(";

4510  for (size\_t i = 0; i < fun.arg\_decls\_.size(); ++i) {

4511  if (i > 0)

4512  out << ",";

4513  out << fun.arg\_decls\_[i].name\_;

4514  }

4515  if (fun.arg\_decls\_.size() > 0)

4516  out << ", ";

4517  out << "pstream\_\_";

4518  out << ");" << EOL;

4519  out << "}" << EOL;

4520  }

4521

4522  void generate\_propto\_default\_function(const function\_decl\_def& fun,

4523  const std::string& scalar\_t\_name,

4524  std::ostream& out) {

4525  generate\_function\_template\_parameters(fun,false,false,false,out);

4526  generate\_function\_inline\_return\_type(fun,scalar\_t\_name,out);

4527  generate\_function\_name(fun,out);

4528  generate\_function\_arguments(fun,false,false,false,out);

4529  generate\_propto\_default\_function\_body(fun,out);

4530  }

4531

4543  void generate\_function(const function\_decl\_def& fun,

4544  std::ostream& out) {

4545

4546  bool is\_rng = ends\_with("\_rng", fun.name\_);

4547  bool is\_lp = ends\_with("\_lp", fun.name\_);

4548  bool is\_log = ends\_with("\_log", fun.name\_);

4549  std::string scalar\_t\_name

4550  = fun\_scalar\_type(fun, is\_lp);

4551

4552  generate\_function\_template\_parameters(fun,is\_rng,is\_lp,is\_log,out);

4553  generate\_function\_inline\_return\_type(fun,scalar\_t\_name,out);

4554  generate\_function\_name(fun,out);

4555  generate\_function\_arguments(fun,is\_rng,is\_lp,is\_log,out);

4556  generate\_function\_body(fun,scalar\_t\_name,out);

4557

4558  // need a second function def for default propto=false for \_log funs

4559  if (is\_log)

4560  generate\_propto\_default\_function(fun,scalar\_t\_name,out);

4561  out << EOL;

4562  }

4563

4564  void generate\_function\_functor(const function\_decl\_def& fun,

4565  std::ostream& out) {

4566  if (fun.body\_.is\_no\_op\_statement())

4567  return; // forward declaration, so no functor needed

4568

4569  bool is\_rng = ends\_with("\_rng", fun.name\_);

4570  bool is\_lp = ends\_with("\_lp", fun.name\_);

4571  bool is\_log = ends\_with("\_log", fun.name\_);

4572  std::string scalar\_t\_name

4573  = fun\_scalar\_type(fun, is\_lp);

4574

4575  out << std::endl

4576  << "struct ";

4577  generate\_function\_name(fun,out);

4578  out << "\_functor\_\_ {"

4579  << std::endl;

4580

4581  generate\_function\_template\_parameters(fun,is\_rng,is\_lp,is\_log,out);

4582

4583  generate\_function\_inline\_return\_type(fun,scalar\_t\_name,out);

4584

4585  out << "operator()";

4586  generate\_function\_arguments(fun,is\_rng,is\_lp,is\_log,out);

4587  out << " const {"

4588  << std::endl;

4589

4590  out << INDENT

4591  << "return ";

4592  generate\_function\_name(fun,out);

4593  generate\_functor\_arguments(fun,is\_rng,is\_lp,is\_log,out);

4594  out << ";"

4595  << std::endl;

4596

4597  out << "}"

4598  << std::endl;

4599

4600  out << "};"

4601  << std::endl

4602  << std::endl;

4603  }

4604

4605

4606  void generate\_functions(const std::vector<function\_decl\_def>& funs,

4607  std::ostream& out) {

4608  for (size\_t i = 0; i < funs.size(); ++i) {

4609  generate\_function(funs[i],out);

4610  generate\_function\_functor(funs[i],out);

4611  }

4612  }

4613

4614  void generate\_cpp(const program& prog,

4615  const std::string& model\_name,

4616  std::ostream& out) {

4617  generate\_version\_comment(out);

4618  generate\_includes(out);

4619  generate\_start\_namespace(model\_name,out);

4620  generate\_usings(out);

4621  generate\_typedefs(out);

4622  generate\_functions(prog.function\_decl\_defs\_,out);

4623  generate\_class\_decl(model\_name,out);

4624  generate\_private\_decl(out);

4625  generate\_member\_var\_decls(prog.data\_decl\_,1,out);

4626  generate\_member\_var\_decls(prog.derived\_data\_decl\_.first,1,out);

4627  generate\_public\_decl(out);

4628  generate\_constructor(prog,model\_name,out);

4629  generate\_destructor(model\_name,out);

4630  // generate\_set\_param\_ranges(prog.parameter\_decl\_,out);

4631  generate\_init\_method(prog.parameter\_decl\_,out);

4632  generate\_log\_prob(prog,out);

4633  generate\_param\_names\_method(prog,out);

4634  generate\_dims\_method(prog,out);

4635  generate\_write\_array\_method(prog,model\_name,out);

4636  generate\_write\_csv\_header\_method(prog,out);

4637  generate\_write\_csv\_method(prog,model\_name,out);

4638  generate\_model\_name\_method(model\_name,out);

4639  generate\_constrained\_param\_names\_method(prog,out);

4640  generate\_unconstrained\_param\_names\_method(prog,out);

4641  generate\_end\_class\_decl(out);

4642  generate\_end\_namespace(out);

4643  generate\_model\_typedef(model\_name,out);

4644  }

4645

4646  }

4647

4648 }

4649

4650 #endif

stan::gm::init\_local\_var\_visgen::operator()

void operator()(const positive\_ordered\_var\_decl &x) const

**Definition:** generator.hpp:616

stan::gm::visgen

generic visitor with output for extension

**Definition:** generator.hpp:64

stan::gm::generate\_local\_var\_init\_nan\_visgen::operator()

void operator()(const double\_var\_decl &x) const

**Definition:** generator.hpp:1249

stan::gm::write\_csv\_vars\_visgen::operator()

void operator()(const simplex\_var\_decl &x) const

**Definition:** generator.hpp:3716

stan::gm::generate\_init\_visgen::operator()

void operator()(cholesky\_corr\_var\_decl const &x) const

**Definition:** generator.hpp:2666

stan::gm::distribution::family\_

std::string family\_

**Definition:** ast.hpp:195

stan::gm::nil

Placeholder struct for boost::variant default ctors.

**Definition:** ast.hpp:18

stan::gm::validate\_var\_decl\_visgen::operator()

void operator()(unit\_vector\_var\_decl const &x) const

**Definition:** generator.hpp:880

stan::gm::var\_resizing\_visgen::operator()

void operator()(nil const &) const

**Definition:** generator.hpp:495

stan::gm::generate\_void\_statement

void generate\_void\_statement(const std::string &name, const size\_t indent, std::ostream &o)

**Definition:** generator.hpp:55

stan::gm::generate\_function\_functor

void generate\_function\_functor(const function\_decl\_def &fun, std::ostream &out)

**Definition:** generator.hpp:4564

stan::gm::var\_resizing\_visgen::operator()

void operator()(corr\_matrix\_var\_decl const &x) const

**Definition:** generator.hpp:532

stan::gm::increment\_log\_prob\_statement::log\_prob\_

expression log\_prob\_

**Definition:** ast.hpp:713

stan::gm::constrained\_param\_names\_visgen::operator()

void operator()(const row\_vector\_var\_decl &x) const

**Definition:** generator.hpp:3212

stan::gm::write\_array\_visgen::generate\_initialize\_array\_bounded

void generate\_initialize\_array\_bounded(const D &x, const std::string &base\_type, const std::string &read\_fun\_prefix, const std::vector< expression > &dim\_args) const

**Definition:** generator.hpp:3868

stan::gm::statement\_visgen::indent\_

size\_t indent\_

**Definition:** generator.hpp:1519

stan::gm::validate\_var\_decl\_visgen::generate\_begin\_for\_dims

void generate\_begin\_for\_dims(const std::vector< expression > &dims) const

**Definition:** generator.hpp:790

stan::gm::generate\_statements

void generate\_statements(const std::vector< statement > &ss, int indent, std::ostream &o, bool include\_sampling, bool is\_var, bool is\_fun\_return)

**Definition:** generator.hpp:1756

stan::gm::statement

**Definition:** ast.hpp:634

stan::gm::local\_var\_decl\_visgen::operator()

void operator()(vector\_var\_decl const &x) const

**Definition:** generator.hpp:1021

stan::gm::generate\_expression

void generate\_expression(const expression &e, std::ostream &o)

**Definition:** generator.hpp:249

stan::gm::validate\_var\_decl\_visgen::operator()

void operator()(simplex\_var\_decl const &x) const

**Definition:** generator.hpp:883

stan::gm::generate\_init\_vars\_visgen::operator()

void operator()(unit\_vector\_var\_decl const &x) const

**Definition:** generator.hpp:1335

stan::gm::var\_resizing\_visgen::operator()

void operator()(vector\_var\_decl const &x) const

**Definition:** generator.hpp:502

stan::gm::validate\_transformed\_params\_visgen::operator()

void operator()(cholesky\_corr\_var\_decl const &x) const

**Definition:** generator.hpp:1439

stan::gm::init\_local\_var\_visgen::operator()

void operator()(const double\_var\_decl &x) const

**Definition:** generator.hpp:581

stan::gm::statement::is\_no\_op\_statement

bool is\_no\_op\_statement() const

**Definition:** ast\_def.cpp:1286

stan::gm::corr\_matrix\_var\_decl

**Definition:** ast.hpp:563

stan::gm::generate\_local\_var\_init\_nan\_visgen::operator()

void operator()(const corr\_matrix\_var\_decl &x) const

**Definition:** generator.hpp:1282

stan::gm::generate\_initialization

void generate\_initialization(std::ostream &o, const std::string &var\_name, const std::string &base\_type, const std::vector< expression > &dims, const expression &type\_arg1=expression(), const expression &type\_arg2=expression())

**Definition:** generator.hpp:469

stan::gm::write\_array\_vars\_visgen::write\_array

void write\_array(const std::string &name, const std::vector< expression > &arraydims, const std::vector< expression > &matdims) const

**Definition:** generator.hpp:4082

stan::gm::ends\_with

bool ends\_with(const std::string &suffix, const std::string &s)

**Definition:** ast\_def.cpp:1477

stan::gm::generate\_init\_visgen::generate\_buffer\_loop

void generate\_buffer\_loop(const std::string &base\_type, const std::string &name, const std::vector< expression > &dims, const expression &dim1=expression(), const expression &dim2=expression(), int indent=2U) const

**Definition:** generator.hpp:2722

stan::gm::generate\_init\_visgen::generate\_dims\_loop\_fwd

void generate\_dims\_loop\_fwd(const std::vector< expression > &dims, int indent=2U) const

**Definition:** generator.hpp:2765

stan::gm::validate\_var\_decl\_visgen::operator()

void operator()(nil const &) const

**Definition:** generator.hpp:815

stan::gm::program::derived\_data\_decl\_

std::pair< std::vector< var\_decl >, std::vector< statement > > derived\_data\_decl\_

**Definition:** ast.hpp:799

stan::gm::write\_csv\_header\_visgen::operator()

void operator()(const cholesky\_factor\_var\_decl &x) const

**Definition:** generator.hpp:3078

stan::gm::suppress\_warning

void suppress\_warning(const std::string &indent, const std::string &var\_name, std::ostream &o)

**Definition:** generator.hpp:2304

stan::gm::conditional\_statement::conditions\_

std::vector< expression > conditions\_

**Definition:** ast.hpp:730

stan::gm::EMPTY\_EXP\_VECTOR

const std::vector< expression > EMPTY\_EXP\_VECTOR(0)

stan::gm::generate\_local\_var\_init\_nan\_visgen::operator()

void operator()(const positive\_ordered\_var\_decl &x) const

**Definition:** generator.hpp:1270

stan::gm::write\_dims\_visgen::operator()

void operator()(const double\_var\_decl &x) const

**Definition:** generator.hpp:2835

stan::gm::row\_vector\_var\_decl::N\_

expression N\_

**Definition:** ast.hpp:516

stan::gm::local\_var\_decl\_visgen::generate\_type

void generate\_type(const std::string &type, size\_t num\_dims) const

**Definition:** generator.hpp:1124

stan::gm::member\_var\_decl\_visgen::operator()

void operator()(cov\_matrix\_var\_decl const &x) const

**Definition:** generator.hpp:954

stan::gm::print\_string\_literal

static void print\_string\_literal(std::ostream &o, const std::string &s)

**Definition:** generator.hpp:254

stan::gm::base\_var\_decl::dims\_

std::vector< expression > dims\_

**Definition:** ast.hpp:430

stan::gm::expr\_type::base\_type\_

base\_expr\_type base\_type\_

**Definition:** ast.hpp:75

stan::gm::generate\_model\_name\_method

void generate\_model\_name\_method(const std::string &model\_name, std::ostream &out)

**Definition:** generator.hpp:4228

stan::gm::var\_resizing\_visgen::operator()

void operator()(double\_var\_decl const &x) const

**Definition:** generator.hpp:499

stan::gm::constrained\_param\_names\_visgen::generate\_param\_names\_array

void generate\_param\_names\_array(const std::vector< expression > &matrix\_dims, const std::string &name, const std::vector< expression > &dims) const

**Definition:** generator.hpp:3268

stan::gm::validate\_var\_decl\_visgen::operator()

void operator()(row\_vector\_var\_decl const &x) const

**Definition:** generator.hpp:862

stan::gm::assignment::var\_dims\_

variable\_dims var\_dims\_

**Definition:** ast.hpp:829

stan::gm::generate\_local\_var\_init\_nan\_visgen::operator()

void operator()(const unit\_vector\_var\_decl &x) const

**Definition:** generator.hpp:1261

stan::gm::double\_var\_decl

**Definition:** ast.hpp:464

stan::gm::write\_csv\_visgen::operator()

void operator()(const cholesky\_factor\_var\_decl &x) const

**Definition:** generator.hpp:3601

stan::gm::generate\_validate\_transformed\_params

void generate\_validate\_transformed\_params(const std::vector< var\_decl > &vs, int indent, std::ostream &o)

**Definition:** generator.hpp:1504

stan::gm::member\_var\_decl\_visgen::operator()

void operator()(row\_vector\_var\_decl const &x) const

**Definition:** generator.hpp:963

stan::gm::generate\_using

void generate\_using(const std::string &type, std::ostream &o)

**Definition:** generator.hpp:288

stan::gm::corr\_matrix\_var\_decl::K\_

expression K\_

**Definition:** ast.hpp:564

stan::gm::write\_array\_visgen::operator()

void operator()(const int\_var\_decl &x) const

**Definition:** generator.hpp:3862

stan::gm::statement\_visgen::statement\_visgen

statement\_visgen(size\_t indent, bool include\_sampling, bool is\_var, bool is\_fun\_return, std::ostream &o)

**Definition:** generator.hpp:1523

stan::gm::is\_user\_defined\_prob\_function

bool is\_user\_defined\_prob\_function(const std::string &name, const expression &variate, const std::vector< expression > &params)

**Definition:** ast\_def.cpp:1439

stan::gm::constrained\_param\_names\_visgen

**Definition:** generator.hpp:3196

stan::gm::program::statement\_

statement statement\_

**Definition:** ast.hpp:803

stan::gm::generate\_local\_var\_init\_nan\_visgen::operator()

void operator()(const row\_vector\_var\_decl &x) const

**Definition:** generator.hpp:1255

stan::gm::local\_var\_decl\_visgen::indents\_

int indents\_

**Definition:** generator.hpp:997

stan::gm::generate\_init\_vars\_visgen::operator()

void operator()(cholesky\_corr\_var\_decl const &x) const

**Definition:** generator.hpp:1355

stan::gm::validate\_transformed\_params\_visgen::operator()

void operator()(double\_var\_decl const &x) const

**Definition:** generator.hpp:1393

stan::gm::cholesky\_corr\_var\_decl::K\_

expression K\_

**Definition:** ast.hpp:547

stan::gm::write\_csv\_vars\_visgen::operator()

void operator()(const nil &) const

**Definition:** generator.hpp:3696

stan::gm::write\_dims\_visgen::operator()

void operator()(const unit\_vector\_var\_decl &x) const

**Definition:** generator.hpp:2854

stan::gm::DOUBLE\_T

const int DOUBLE\_T

**Definition:** ast.hpp:66

stan::gm::var\_size\_validating\_visgen::operator()

void operator()(nil const &) const

**Definition:** generator.hpp:415

stan::gm::unconstrained\_param\_names\_visgen::operator()

void operator()(const positive\_ordered\_var\_decl &x) const

**Definition:** generator.hpp:3389

stan::gm::arg\_decl::name\_

std::string name\_

**Definition:** ast.hpp:769

stan::gm::row\_vector\_var\_decl

**Definition:** ast.hpp:514

stan::gm::generate\_functions

void generate\_functions(const std::vector< function\_decl\_def > &funs, std::ostream &out)

**Definition:** generator.hpp:4606

stan::gm::write\_dims\_visgen::operator()

void operator()(const row\_vector\_var\_decl &x) const

**Definition:** generator.hpp:2843

stan::gm::INDENT3

const std::string INDENT3(" ")

stan::gm::constrained\_param\_names\_visgen::operator()

void operator()(const cholesky\_corr\_var\_decl &x) const

**Definition:** generator.hpp:3249

stan::gm::validate\_transformed\_params\_visgen::operator()

void operator()(row\_vector\_var\_decl const &x) const

**Definition:** generator.hpp:1422

stan::gm::statement\_visgen::operator()

void operator()(expression const &x) const

**Definition:** generator.hpp:1548

stan::gm::write\_param\_names\_visgen::operator()

void operator()(const cholesky\_corr\_var\_decl &x) const

**Definition:** generator.hpp:2983

stan::gm::assignment::expr\_

expression expr\_

**Definition:** ast.hpp:830

stan::gm::generate\_var\_resizing

void generate\_var\_resizing(const std::vector< var\_decl > &vs, std::ostream &o)

**Definition:** generator.hpp:537

stan::gm::validate\_transformed\_params\_visgen

**Definition:** generator.hpp:1381

stan::gm::generate\_propto\_default\_function

void generate\_propto\_default\_function(const function\_decl\_def &fun, const std::string &scalar\_t\_name, std::ostream &out)

**Definition:** generator.hpp:4522

stan::gm::write\_dims\_visgen::operator()

void operator()(const vector\_var\_decl &x) const

**Definition:** generator.hpp:2838

stan::gm::generate\_set\_param\_ranges

void generate\_set\_param\_ranges(const std::vector< var\_decl > &var\_decls, std::ostream &o)

**Definition:** generator.hpp:2505

stan::gm::generate\_init\_visgen::var\_size\_validator\_

var\_size\_validating\_visgen var\_size\_validator\_

**Definition:** generator.hpp:2561

stan::gm::generate\_init\_vars\_visgen::operator()

void operator()(int\_var\_decl const &x) const

**Definition:** generator.hpp:1315

stan::gm::validate\_transformed\_params\_visgen::operator()

void operator()(nil const &) const

**Definition:** generator.hpp:1388

stan::gm::statements

**Definition:** ast.hpp:185

stan::gm::member\_var\_decl\_visgen::operator()

void operator()(simplex\_var\_decl const &x) const

**Definition:** generator.hpp:939

stan::gm::unconstrained\_param\_names\_visgen

**Definition:** generator.hpp:3347

stan::gm::write\_csv\_visgen::operator()

void operator()(const positive\_ordered\_var\_decl &x) const

**Definition:** generator.hpp:3596

stan::gm::local\_var\_decl\_visgen::generate\_init\_args

void generate\_init\_args(const std::string &type, const std::vector< expression > &ctor\_args, const std::vector< expression > &dims, size\_t dim) const

**Definition:** generator.hpp:1142

stan::gm::set\_param\_ranges\_visgen::operator()

void operator()(const unit\_vector\_var\_decl &x) const

**Definition:** generator.hpp:2368

stan::gm::set\_param\_ranges\_visgen::generate\_increment

void generate\_increment(expression K, std::vector< expression > dims) const

**Definition:** generator.hpp:2480

stan::gm::var\_resizing\_visgen::operator()

void operator()(simplex\_var\_decl const &x) const

**Definition:** generator.hpp:511

stan::gm::ordered\_var\_decl::K\_

expression K\_

**Definition:** ast.hpp:489

stan::gm::unconstrained\_param\_names\_visgen::operator()

void operator()(const unit\_vector\_var\_decl &x) const

**Definition:** generator.hpp:3374

stan::gm::local\_var\_decl\_visgen::is\_fun\_return\_

bool is\_fun\_return\_

**Definition:** generator.hpp:999

stan::is\_var

**Definition:** traits.hpp:421

stan::gm::generate\_init\_visgen::generate\_check\_double

void generate\_check\_double(const std::string &name, size\_t) const

**Definition:** generator.hpp:2784

stan::gm::conditional\_statement

**Definition:** ast.hpp:729

stan::gm::assignment

**Definition:** ast.hpp:828

stan::gm::write\_array\_vars\_visgen

**Definition:** generator.hpp:4010

stan::gm::generate\_init\_vars\_visgen::operator()

void operator()(cov\_matrix\_var\_decl const &x) const

**Definition:** generator.hpp:1359

stan::gm::ordered\_var\_decl

**Definition:** ast.hpp:488

stan::gm::var\_resizing\_visgen::operator()

void operator()(cholesky\_corr\_var\_decl const &x) const

**Definition:** generator.hpp:526

stan::gm::var\_resizing\_visgen::operator()

void operator()(ordered\_var\_decl const &x) const

**Definition:** generator.hpp:514

stan::gm::generate\_validate\_context\_size

void generate\_validate\_context\_size(std::ostream &o, const std::string &stage, const std::string &var\_name, const std::string &base\_type, const std::vector< expression > &dims, const expression &type\_arg1=expression(), const expression &type\_arg2=expression())

**Definition:** generator.hpp:380

stan::gm::var\_size\_validating\_visgen::operator()

void operator()(row\_vector\_var\_decl const &x) const

**Definition:** generator.hpp:425

stan::gm::int\_literal

**Definition:** ast.hpp:297

stan::gm::unconstrained\_param\_names\_visgen::operator()

void operator()(const corr\_matrix\_var\_decl &x) const

**Definition:** generator.hpp:3439

stan::gm::generate\_member\_var\_decls

void generate\_member\_var\_decls(const std::vector< var\_decl > &vs, int indent, std::ostream &o)

**Definition:** generator.hpp:987

stan::gm::generate\_printable

void generate\_printable(const printable &p, std::ostream &o)

**Definition:** generator.hpp:283

stan::gm::range::has\_low

bool has\_low() const

**Definition:** ast\_def.cpp:937

stan::gm::range::high\_

expression high\_

**Definition:** ast.hpp:403

stan::gm::validate\_var\_decl\_visgen::operator()

void operator()(positive\_ordered\_var\_decl const &x) const

**Definition:** generator.hpp:889

stan::gm::var\_size\_validating\_visgen

**Definition:** generator.hpp:409

stan::gm::write\_dims\_visgen::write\_dims\_visgen

write\_dims\_visgen(std::ostream &o)

**Definition:** generator.hpp:2828

stan::gm::write\_param\_names\_visgen::operator()

void operator()(const cholesky\_factor\_var\_decl &x) const

**Definition:** generator.hpp:2980

stan::gm::INDENT

const std::string INDENT(" ")

stan::gm::write\_csv\_vars\_visgen::operator()

void operator()(const unit\_vector\_var\_decl &x) const

**Definition:** generator.hpp:3713

stan::gm::member\_var\_decl\_visgen

**Definition:** generator.hpp:922

stan::gm::generate\_local\_var\_init\_nan

void generate\_local\_var\_init\_nan(const std::vector< var\_decl > &vs, int indent, std::ostream &o, bool is\_var, bool is\_fun\_return)

**Definition:** generator.hpp:1295

stan::gm::local\_var\_decl\_visgen

**Definition:** generator.hpp:996

stan::gm::program::generated\_decl\_

std::pair< std::vector< var\_decl >, std::vector< statement > > generated\_decl\_

**Definition:** ast.hpp:804

stan::gm::write\_csv\_vars\_visgen::write\_array

void write\_array(const std::string &name, const std::vector< expression > &dims) const

**Definition:** generator.hpp:3737

stan::gm::write\_csv\_vars\_visgen::operator()

void operator()(const int\_var\_decl &x) const

**Definition:** generator.hpp:3698

stan::gm::member\_var\_decl\_visgen::operator()

void operator()(cholesky\_factor\_var\_decl const &x) const

**Definition:** generator.hpp:948

stan::gm::local\_var\_decl\_visgen::operator()

void operator()(corr\_matrix\_var\_decl const &x) const

**Definition:** generator.hpp:1113

stan::gm::ROW\_VECTOR\_T

const int ROW\_VECTOR\_T

**Definition:** ast.hpp:68

stan::gm::var\_size\_validating\_visgen::operator()

void operator()(ordered\_var\_decl const &x) const

**Definition:** generator.hpp:434

stan::gm::generate\_init\_vars\_visgen::operator()

void operator()(nil const &) const

**Definition:** generator.hpp:1314

stan::gm::write\_array\_visgen::operator()

void operator()(const corr\_matrix\_var\_decl &x) const

**Definition:** generator.hpp:3945

stan::gm::init\_local\_var\_visgen

**Definition:** generator.hpp:546

stan::gm::program::data\_decl\_

std::vector< var\_decl > data\_decl\_

**Definition:** ast.hpp:797

stan::gm::generate\_dims\_method

void generate\_dims\_method(const program &prog, std::ostream &o)

**Definition:** generator.hpp:2920

stan::gm::generate\_init\_vars\_visgen::generate\_init\_vars\_visgen

generate\_init\_vars\_visgen(int indent, std::ostream &o)

**Definition:** generator.hpp:1309

stan::gm::generate\_local\_var\_init\_nan\_visgen::operator()

void operator()(const cov\_matrix\_var\_decl &x) const

**Definition:** generator.hpp:1279

stan::gm::write\_array\_vars\_visgen::operator()

void operator()(const cholesky\_corr\_var\_decl &x) const

**Definition:** generator.hpp:4064

stan::gm::dump\_member\_var\_visgen::operator()

void operator()(int\_var\_decl const &x) const

**Definition:** generator.hpp:1841

stan::gm::var\_resizing\_visgen::operator()

void operator()(cholesky\_factor\_var\_decl const &x) const

**Definition:** generator.hpp:523

stan::gm::generate\_local\_var\_init\_nan\_visgen::operator()

void operator()(const matrix\_var\_decl &x) const

**Definition:** generator.hpp:1258

stan::gm::function\_decl\_def

**Definition:** ast.hpp:776

stan::gm::constrained\_param\_names\_visgen::operator()

void operator()(const unit\_vector\_var\_decl &x) const

**Definition:** generator.hpp:3223

stan::gm::generate\_include

void generate\_include(const std::string &lib\_name, std::ostream &o)

**Definition:** generator.hpp:323

stan::gm::no\_op\_statement

**Definition:** ast.hpp:763

stan::gm::write\_csv\_visgen::operator()

void operator()(const simplex\_var\_decl &x) const

**Definition:** generator.hpp:3586

stan::gm::write\_csv\_header\_visgen

**Definition:** generator.hpp:3031

stan::gm::generate\_arg\_decl

void generate\_arg\_decl(bool gen\_const, bool gen\_ref, const arg\_decl &decl, const std::string &scalar\_t\_name, std::ostream &out)

**Definition:** generator.hpp:4289

stan::gm::validate\_var\_decl\_visgen::operator()

void operator()(corr\_matrix\_var\_decl const &x) const

**Definition:** generator.hpp:901

stan::gm::write\_csv\_visgen::write\_csv\_visgen

write\_csv\_visgen(std::ostream &o)

**Definition:** generator.hpp:3532

stan::gm::generate\_init\_visgen::generate\_check\_int

void generate\_check\_int(const std::string &name, size\_t) const

**Definition:** generator.hpp:2776

stan::gm::set\_param\_ranges\_visgen::operator()

void operator()(const simplex\_var\_decl &x) const

**Definition:** generator.hpp:2379

stan::gm::generate\_init\_vars\_visgen::operator()

void operator()(ordered\_var\_decl const &x) const

**Definition:** generator.hpp:1343

stan::gm::generate\_constructor

void generate\_constructor(const program &prog, const std::string &model\_name, std::ostream &o)

**Definition:** generator.hpp:2517

stan::gm::generate\_init\_vars\_visgen::operator()

void operator()(simplex\_var\_decl const &x) const

**Definition:** generator.hpp:1339

stan::gm::write\_csv\_header\_visgen::operator()

void operator()(const ordered\_var\_decl &x) const

**Definition:** generator.hpp:3068

stan::gm::init\_local\_var\_visgen::operator()

void operator()(const int\_var\_decl &x) const

**Definition:** generator.hpp:578

stan::gm::cov\_matrix\_var\_decl::K\_

expression K\_

**Definition:** ast.hpp:555

stan::gm::validate\_transformed\_params\_visgen::operator()

void operator()(matrix\_var\_decl const &x) const

**Definition:** generator.hpp:1427

stan::gm::program

**Definition:** ast.hpp:795

stan::gm::write\_array\_visgen::operator()

void operator()(const double\_var\_decl &x) const

**Definition:** generator.hpp:3889

stan::gm::var\_size\_validating\_visgen::operator()

void operator()(cov\_matrix\_var\_decl const &x) const

**Definition:** generator.hpp:449

stan::gm::write\_array\_vars\_visgen::operator()

void operator()(const nil &) const

**Definition:** generator.hpp:4014

stan::gm::write\_dims\_visgen::operator()

void operator()(const int\_var\_decl &x) const

**Definition:** generator.hpp:2832

stan::gm::write\_csv\_vars\_visgen::operator()

void operator()(const matrix\_var\_decl &x) const

**Definition:** generator.hpp:3710

stan::gm::var\_size\_validating\_visgen::stage\_

const std::string stage\_

**Definition:** generator.hpp:410

stan::gm::write\_csv\_visgen::operator()

void operator()(const row\_vector\_var\_decl &x) const

**Definition:** generator.hpp:3570

stan::gm::set\_param\_ranges\_visgen::generate\_increment

void generate\_increment(std::vector< expression > dims) const

**Definition:** generator.hpp:2468

stan::gm::simplex\_var\_decl::K\_

expression K\_

**Definition:** ast.hpp:481

stan::gm::generate\_init\_visgen::generate\_name\_dims

void generate\_name\_dims(const std::string name, size\_t num\_dims) const

**Definition:** generator.hpp:2697

stan::gm::set\_param\_ranges\_visgen::operator()

void operator()(const vector\_var\_decl &x) const

**Definition:** generator.hpp:2359

stan::gm::write\_array\_visgen::operator()

void operator()(const positive\_ordered\_var\_decl &x) const

**Definition:** generator.hpp:3924

stan::gm::generate\_unconstrained\_param\_names\_method

void generate\_unconstrained\_param\_names\_method(const program &prog, std::ostream &o)

**Definition:** generator.hpp:3491

stan::gm::dump\_member\_var\_visgen::operator()

void operator()(positive\_ordered\_var\_decl const &x) const

**Definition:** generator.hpp:2069

stan::gm::statement\_visgen::operator()

void operator()(sample const &x) const

**Definition:** generator.hpp:1553

stan::gm::generate\_function\_name

void generate\_function\_name(const function\_decl\_def &fun, std::ostream &out)

**Definition:** generator.hpp:4412

stan::gm::set\_param\_ranges\_visgen::operator()

void operator()(const matrix\_var\_decl &x) const

**Definition:** generator.hpp:2365

stan::gm::set\_param\_ranges\_visgen

**Definition:** generator.hpp:2328

stan::gm::set\_param\_ranges\_visgen::operator()

void operator()(const row\_vector\_var\_decl &x) const

**Definition:** generator.hpp:2362

stan::gm::write\_csv\_visgen::generate\_initialize\_array\_bounded

void generate\_initialize\_array\_bounded(const D &x, const std::string &base\_type, const std::string &read\_fun\_prefix, const std::vector< expression > &dim\_args) const

**Definition:** generator.hpp:3536

stan::gm::has\_ub

bool has\_ub(const D &x)

**Definition:** generator.hpp:35

stan::gm::cov\_matrix\_var\_decl

**Definition:** ast.hpp:554

stan::gm::write\_param\_names\_visgen::operator()

void operator()(const int\_var\_decl &x) const

**Definition:** generator.hpp:2953

stan::gm::generate\_constrained\_param\_names\_method

void generate\_constrained\_param\_names\_method(const program &prog, std::ostream &o)

**Definition:** generator.hpp:3308

stan::gm::generate\_function

void generate\_function(const function\_decl\_def &fun, std::ostream &out)

Generate the specified function and optionally its default for propto=false for functions ending in \_...

**Definition:** generator.hpp:4543

stan::gm::generate\_function\_template\_parameters

void generate\_function\_template\_parameters(const function\_decl\_def &fun, bool is\_rng, bool is\_lp, bool is\_log, std::ostream &out)

**Definition:** generator.hpp:4356

stan::gm::write\_csv\_header\_visgen::write\_csv\_header\_visgen

write\_csv\_header\_visgen(std::ostream &o)

**Definition:** generator.hpp:3032

stan::gm::generate\_local\_var\_inits

void generate\_local\_var\_inits(std::vector< var\_decl > vs, bool is\_var, bool declare\_vars, std::ostream &o)

**Definition:** generator.hpp:758

stan::gm::write\_array\_vars\_visgen::operator()

void operator()(const positive\_ordered\_var\_decl &x) const

**Definition:** generator.hpp:4053

stan::gm::return\_statement::return\_value\_

expression return\_value\_

**Definition:** ast.hpp:758

stan::gm::generate\_validate\_var\_decls

void generate\_validate\_var\_decls(const std::vector< var\_decl > decls, int indent, std::ostream &o)

**Definition:** generator.hpp:914

stan::gm::generate\_init\_vars\_visgen::operator()

void operator()(matrix\_var\_decl const &x) const

**Definition:** generator.hpp:1331

stan::gm::write\_csv\_visgen::operator()

void operator()(const vector\_var\_decl &x) const

**Definition:** generator.hpp:3565

stan::gm::write\_array\_visgen::operator()

void operator()(const cov\_matrix\_var\_decl &x) const

**Definition:** generator.hpp:3940

stan::gm::statement\_visgen::operator()

void operator()(const reject\_statement &ps) const

**Definition:** generator.hpp:1673

stan::gm::cholesky\_factor\_var\_decl::M\_

expression M\_

**Definition:** ast.hpp:537

stan::gm::generate\_local\_var\_init\_nan\_visgen::generate\_local\_var\_init\_nan\_visgen

generate\_local\_var\_init\_nan\_visgen(bool declare\_vars, bool is\_var, bool is\_fun\_return, int indent, std::ostream &o)

**Definition:** generator.hpp:1232

stan::gm::write\_array\_visgen::operator()

void operator()(const unit\_vector\_var\_decl &x) const

**Definition:** generator.hpp:3909

stan::gm::generate\_destructor

void generate\_destructor(const std::string &model\_name, std::ostream &o)

**Definition:** generator.hpp:2320

stan::gm::generate\_indent

void generate\_indent(size\_t indent, std::ostream &o)

**Definition:** generator.hpp:50

stan::gm::var\_size\_validating\_visgen::operator()

void operator()(corr\_matrix\_var\_decl const &x) const

**Definition:** generator.hpp:452

stan::gm::write\_dims\_visgen::operator()

void operator()(const positive\_ordered\_var\_decl &x) const

**Definition:** generator.hpp:2869

stan::gm::write\_csv\_header\_visgen::operator()

void operator()(const nil &) const

**Definition:** generator.hpp:3035

stan::gm::dump\_member\_var\_visgen::operator()

void operator()(double\_var\_decl const &x) const

**Definition:** generator.hpp:1871

stan::gm::write\_array\_visgen::generate\_initialize\_array

void generate\_initialize\_array(const std::string &var\_type, const std::string &read\_type, const std::vector< expression > &read\_args, const std::string &name, const std::vector< expression > &dims) const

**Definition:** generator.hpp:3950

stan::gm::var\_size\_validating\_visgen::var\_size\_validating\_visgen

var\_size\_validating\_visgen(std::ostream &o, const std::string &stage)

**Definition:** generator.hpp:411

stan::gm::dump\_member\_var\_visgen::dump\_member\_var\_visgen

dump\_member\_var\_visgen(std::ostream &o)

**Definition:** generator.hpp:1835

stan::gm::init\_local\_var\_visgen::operator()

void operator()(const matrix\_var\_decl &x) const

**Definition:** generator.hpp:595

stan::gm::write\_csv\_vars\_visgen::operator()

void operator()(const positive\_ordered\_var\_decl &x) const

**Definition:** generator.hpp:3722

stan::gm::for\_statement

**Definition:** ast.hpp:718

stan::gm::generate\_validate\_var\_decl

void generate\_validate\_var\_decl(const var\_decl &decl, int indent, std::ostream &o)

**Definition:** generator.hpp:907

stan::gm::generate\_init\_vars\_visgen::operator()

void operator()(vector\_var\_decl const &x) const

**Definition:** generator.hpp:1323

stan::gm::to\_string

std::string to\_string(T i)

**Definition:** generator.hpp:44

stan::gm::write\_csv\_header\_visgen::operator()

void operator()(const positive\_ordered\_var\_decl &x) const

**Definition:** generator.hpp:3073

stan::gm::write\_dims\_visgen::generate\_dims\_array

void generate\_dims\_array(const std::vector< expression > &matrix\_dims\_exprs, const std::vector< expression > &array\_dims\_exprs) const

**Definition:** generator.hpp:2899

stan::gm::member\_var\_decl\_visgen::operator()

void operator()(positive\_ordered\_var\_decl const &x) const

**Definition:** generator.hpp:945

stan::gm::unconstrained\_param\_names\_visgen::generate\_param\_names\_array

void generate\_param\_names\_array(const std::vector< expression > &matrix\_dims, const std::string &name, const std::vector< expression > &dims) const

**Definition:** generator.hpp:3452

stan::gm::unconstrained\_param\_names\_visgen::operator()

void operator()(const double\_var\_decl &x) const

**Definition:** generator.hpp:3355

stan::gm::write\_csv\_header\_visgen::operator()

void operator()(const simplex\_var\_decl &x) const

**Definition:** generator.hpp:3063

stan::gm::write\_array\_visgen::operator()

void operator()(const simplex\_var\_decl &x) const

**Definition:** generator.hpp:3914

stan::gm::vector\_var\_decl::M\_

expression M\_

**Definition:** ast.hpp:506

stan::gm::increment\_log\_prob\_statement

**Definition:** ast.hpp:712

stan::gm::generate\_bare\_type

void generate\_bare\_type(const expr\_type &t, const std::string &scalar\_t\_name, std::ostream &out)

**Definition:** generator.hpp:4241

stan::gm::dump\_member\_var\_visgen::var\_resizer\_

var\_resizing\_visgen var\_resizer\_

**Definition:** generator.hpp:1833

stan::gm::printable

**Definition:** ast.hpp:256

stan::gm::init\_local\_var\_visgen::init\_local\_var\_visgen

init\_local\_var\_visgen(bool declare\_vars, bool is\_var, std::ostream &o)

**Definition:** generator.hpp:549

stan::gm::generate\_model\_typedef

void generate\_model\_typedef(const std::string &model\_name, std::ostream &out)

**Definition:** generator.hpp:4235

stan::gm::member\_var\_decl\_visgen::operator()

void operator()(unit\_vector\_var\_decl const &x) const

**Definition:** generator.hpp:936

stan::is\_vector

**Definition:** traits.hpp:83

stan::gm::init\_local\_var\_visgen::operator()

void operator()(const cholesky\_factor\_var\_decl &x) const

**Definition:** generator.hpp:621

stan::gm::init\_local\_var\_visgen::operator()

void operator()(const cholesky\_corr\_var\_decl &x) const

**Definition:** generator.hpp:628

stan::gm::print\_statement

**Definition:** ast.hpp:745

stan::gm::generate\_functor\_arguments

void generate\_functor\_arguments(const function\_decl\_def &fun, bool is\_rng, bool is\_lp, bool is\_log, std::ostream &out)

**Definition:** generator.hpp:4447

stan::gm::write\_array\_vars\_visgen::operator()

void operator()(const matrix\_var\_decl &x) const

**Definition:** generator.hpp:4032

stan::gm::write\_array\_vars\_visgen::operator()

void operator()(const int\_var\_decl &x) const

**Definition:** generator.hpp:4016

stan::gm::statement\_visgen::operator()

void operator()(const increment\_log\_prob\_statement &x) const

**Definition:** generator.hpp:1632

stan::gm::cholesky\_factor\_var\_decl::N\_

expression N\_

**Definition:** ast.hpp:538

stan::gm::write\_array\_vars\_visgen::operator()

void operator()(const cov\_matrix\_var\_decl &x) const

**Definition:** generator.hpp:4070

stan::gm::write\_csv\_header\_visgen::operator()

void operator()(const cholesky\_corr\_var\_decl &x) const

**Definition:** generator.hpp:3084

stan::gm::sample::expr\_

expression expr\_

**Definition:** ast.hpp:819

stan::gm::generate\_indexed\_expr

void generate\_indexed\_expr(const std::string &expr, const std::vector< expression > indexes, base\_expr\_type base\_type, size\_t e\_num\_dims, std::ostream &o)

**Definition:** generator.hpp:87

stan::gm::dump\_member\_var\_visgen

**Definition:** generator.hpp:1832

stan::gm::generate\_function\_inline\_return\_type

void generate\_function\_inline\_return\_type(const function\_decl\_def &fun, const std::string &scalar\_t\_name, std::ostream &out)

**Definition:** generator.hpp:4404

stan::gm::local\_var\_decl\_visgen::operator()

void operator()(unit\_vector\_var\_decl const &x) const

**Definition:** generator.hpp:1050

stan::gm::base\_var\_decl::base\_type\_

base\_expr\_type base\_type\_

**Definition:** ast.hpp:431

stan::gm::set\_param\_ranges\_visgen::operator()

void operator()(const cov\_matrix\_var\_decl &x) const

**Definition:** generator.hpp:2428

stan::gm::init\_local\_var\_visgen::declare\_vars\_

const bool declare\_vars\_

**Definition:** generator.hpp:547

stan::gm::constrained\_param\_names\_visgen::operator()

void operator()(const cov\_matrix\_var\_decl &x) const

**Definition:** generator.hpp:3255

stan::gm::expression::expr\_

expression\_t expr\_

**Definition:** ast.hpp:252

stan::gm::while\_statement::body\_

statement body\_

**Definition:** ast.hpp:739

stan::gm::generate\_init\_visgen::operator()

void operator()(simplex\_var\_decl const &x) const

**Definition:** generator.hpp:2638

stan::gm::init\_local\_var\_visgen::operator()

void operator()(const cov\_matrix\_var\_decl &x) const

**Definition:** generator.hpp:637

stan::gm::generate\_init\_vars\_visgen::operator()

void operator()(positive\_ordered\_var\_decl const &x) const

**Definition:** generator.hpp:1347

stan::gm::program::parameter\_decl\_

std::vector< var\_decl > parameter\_decl\_

**Definition:** ast.hpp:800

stan::gm::var\_decl

**Definition:** ast.hpp:592

stan::gm::needs\_template\_params

bool needs\_template\_params(const function\_decl\_def &fun)

**Definition:** generator.hpp:4346

stan::gm::write\_array\_visgen

**Definition:** generator.hpp:3857

stan::gm::var\_size\_validating\_visgen::operator()

void operator()(simplex\_var\_decl const &x) const

**Definition:** generator.hpp:431

stan::gm::expr\_type::num\_dims\_

size\_t num\_dims\_

**Definition:** ast.hpp:76

stan::gm::generate\_function\_body

void generate\_function\_body(const function\_decl\_def &fun, const std::string &scalar\_t\_name, std::ostream &out)

**Definition:** generator.hpp:4473

stan::gm::local\_var\_decl\_visgen::operator()

void operator()(positive\_ordered\_var\_decl const &x) const

**Definition:** generator.hpp:1074

stan::gm::var\_size\_validating\_visgen::operator()

void operator()(matrix\_var\_decl const &x) const

**Definition:** generator.hpp:440

stan::gm::write\_array\_vars\_visgen::write\_array\_vars\_visgen

write\_array\_vars\_visgen(std::ostream &o)

**Definition:** generator.hpp:4011

stan::gm::var\_resizing\_visgen

**Definition:** generator.hpp:491

stan::gm::printable\_visgen::operator()

void operator()(const std::string &s) const

**Definition:** generator.hpp:274

stan::gm::write\_array\_visgen::operator()

void operator()(const vector\_var\_decl &x) const

**Definition:** generator.hpp:3893

stan::gm::write\_csv\_header\_visgen::operator()

void operator()(const corr\_matrix\_var\_decl &x) const

**Definition:** generator.hpp:3096

stan::gm::write\_param\_names\_visgen::operator()

void operator()(const double\_var\_decl &x) const

**Definition:** generator.hpp:2956

stan::gm::write\_csv\_visgen::operator()

void operator()(const ordered\_var\_decl &x) const

**Definition:** generator.hpp:3591

stan::gm::init\_local\_var\_visgen::generate\_initialize\_array

void generate\_initialize\_array(const std::string &var\_type, const std::string &read\_type, const std::vector< expression > &read\_args, const std::string &name, const std::vector< expression > &dims) const

**Definition:** generator.hpp:649

stan::gm::generate\_propto\_default\_function\_body

void generate\_propto\_default\_function\_body(const function\_decl\_def &fun, std::ostream &out)

**Definition:** generator.hpp:4505

stan::gm::write\_param\_names\_visgen::operator()

void operator()(const cov\_matrix\_var\_decl &x) const

**Definition:** generator.hpp:2986

stan::gm::conditional\_statement::bodies\_

std::vector< statement > bodies\_

**Definition:** ast.hpp:731

stan::gm::generate\_init\_vars\_visgen::operator()

void operator()(row\_vector\_var\_decl const &x) const

**Definition:** generator.hpp:1327

stan::gm::init\_local\_var\_visgen::operator()

void operator()(const corr\_matrix\_var\_decl &x) const

**Definition:** generator.hpp:643

stan::gm::while\_statement::condition\_

expression condition\_

**Definition:** ast.hpp:738

stan::gm::write\_csv\_header\_visgen::operator()

void operator()(const cov\_matrix\_var\_decl &x) const

**Definition:** generator.hpp:3090

stan::gm::cholesky\_factor\_var\_decl

**Definition:** ast.hpp:536

stan::gm::unconstrained\_param\_names\_visgen::unconstrained\_param\_names\_visgen

unconstrained\_param\_names\_visgen(std::ostream &o)

**Definition:** generator.hpp:3348

stan::gm::positive\_ordered\_var\_decl

**Definition:** ast.hpp:496

stan::gm::var\_size\_validating\_visgen::operator()

void operator()(double\_var\_decl const &x) const

**Definition:** generator.hpp:419

stan::gm::init\_local\_var\_visgen::operator()

void operator()(const vector\_var\_decl &x) const

**Definition:** generator.hpp:585

stan::gm::write\_csv\_visgen

**Definition:** generator.hpp:3531

stan::gm::validate\_transformed\_params\_visgen::indents\_

int indents\_

**Definition:** generator.hpp:1382

stan::gm::write\_param\_names\_visgen::operator()

void operator()(const ordered\_var\_decl &x) const

**Definition:** generator.hpp:2974

stan::gm::int\_var\_decl::range\_

range range\_

**Definition:** ast.hpp:456

stan::gm::generate\_init\_visgen::generate\_indent\_num\_dims

void generate\_indent\_num\_dims(size\_t base\_indent, const std::vector< expression > &dims, const expression &dim1, const expression &dim2) const

**Definition:** generator.hpp:2714

stan::gm::set\_param\_ranges\_visgen::operator()

void operator()(const double\_var\_decl &x) const

**Definition:** generator.hpp:2356

stan::gm::matrix\_var\_decl::N\_

expression N\_

**Definition:** ast.hpp:527

stan::gm::generate\_init\_visgen::generate\_write\_loop

void generate\_write\_loop(const std::string &write\_method\_name, const std::string &var\_name, const std::vector< expression > &dims) const

**Definition:** generator.hpp:2687

stan::gm::generate\_init\_visgen::operator()

void operator()(int\_var\_decl const &x) const

**Definition:** generator.hpp:2567

stan::gm::write\_array\_visgen::write\_array\_visgen

write\_array\_visgen(std::ostream &o)

**Definition:** generator.hpp:3858

stan::gm::init\_local\_var\_visgen::operator()

void operator()(const simplex\_var\_decl &x) const

**Definition:** generator.hpp:606

stan::gm::write\_csv\_vars\_visgen::operator()

void operator()(const cholesky\_corr\_var\_decl &x) const

**Definition:** generator.hpp:3728

stan::gm::write\_csv\_vars\_visgen

**Definition:** generator.hpp:3692

stan::gm::generate\_init\_visgen::operator()

void operator()(cholesky\_factor\_var\_decl const &x) const

**Definition:** generator.hpp:2659

stan::gm::type\_name

**Definition:** singleton\_argument.hpp:13

stan::gm::has\_only\_int\_args

bool has\_only\_int\_args(const function\_decl\_def &fun)

**Definition:** generator.hpp:4302

stan::gm::write\_dims\_visgen::operator()

void operator()(const cov\_matrix\_var\_decl &x) const

**Definition:** generator.hpp:2886

stan::gm::var\_size\_validating\_visgen::operator()

void operator()(unit\_vector\_var\_decl const &x) const

**Definition:** generator.hpp:428

stan::gm::generate\_typedef

void generate\_typedef(const std::string &type, const std::string &abbrev, std::ostream &o)

**Definition:** generator.hpp:310

stan::gm::write\_csv\_vars\_visgen::operator()

void operator()(const ordered\_var\_decl &x) const

**Definition:** generator.hpp:3719

stan::gm::dump\_member\_var\_visgen::operator()

void operator()(cov\_matrix\_var\_decl const &x) const

**Definition:** generator.hpp:2267

stan::gm::generate\_init\_visgen::operator()

void operator()(row\_vector\_var\_decl const &x) const

**Definition:** generator.hpp:2615

stan::gm::unconstrained\_param\_names\_visgen::operator()

void operator()(const nil &) const

**Definition:** generator.hpp:3351

stan::MINOR\_VERSION

const std::string MINOR\_VERSION

Minor version number for Stan package.

**Definition:** version.hpp:12

stan::gm::validate\_var\_decl\_visgen::operator()

void operator()(double\_var\_decl const &x) const

**Definition:** generator.hpp:856

stan::gm::validate\_var\_decl\_visgen::operator()

void operator()(vector\_var\_decl const &x) const

**Definition:** generator.hpp:859

stan::gm::VECTOR\_T

const int VECTOR\_T

**Definition:** ast.hpp:67

stan::gm::program::derived\_decl\_

std::pair< std::vector< var\_decl >, std::vector< statement > > derived\_decl\_

**Definition:** ast.hpp:802

stan::gm::visgen::o\_

std::ostream & o\_

**Definition:** generator.hpp:66

stan::gm::var\_resizing\_visgen::operator()

void operator()(cov\_matrix\_var\_decl const &x) const

**Definition:** generator.hpp:529

stan::gm::write\_csv\_header\_visgen::operator()

void operator()(const matrix\_var\_decl &x) const

**Definition:** generator.hpp:3052

stan::gm::MATRIX\_T

const int MATRIX\_T

**Definition:** ast.hpp:69

stan::gm::generate\_end\_namespace

void generate\_end\_namespace(std::ostream &o)

**Definition:** generator.hpp:75

stan::gm::validate\_transformed\_params\_visgen::operator()

void operator()(cov\_matrix\_var\_decl const &x) const

**Definition:** generator.hpp:1445

stan::gm::generate\_class\_decl

void generate\_class\_decl(const std::string &model\_name, std::ostream &o)

**Definition:** generator.hpp:339

stan::gm::generate\_local\_var\_init\_nan\_visgen::operator()

void operator()(const cholesky\_corr\_var\_decl &x) const

**Definition:** generator.hpp:1276

stan::math::dims

void dims(const T &x, std::vector< int > &result)

**Definition:** dims.hpp:13

stan::gm::init\_local\_var\_visgen::operator()

void operator()(const row\_vector\_var\_decl &x) const

**Definition:** generator.hpp:590

stan::gm::generate\_includes

void generate\_includes(std::ostream &o)

**Definition:** generator.hpp:327

stan::gm::var\_resizing\_visgen::operator()

void operator()(unit\_vector\_var\_decl const &x) const

**Definition:** generator.hpp:508

stan::gm::generate\_local\_var\_init\_nan\_visgen::declare\_vars\_

const bool declare\_vars\_

**Definition:** generator.hpp:1228

stan::gm::local\_var\_decl\_visgen::operator()

void operator()(cov\_matrix\_var\_decl const &x) const

**Definition:** generator.hpp:1102

stan::gm::range::low\_

expression low\_

**Definition:** ast.hpp:402

stan::gm::write\_dims\_visgen::operator()

void operator()(const simplex\_var\_decl &x) const

**Definition:** generator.hpp:2859

stan::gm::generate\_cpp

void generate\_cpp(const program &prog, const std::string &model\_name, std::ostream &out)

**Definition:** generator.hpp:4614

stan::gm::generate\_init\_visgen::operator()

void operator()(matrix\_var\_decl const &x) const

**Definition:** generator.hpp:2623

stan::gm::write\_csv\_vars\_visgen::operator()

void operator()(const cov\_matrix\_var\_decl &x) const

**Definition:** generator.hpp:3731

stan::gm::expr\_type

**Definition:** ast.hpp:74

stan::gm::unconstrained\_param\_names\_visgen::operator()

void operator()(const simplex\_var\_decl &x) const

**Definition:** generator.hpp:3379

stan::gm::dump\_member\_var\_visgen::operator()

void operator()(unit\_vector\_var\_decl const &x) const

**Definition:** generator.hpp:1967

stan::gm::printable::printable\_

printable\_t printable\_

**Definition:** ast.hpp:267

stan::gm::write\_csv\_header\_visgen::operator()

void operator()(const row\_vector\_var\_decl &x) const

**Definition:** generator.hpp:3047

stan::gm::variable\_dims::dims\_

std::vector< expression > dims\_

**Definition:** ast.hpp:290

stan::gm::write\_csv\_header\_visgen::generate\_csv\_header\_array

void generate\_csv\_header\_array(const std::vector< expression > &matrix\_dims, const std::string &name, const std::vector< expression > &dims) const

**Definition:** generator.hpp:3103

stan::gm::dump\_member\_var\_visgen::operator()

void operator()(vector\_var\_decl const &x) const

**Definition:** generator.hpp:1899

stan::gm::constrained\_param\_names\_visgen::operator()

void operator()(const vector\_var\_decl &x) const

**Definition:** generator.hpp:3207

stan::gm::set\_param\_ranges\_visgen::operator()

void operator()(const ordered\_var\_decl &x) const

**Definition:** generator.hpp:2390

stan::gm::constrained\_param\_names\_visgen::operator()

void operator()(const positive\_ordered\_var\_decl &x) const

**Definition:** generator.hpp:3238

stan::gm::generate\_typedefs

void generate\_typedefs(std::ostream &o)

**Definition:** generator.hpp:316

stan::gm::unit\_vector\_var\_decl::K\_

expression K\_

**Definition:** ast.hpp:473

stan::gm::unconstrained\_param\_names\_visgen::operator()

void operator()(const matrix\_var\_decl &x) const

**Definition:** generator.hpp:3368

stan::gm::write\_param\_names\_visgen::operator()

void operator()(const unit\_vector\_var\_decl &x) const

**Definition:** generator.hpp:2968

stan::gm::generate\_local\_var\_init\_nan\_visgen::generate\_init

void generate\_init(const T &x) const

**Definition:** generator.hpp:1286

stan::gm::set\_param\_ranges\_visgen::operator()

void operator()(const int\_var\_decl &x) const

**Definition:** generator.hpp:2333

stan::gm::generate\_local\_var\_decls

void generate\_local\_var\_decls(const std::vector< var\_decl > &vs, int indent, std::ostream &o, bool is\_var, bool is\_fun\_return)

**Definition:** generator.hpp:1215

stan::gm::for\_statement::statement\_

statement statement\_

**Definition:** ast.hpp:721

stan::gm::write\_dims\_visgen::operator()

void operator()(const cholesky\_factor\_var\_decl &x) const

**Definition:** generator.hpp:2874

stan::gm::local\_var\_decl\_visgen::is\_var\_

bool is\_var\_

**Definition:** generator.hpp:998

stan::gm::generate\_function\_arguments

void generate\_function\_arguments(const function\_decl\_def &fun, bool is\_rng, bool is\_lp, bool is\_log, std::ostream &out)

**Definition:** generator.hpp:4418

stan::gm::generate\_init\_visgen::function\_args

std::string function\_args(const std::string &fun\_prefix, const D &x) const

**Definition:** generator.hpp:2575

stan::gm::variable\_dims::name\_

std::string name\_

**Definition:** ast.hpp:289

stan::gm::validate\_transformed\_params\_visgen::validate\_array

void validate\_array(const std::string &name, const std::vector< expression > &dims, size\_t matrix\_dims) const

**Definition:** generator.hpp:1457

stan::gm::unconstrained\_param\_names\_visgen::operator()

void operator()(const row\_vector\_var\_decl &x) const

**Definition:** generator.hpp:3363

stan::gm::generate\_local\_var\_init\_nan\_visgen::operator()

void operator()(const simplex\_var\_decl &x) const

**Definition:** generator.hpp:1264

stan::gm::write\_dims\_visgen::operator()

void operator()(const cholesky\_corr\_var\_decl &x) const

**Definition:** generator.hpp:2880

stan::gm::for\_statement::range\_

range range\_

**Definition:** ast.hpp:720

stan::gm::generate\_init\_visgen

**Definition:** generator.hpp:2560

stan::gm::member\_var\_decl\_visgen::operator()

void operator()(ordered\_var\_decl const &x) const

**Definition:** generator.hpp:942

stan::gm::validate\_var\_decl\_visgen::validate\_var\_decl\_visgen

validate\_var\_decl\_visgen(int indents, std::ostream &o)

**Definition:** generator.hpp:785

stan::gm::matrix\_var\_decl

**Definition:** ast.hpp:524

stan::gm::visgen::result\_type

void result\_type

**Definition:** generator.hpp:65

stan::gm::var\_resizing\_visgen::operator()

void operator()(row\_vector\_var\_decl const &x) const

**Definition:** generator.hpp:505

stan::gm::generate\_version\_comment

void generate\_version\_comment(std::ostream &o)

**Definition:** generator.hpp:334

stan::gm::var\_resizing\_visgen::operator()

void operator()(positive\_ordered\_var\_decl const &x) const

**Definition:** generator.hpp:517

stan::gm::var\_size\_validating\_visgen::operator()

void operator()(vector\_var\_decl const &x) const

**Definition:** generator.hpp:422

stan::gm::var\_resizing\_visgen::operator()

void operator()(matrix\_var\_decl const &x) const

**Definition:** generator.hpp:520

stan::gm::write\_csv\_header\_visgen::operator()

void operator()(const double\_var\_decl &x) const

**Definition:** generator.hpp:3039

stan::gm::member\_var\_decl\_visgen::operator()

void operator()(corr\_matrix\_var\_decl const &x) const

**Definition:** generator.hpp:957

stan::gm::generate\_local\_var\_init\_nan\_visgen::operator()

void operator()(const cholesky\_factor\_var\_decl &x) const

**Definition:** generator.hpp:1273

stan::gm::local\_var\_decl\_visgen::operator()

void operator()(simplex\_var\_decl const &x) const

**Definition:** generator.hpp:1058

stan::gm::write\_param\_names\_visgen::operator()

void operator()(const row\_vector\_var\_decl &x) const

**Definition:** generator.hpp:2962

stan::gm::generate\_init\_visgen::operator()

void operator()(positive\_ordered\_var\_decl const &x) const

**Definition:** generator.hpp:2652

stan::gm::write\_csv\_visgen::generate\_initialize\_array

void generate\_initialize\_array(const std::string &var\_type, const std::string &read\_type, const std::vector< expression > &read\_args, const std::string &name, const std::vector< expression > &dims) const

**Definition:** generator.hpp:3622

stan::gm::validate\_var\_decl\_visgen::operator()

void operator()(ordered\_var\_decl const &x) const

**Definition:** generator.hpp:886

stan::gm::validate\_transformed\_params\_visgen::validate\_transformed\_params\_visgen

validate\_transformed\_params\_visgen(int indents, std::ostream &o)

**Definition:** generator.hpp:1383

stan::gm::expression

**Definition:** ast.hpp:216

stan::gm::unconstrained\_param\_names\_visgen::operator()

void operator()(const int\_var\_decl &x) const

**Definition:** generator.hpp:3352

stan::gm::print\_quoted\_expression

static void print\_quoted\_expression(std::ostream &o, const expression &e)

**Definition:** generator.hpp:265

stan::gm::function\_decl\_def::arg\_decls\_

std::vector< arg\_decl > arg\_decls\_

**Definition:** ast.hpp:784

stan::gm::write\_param\_names\_visgen::write\_param\_names\_visgen

write\_param\_names\_visgen(std::ostream &o)

**Definition:** generator.hpp:2949

stan::gm::generate\_init\_vars\_visgen::operator()

void operator()(corr\_matrix\_var\_decl const &x) const

**Definition:** generator.hpp:1363

stan::gm::generate\_init\_visgen::operator()

void operator()(vector\_var\_decl const &x) const

**Definition:** generator.hpp:2607

stan::gm::statement\_visgen::operator()

void operator()(const while\_statement &x) const

**Definition:** generator.hpp:1708

stan::gm::local\_var\_decl\_visgen::operator()

void operator()(const cholesky\_corr\_var\_decl &x) const

**Definition:** generator.hpp:1093

stan::gm::validate\_transformed\_params\_visgen::operator()

void operator()(cholesky\_factor\_var\_decl const &x) const

**Definition:** generator.hpp:1433

stan::gm::var\_resizing\_visgen::operator()

void operator()(int\_var\_decl const &x) const

**Definition:** generator.hpp:496

stan::gm::base\_var\_decl::name\_

std::string name\_

**Definition:** ast.hpp:429

stan::gm::var\_resizing\_visgen::var\_resizing\_visgen

var\_resizing\_visgen(std::ostream &o)

**Definition:** generator.hpp:492

stan::gm::local\_var\_decl\_visgen::operator()

void operator()(nil const &) const

**Definition:** generator.hpp:1009

stan::gm::write\_csv\_vars\_visgen::operator()

void operator()(const corr\_matrix\_var\_decl &x) const

**Definition:** generator.hpp:3734

stan::gm::var\_size\_validating\_visgen::operator()

void operator()(positive\_ordered\_var\_decl const &x) const

**Definition:** generator.hpp:437

stan::gm::validate\_var\_decl\_visgen::operator()

void operator()(cholesky\_factor\_var\_decl const &x) const

**Definition:** generator.hpp:892

stan::gm::member\_var\_decl\_visgen::operator()

void operator()(vector\_var\_decl const &x) const

**Definition:** generator.hpp:960

stan::gm::validate\_transformed\_params\_visgen::operator()

void operator()(unit\_vector\_var\_decl const &x) const

**Definition:** generator.hpp:1402

stan::gm::generate\_local\_var\_init\_nan\_visgen::indent\_

const int indent\_

**Definition:** generator.hpp:1231

stan::gm::simplex\_var\_decl

**Definition:** ast.hpp:480

stan::gm::set\_param\_ranges\_visgen::set\_param\_ranges\_visgen

set\_param\_ranges\_visgen(std::ostream &o)

**Definition:** generator.hpp:2329

stan::gm::generate\_statement

void generate\_statement(statement const &s, int indent, std::ostream &o, bool include\_sampling, bool is\_var, bool is\_fun\_return)

**Definition:** generator.hpp:1746

stan::gm::local\_var\_decl\_visgen::operator()

void operator()(matrix\_var\_decl const &x) const

**Definition:** generator.hpp:1039

stan::gm::function\_decl\_def::name\_

std::string name\_

**Definition:** ast.hpp:783

stan::gm::write\_csv\_vars\_visgen::write\_csv\_vars\_visgen

write\_csv\_vars\_visgen(std::ostream &o)

**Definition:** generator.hpp:3693

stan::gm::validate\_transformed\_params\_visgen::operator()

void operator()(ordered\_var\_decl const &x) const

**Definition:** generator.hpp:1412

stan::gm::set\_param\_ranges\_visgen::generate\_increment\_i

void generate\_increment\_i(std::vector< expression > dims) const

**Definition:** generator.hpp:2456

stan::gm::write\_csv\_visgen::operator()

void operator()(const nil &) const

**Definition:** generator.hpp:3556

stan::gm::write\_array\_vars\_visgen::operator()

void operator()(const vector\_var\_decl &x) const

**Definition:** generator.hpp:4022

stan::gm::dump\_member\_var\_visgen::operator()

void operator()(cholesky\_corr\_var\_decl const &x) const

**Definition:** generator.hpp:2222

stan::gm::sample

**Definition:** ast.hpp:818

stan::gm::write\_param\_names\_visgen::operator()

void operator()(const positive\_ordered\_var\_decl &x) const

**Definition:** generator.hpp:2977

stan::gm::dump\_member\_var\_visgen::operator()

void operator()(simplex\_var\_decl const &x) const

**Definition:** generator.hpp:2001

stan::gm::dump\_member\_var\_visgen::operator()

void operator()(cholesky\_factor\_var\_decl const &x) const

**Definition:** generator.hpp:2177

stan::gm::unconstrained\_param\_names\_visgen::operator()

void operator()(const cholesky\_factor\_var\_decl &x) const

**Definition:** generator.hpp:3394

stan::gm::generate\_param\_names\_method

void generate\_param\_names\_method(const program &prog, std::ostream &o)

**Definition:** generator.hpp:3001

stan::gm::cholesky\_corr\_var\_decl

**Definition:** ast.hpp:546

stan::gm::generate\_init\_visgen::operator()

void operator()(corr\_matrix\_var\_decl const &x) const

**Definition:** generator.hpp:2680

stan::gm::generate\_init\_vars\_visgen::operator()

void operator()(cholesky\_factor\_var\_decl const &x) const

**Definition:** generator.hpp:1351

stan::gm::write\_csv\_visgen::operator()

void operator()(const int\_var\_decl &x) const

**Definition:** generator.hpp:3557

stan::gm::reject\_statement

**Definition:** ast.hpp:751

stan::gm::local\_var\_decl\_visgen::operator()

void operator()(double\_var\_decl const &x) const

**Definition:** generator.hpp:1014

stan::gm::write\_csv\_header\_visgen::operator()

void operator()(const unit\_vector\_var\_decl &x) const

**Definition:** generator.hpp:3058

stan::gm::validate\_var\_decl\_visgen::operator()

void operator()(int\_var\_decl const &x) const

**Definition:** generator.hpp:853

stan::gm::dump\_member\_var\_visgen::operator()

void operator()(matrix\_var\_decl const &x) const

**Definition:** generator.hpp:2103

stan::gm::dump\_member\_var\_visgen::operator()

void operator()(ordered\_var\_decl const &x) const

**Definition:** generator.hpp:2035

stan::gm::dump\_member\_var\_visgen::operator()

void operator()(corr\_matrix\_var\_decl const &x) const

**Definition:** generator.hpp:2141

stan::gm::statement\_visgen

**Definition:** generator.hpp:1518

stan::gm::generate\_type

void generate\_type(const std::string &base\_type, const std::vector< expression > &, size\_t end, std::ostream &o)

**Definition:** generator.hpp:126

stan::gm::write\_dims\_visgen::operator()

void operator()(const matrix\_var\_decl &x) const

**Definition:** generator.hpp:2848

stan::gm::validate\_transformed\_params\_visgen::operator()

void operator()(simplex\_var\_decl const &x) const

**Definition:** generator.hpp:1407

stan::gm::write\_csv\_visgen::operator()

void operator()(const corr\_matrix\_var\_decl &x) const

**Definition:** generator.hpp:3617

stan::gm::generate\_using\_namespace

void generate\_using\_namespace(const std::string &ns, std::ostream &o)

**Definition:** generator.hpp:292

stan::gm::statement\_visgen::include\_sampling\_

bool include\_sampling\_

**Definition:** generator.hpp:1520

stan::gm::write\_dims\_visgen::operator()

void operator()(const nil &) const

**Definition:** generator.hpp:2831

stan::gm::dump\_member\_var\_visgen::var\_size\_validator\_

var\_size\_validating\_visgen var\_size\_validator\_

**Definition:** generator.hpp:1834

stan::gm::constrained\_param\_names\_visgen::operator()

void operator()(const int\_var\_decl &x) const

**Definition:** generator.hpp:3201

stan::gm::generate\_init\_visgen::generate\_declaration

void generate\_declaration(const std::string &name, const std::string &base\_type, const std::vector< expression > &dims, const expression &type\_arg1=expression(), const expression &type\_arg2=expression()) const

**Definition:** generator.hpp:2703

stan::gm::generate\_log\_prob

void generate\_log\_prob(program const &p, std::ostream &o)

**Definition:** generator.hpp:1768

stan::gm::generate\_member\_var\_inits

void generate\_member\_var\_inits(const std::vector< var\_decl > &vs, std::ostream &o)

**Definition:** generator.hpp:2313

stan::gm::int\_var\_decl

**Definition:** ast.hpp:455

stan::gm::EOL2

const std::string EOL2("\n\n")

stan::gm::local\_var\_decl\_visgen::operator()

void operator()(row\_vector\_var\_decl const &x) const

**Definition:** generator.hpp:1029

stan::gm::member\_var\_decl\_visgen::operator()

void operator()(nil const &) const

**Definition:** generator.hpp:929

stan::gm::member\_var\_decl\_visgen::operator()

void operator()(int\_var\_decl const &x) const

**Definition:** generator.hpp:930

stan::gm::generate\_start\_namespace

void generate\_start\_namespace(std::string name, std::ostream &o)

**Definition:** generator.hpp:70

stan::gm::init\_local\_var\_visgen::generate\_initialize\_array\_bounded

void generate\_initialize\_array\_bounded(const D &x, const std::string &base\_type, const std::string &read\_fun\_prefix, const std::vector< expression > &dim\_args) const

**Definition:** generator.hpp:557

stan::gm::write\_array\_vars\_visgen::operator()

void operator()(const cholesky\_factor\_var\_decl &x) const

**Definition:** generator.hpp:4058

stan::gm::member\_var\_decl\_visgen::declare\_array

void declare\_array(std::string const &type, std::string const &name, size\_t size) const

**Definition:** generator.hpp:969

stan::gm::validate\_var\_decl\_visgen::basic\_validate

void basic\_validate(T const &x) const

**Definition:** generator.hpp:817

ast.hpp

stan::gm::set\_param\_ranges\_visgen::operator()

void operator()(const corr\_matrix\_var\_decl &x) const

**Definition:** generator.hpp:2443

stan::gm::statement\_visgen::operator()

void operator()(const no\_op\_statement &) const

**Definition:** generator.hpp:1742

stan::gm::validate\_transformed\_params\_visgen::operator()

void operator()(vector\_var\_decl const &x) const

**Definition:** generator.hpp:1397

stan::gm::visgen::visgen

visgen(std::ostream &o)

**Definition:** generator.hpp:67

stan::gm::write\_array\_vars\_visgen::operator()

void operator()(const corr\_matrix\_var\_decl &x) const

**Definition:** generator.hpp:4076

stan::gm::local\_var\_decl\_visgen::local\_var\_decl\_visgen

local\_var\_decl\_visgen(int indents, bool is\_var, bool is\_fun\_return, std::ostream &o)

**Definition:** generator.hpp:1000

stan::gm::write\_csv\_visgen::operator()

void operator()(const cholesky\_corr\_var\_decl &x) const

**Definition:** generator.hpp:3607

stan::gm::write\_csv\_vars\_visgen::operator()

void operator()(const vector\_var\_decl &x) const

**Definition:** generator.hpp:3704

stan::gm::generate\_comment

void generate\_comment(std::string const &msg, int indent, std::ostream &o)

**Definition:** generator.hpp:79

stan::gm::write\_array\_visgen::operator()

void operator()(const cholesky\_factor\_var\_decl &x) const

**Definition:** generator.hpp:3929

stan::gm::base\_expr\_type

int base\_expr\_type

**Definition:** ast.hpp:60

stan::gm::validate\_var\_decl\_visgen::generate\_end\_for\_dims

void generate\_end\_for\_dims(size\_t dims\_size) const

**Definition:** generator.hpp:802

stan::gm::generate\_local\_var\_init\_nan\_visgen::operator()

void operator()(const ordered\_var\_decl &x) const

**Definition:** generator.hpp:1267

stan::gm::is\_nil

bool is\_nil(const expression &e)

**Definition:** ast\_def.cpp:768

stan::gm::statement::statement\_

statement\_t statement\_

**Definition:** ast.hpp:650

stan::gm::init\_local\_var\_visgen::operator()

void operator()(const nil &) const

**Definition:** generator.hpp:577

stan::gm::init\_local\_var\_visgen::operator()

void operator()(const unit\_vector\_var\_decl &x) const

**Definition:** generator.hpp:601

stan::math::e

double e()

Return the base of the natural logarithm.

**Definition:** constants.hpp:86

stan::gm::generate\_local\_var\_init\_nan\_visgen::operator()

void operator()(const int\_var\_decl &x) const

**Definition:** generator.hpp:1246

stan::gm::generate\_init\_vars\_visgen::indent\_

int indent\_

**Definition:** generator.hpp:1308

stan::gm::var\_size\_validating\_visgen::operator()

void operator()(cholesky\_factor\_var\_decl const &x) const

**Definition:** generator.hpp:443

stan::gm::var\_size\_validating\_visgen::operator()

void operator()(cholesky\_corr\_var\_decl const &x) const

**Definition:** generator.hpp:446

stan::gm::var\_decl::decl\_

var\_decl\_t decl\_

**Definition:** ast.hpp:609

stan::gm::local\_var\_decl\_visgen::declare\_array

void declare\_array(const std::string &type, const std::vector< expression > &ctor\_args, const std::string &name, const std::vector< expression > &dims) const

**Definition:** generator.hpp:1189

stan::gm::constrained\_param\_names\_visgen::operator()

void operator()(const nil &) const

**Definition:** generator.hpp:3200

stan::gm::fun

**Definition:** ast.hpp:350

stan::gm::statement\_visgen::is\_fun\_return\_

bool is\_fun\_return\_

**Definition:** generator.hpp:1522

stan::gm::statement\_visgen::operator()

void operator()(const return\_statement &rs) const

**Definition:** generator.hpp:1685

stan::gm::printable\_visgen::printable\_visgen

printable\_visgen(std::ostream &o)

**Definition:** generator.hpp:273

stan::gm::dump\_member\_var\_visgen::operator()

void operator()(row\_vector\_var\_decl const &x) const

**Definition:** generator.hpp:1933

stan::gm::for\_statement::variable\_

std::string variable\_

**Definition:** ast.hpp:719

stan::gm::local\_var\_decl\_visgen::operator()

void operator()(int\_var\_decl const &x) const

**Definition:** generator.hpp:1010

stan::gm::constrained\_param\_names\_visgen::operator()

void operator()(const simplex\_var\_decl &x) const

**Definition:** generator.hpp:3228

stan::gm::printable\_visgen

**Definition:** generator.hpp:272

stan::gm::generate\_write\_csv\_method

void generate\_write\_csv\_method(const program &prog, const std::string &model\_name, std::ostream &o)

**Definition:** generator.hpp:3771

stan::gm::program::function\_decl\_defs\_

std::vector< function\_decl\_def > function\_decl\_defs\_

**Definition:** ast.hpp:796

stan::gm::validate\_var\_decl\_visgen::operator()

void operator()(cholesky\_corr\_var\_decl const &x) const

**Definition:** generator.hpp:895

stan::gm::sample::truncation\_

range truncation\_

**Definition:** ast.hpp:821

stan::gm::reject\_statement::printables\_

std::vector< printable > printables\_

**Definition:** ast.hpp:752

stan::math::size

int size(const std::vector< T > &x)

**Definition:** size.hpp:11

stan::gm::set\_param\_ranges\_visgen::operator()

void operator()(const nil &) const

**Definition:** generator.hpp:2332

stan::gm::expr\_type::is\_ill\_formed

bool is\_ill\_formed() const

**Definition:** ast\_def.cpp:105

stan::gm::generate\_end\_class\_decl

void generate\_end\_class\_decl(std::ostream &o)

**Definition:** generator.hpp:344

stan::gm::unit\_vector\_var\_decl

**Definition:** ast.hpp:472

stan::gm::function\_decl\_def::return\_type\_

expr\_type return\_type\_

**Definition:** ast.hpp:782

stan::gm::var\_size\_validating\_visgen::operator()

void operator()(int\_var\_decl const &x) const

**Definition:** generator.hpp:416

stan::gm::generate\_init\_visgen::operator()

void operator()(cov\_matrix\_var\_decl const &x) const

**Definition:** generator.hpp:2673

stan::gm::binary\_op

**Definition:** ast.hpp:382

stan::gm::range::has\_high

bool has\_high() const

**Definition:** ast\_def.cpp:940

stan::gm::constrained\_param\_names\_visgen::operator()

void operator()(const double\_var\_decl &x) const

**Definition:** generator.hpp:3204

stan::gm::generate\_initializer

void generate\_initializer(std::ostream &o, const std::string &base\_type, const std::vector< expression > &dims, const expression &type\_arg1=expression(), const expression &type\_arg2=expression())

**Definition:** generator.hpp:348

stan::gm::validate\_var\_decl\_visgen::indents\_

int indents\_

**Definition:** generator.hpp:784

stan::gm::init\_local\_var\_visgen::is\_var\_

const bool is\_var\_

**Definition:** generator.hpp:548

stan::gm::local\_var\_decl\_visgen::generate\_void\_statement

void generate\_void\_statement(const std::string &name) const

**Definition:** generator.hpp:1135

stan::gm::write\_array\_vars\_visgen::operator()

void operator()(const simplex\_var\_decl &x) const

**Definition:** generator.hpp:4043

stan::gm::set\_param\_ranges\_visgen::generate\_increment

void generate\_increment(expression M, expression N, std::vector< expression > dims) const

**Definition:** generator.hpp:2491

stan::gm::generate\_usings

void generate\_usings(std::ostream &o)

**Definition:** generator.hpp:297

stan::gm::generate\_local\_var\_init\_nan\_visgen::operator()

void operator()(const nil &) const

**Definition:** generator.hpp:1243

stan::gm::INT\_T

const int INT\_T

**Definition:** ast.hpp:65

stan::gm::statement\_visgen::operator()

void operator()(assignment const &x) const

**Definition:** generator.hpp:1536

stan::gm::local\_var\_decl\_visgen::operator()

void operator()(ordered\_var\_decl const &x) const

**Definition:** generator.hpp:1066

stan::gm::assignment::var\_type\_

base\_var\_decl var\_type\_

**Definition:** ast.hpp:831

stan::gm::write\_csv\_vars\_visgen::operator()

void operator()(const cholesky\_factor\_var\_decl &x) const

**Definition:** generator.hpp:3725

stan::gm::member\_var\_decl\_visgen::operator()

void operator()(matrix\_var\_decl const &x) const

**Definition:** generator.hpp:966

stan::gm::unconstrained\_param\_names\_visgen::operator()

void operator()(const cov\_matrix\_var\_decl &x) const

**Definition:** generator.hpp:3426

stan::gm::generate\_init\_vars\_visgen

**Definition:** generator.hpp:1307

stan::gm::statement\_visgen::operator()

void operator()(const conditional\_statement &x) const

**Definition:** generator.hpp:1718

stan::gm::validate\_var\_decl\_visgen::generate\_loop\_var

void generate\_loop\_var(const std::string &name, size\_t dims\_size) const

**Definition:** generator.hpp:809

stan::gm::statement\_visgen::operator()

void operator()(nil const &) const

**Definition:** generator.hpp:1534

stan::gm::write\_csv\_vars\_visgen::operator()

void operator()(const row\_vector\_var\_decl &x) const

**Definition:** generator.hpp:3707

stan::gm::return\_statement

**Definition:** ast.hpp:757

stan::gm::unconstrained\_param\_names\_visgen::operator()

void operator()(const ordered\_var\_decl &x) const

**Definition:** generator.hpp:3384

stan::gm::generate\_init\_visgen::generate\_init\_visgen

generate\_init\_visgen(std::ostream &o)

**Definition:** generator.hpp:2562

stan::gm::member\_var\_decl\_visgen::operator()

void operator()(const cholesky\_corr\_var\_decl &x) const

**Definition:** generator.hpp:951

stan::gm::fun\_scalar\_type

std::string fun\_scalar\_type(const function\_decl\_def &fun, bool is\_lp)

**Definition:** generator.hpp:4309

stan::gm::statement\_visgen::is\_var\_

bool is\_var\_

**Definition:** generator.hpp:1521

stan::gm::write\_array\_visgen::operator()

void operator()(const cholesky\_corr\_var\_decl &x) const

**Definition:** generator.hpp:3935

stan::gm::generate\_public\_decl

void generate\_public\_decl(std::ostream &o)

**Definition:** generator.hpp:774

stan::gm::validate\_var\_decl\_visgen::operator()

void operator()(matrix\_var\_decl const &x) const

**Definition:** generator.hpp:865

stan::gm::generate\_init\_visgen::operator()

void operator()(nil const &) const

**Definition:** generator.hpp:2566

stan::gm::constrained\_param\_names\_visgen::operator()

void operator()(const corr\_matrix\_var\_decl &x) const

**Definition:** generator.hpp:3261

is\_matrix

bool is\_matrix(const std::string &parameter\_name)

**Definition:** print.hpp:116

stan::gm::write\_csv\_visgen::operator()

void operator()(const unit\_vector\_var\_decl &x) const

**Definition:** generator.hpp:3581

stan::gm::constrained\_param\_names\_visgen::operator()

void operator()(const cholesky\_factor\_var\_decl &x) const

**Definition:** generator.hpp:3243

stan::gm::validate\_var\_decl\_visgen::operator()

void operator()(cov\_matrix\_var\_decl const &x) const

**Definition:** generator.hpp:898

stan::gm::generate\_init\_visgen::operator()

void operator()(double\_var\_decl const &x) const

**Definition:** generator.hpp:2599

stan::gm::distribution::args\_

std::vector< expression > args\_

**Definition:** ast.hpp:196

stan::gm::member\_var\_decl\_visgen::operator()

void operator()(double\_var\_decl const &x) const

**Definition:** generator.hpp:933

stan::gm::unconstrained\_param\_names\_visgen::operator()

void operator()(const vector\_var\_decl &x) const

**Definition:** generator.hpp:3358

stan::gm::write\_dims\_visgen::operator()

void operator()(const ordered\_var\_decl &x) const

**Definition:** generator.hpp:2864

stan::MAJOR\_VERSION

const std::string MAJOR\_VERSION

Major version number for Stan package.

**Definition:** version.hpp:9

stan::gm::has\_lb

bool has\_lb(const D &x)

**Definition:** generator.hpp:39

stan::gm::write\_array\_visgen::operator()

void operator()(const row\_vector\_var\_decl &x) const

**Definition:** generator.hpp:3898

stan::gm::sample::dist\_

distribution dist\_

**Definition:** ast.hpp:820

stan::gm::set\_param\_ranges\_visgen::operator()

void operator()(const cholesky\_factor\_var\_decl &x) const

**Definition:** generator.hpp:2396

stan::gm::write\_param\_names\_visgen

**Definition:** generator.hpp:2948

stan::gm::vector\_var\_decl

**Definition:** ast.hpp:504

stan::gm::constrained\_param\_names\_visgen::constrained\_param\_names\_visgen

constrained\_param\_names\_visgen(std::ostream &o)

**Definition:** generator.hpp:3197

stan::gm::generate\_init\_vars

void generate\_init\_vars(const std::vector< var\_decl > &vs, int indent, std::ostream &o)

**Definition:** generator.hpp:1369

stan::gm::write\_param\_names\_visgen::operator()

void operator()(const matrix\_var\_decl &x) const

**Definition:** generator.hpp:2965

stan::gm::write\_array\_vars\_visgen::operator()

void operator()(const row\_vector\_var\_decl &x) const

**Definition:** generator.hpp:4027

stan::gm::write\_param\_names\_visgen::operator()

void operator()(const corr\_matrix\_var\_decl &x) const

**Definition:** generator.hpp:2989

stan::gm::function\_decl\_def::body\_

statement body\_

**Definition:** ast.hpp:785

stan::gm::write\_array\_visgen::operator()

void operator()(const matrix\_var\_decl &x) const

**Definition:** generator.hpp:3903

stan::gm::validate\_var\_decl\_visgen::nonbasic\_validate

void nonbasic\_validate(const T &x, const std::string &type\_name) const

**Definition:** generator.hpp:869

stan::gm::write\_csv\_vars\_visgen::operator()

void operator()(const double\_var\_decl &x) const

**Definition:** generator.hpp:3701

stan::gm::write\_array\_visgen::operator()

void operator()(const ordered\_var\_decl &x) const

**Definition:** generator.hpp:3919

stan::gm::write\_csv\_visgen::operator()

void operator()(const cov\_matrix\_var\_decl &x) const

**Definition:** generator.hpp:3612

stan::gm::write\_param\_names\_visgen::operator()

void operator()(const simplex\_var\_decl &x) const

**Definition:** generator.hpp:2971

stan::gm::EOL

const std::string EOL("\n")

stan::gm::while\_statement

**Definition:** ast.hpp:737

stan::gm::generate\_validate\_positive

void generate\_validate\_positive(const std::string &var\_name, const expression &expr, std::ostream &o)

**Definition:** generator.hpp:458

stan::gm::write\_array\_vars\_visgen::operator()

void operator()(const double\_var\_decl &x) const

**Definition:** generator.hpp:4019

stan::gm::write\_dims\_visgen

**Definition:** generator.hpp:2827

stan::gm::constrained\_param\_names\_visgen::operator()

void operator()(const matrix\_var\_decl &x) const

**Definition:** generator.hpp:3217

stan::gm::set\_param\_ranges\_visgen::operator()

void operator()(const positive\_ordered\_var\_decl &x) const

**Definition:** generator.hpp:2393

stan::gm::write\_array\_vars\_visgen::operator()

void operator()(const unit\_vector\_var\_decl &x) const

**Definition:** generator.hpp:4038

stan::gm::write\_csv\_visgen::operator()

void operator()(const matrix\_var\_decl &x) const

**Definition:** generator.hpp:3575

stan::gm::member\_var\_decl\_visgen::member\_var\_decl\_visgen

member\_var\_decl\_visgen(int indents, std::ostream &o)

**Definition:** generator.hpp:924

stan::gm::init\_local\_var\_visgen::operator()

void operator()(const ordered\_var\_decl &x) const

**Definition:** generator.hpp:611

stan::gm::write\_array\_visgen::operator()

void operator()(const nil &) const

**Definition:** generator.hpp:3861

stan::gm::constrained\_param\_names\_visgen::operator()

void operator()(const ordered\_var\_decl &x) const

**Definition:** generator.hpp:3233

stan::gm::unconstrained\_param\_names\_visgen::operator()

void operator()(const cholesky\_corr\_var\_decl &x) const

**Definition:** generator.hpp:3413

stan::gm::statements::local\_decl\_

std::vector< var\_decl > local\_decl\_

**Definition:** ast.hpp:186

version.hpp

stan::gm::print\_statement::printables\_

std::vector< printable > printables\_

**Definition:** ast.hpp:746

stan::gm::expression::expression\_type

expr\_type expression\_type() const

**Definition:** ast\_def.cpp:570

stan::gm::validate\_transformed\_params\_visgen::operator()

void operator()(positive\_ordered\_var\_decl const &x) const

**Definition:** generator.hpp:1417

stan::gm::write\_csv\_header\_visgen::operator()

void operator()(const int\_var\_decl &x) const

**Definition:** generator.hpp:3036

stan::gm::printable\_visgen::operator()

void operator()(const expression &e) const

**Definition:** generator.hpp:277

stan::gm::write\_dims\_visgen::operator()

void operator()(const corr\_matrix\_var\_decl &x) const

**Definition:** generator.hpp:2892

stan::gm::generate\_local\_var\_init\_nan\_visgen

**Definition:** generator.hpp:1227

stan::gm::set\_param\_ranges\_visgen::operator()

void operator()(const cholesky\_corr\_var\_decl &x) const

**Definition:** generator.hpp:2415

stan::gm::write\_csv\_header\_visgen::operator()

void operator()(const vector\_var\_decl &x) const

**Definition:** generator.hpp:3042

stan::gm::matrix\_var\_decl::M\_

expression M\_

**Definition:** ast.hpp:526

stan::gm::generate\_local\_var\_init\_nan\_visgen::operator()

void operator()(const vector\_var\_decl &x) const

**Definition:** generator.hpp:1252

stan::gm::generate\_init\_vars\_visgen::operator()

void operator()(double\_var\_decl const &x) const

**Definition:** generator.hpp:1319

stan::gm::statement\_visgen::operator()

void operator()(const print\_statement &ps) const

**Definition:** generator.hpp:1659

stan::gm::generate\_init\_visgen::operator()

void operator()(ordered\_var\_decl const &x) const

**Definition:** generator.hpp:2645

stan::gm::INDENT2

const std::string INDENT2(" ")

stan::gm::has\_lub

bool has\_lub(const D &x)

**Definition:** generator.hpp:31

stan::gm::arg\_decl::arg\_type\_

expr\_type arg\_type\_

**Definition:** ast.hpp:768

stan::gm::statement\_visgen::operator()

void operator()(const for\_statement &x) const

**Definition:** generator.hpp:1696

stan::gm::generate\_init\_visgen::operator()

void operator()(unit\_vector\_var\_decl const &x) const

**Definition:** generator.hpp:2631

stan::gm::generate\_init\_method

void generate\_init\_method(const std::vector< var\_decl > &vs, std::ostream &o)

**Definition:** generator.hpp:2795

stan::gm::statements::statements\_

std::vector< statement > statements\_

**Definition:** ast.hpp:187

stan::gm::member\_var\_decl\_visgen::indents\_

int indents\_

**Definition:** generator.hpp:923

stan::gm::generate\_local\_var\_init\_nan\_visgen::is\_var\_

const bool is\_var\_

**Definition:** generator.hpp:1229

stan::gm::statement\_visgen::operator()

void operator()(const statements &x) const

**Definition:** generator.hpp:1638

stan::gm::generate\_local\_var\_init\_nan\_visgen::is\_fun\_return\_

const bool is\_fun\_return\_

**Definition:** generator.hpp:1230

stan::gm::validate\_transformed\_params\_visgen::operator()

void operator()(int\_var\_decl const &x) const

**Definition:** generator.hpp:1389

stan::gm::write\_csv\_visgen::operator()

void operator()(const double\_var\_decl &x) const

**Definition:** generator.hpp:3561

stan::gm::generate\_private\_decl

void generate\_private\_decl(std::ostream &o)

**Definition:** generator.hpp:778

stan::gm::positive\_ordered\_var\_decl::K\_

expression K\_

**Definition:** ast.hpp:497

stan::gm::generate\_write\_csv\_header\_method

void generate\_write\_csv\_header\_method(const program &prog, std::ostream &o)

**Definition:** generator.hpp:3173

stan::gm::write\_array\_vars\_visgen::operator()

void operator()(const ordered\_var\_decl &x) const

**Definition:** generator.hpp:4048

stan::gm::generate\_write\_array\_method

void generate\_write\_array\_method(const program &prog, const std::string &model\_name, std::ostream &o)

**Definition:** generator.hpp:4131

stan::gm::local\_var\_decl\_visgen::operator()

void operator()(cholesky\_factor\_var\_decl const &x) const

**Definition:** generator.hpp:1082

stan::gm::dump\_member\_var\_visgen::operator()

void operator()(nil const &) const

**Definition:** generator.hpp:1840

stan::gm::expr\_type::is\_void

bool is\_void() const

**Definition:** ast\_def.cpp:108

stan::gm::VOID\_T

const int VOID\_T

**Definition:** ast.hpp:64

stan::gm::write\_param\_names\_visgen::generate\_param\_names

void generate\_param\_names(const std::string &name) const

**Definition:** generator.hpp:2993

stan::gm::validate\_transformed\_params\_visgen::operator()

void operator()(corr\_matrix\_var\_decl const &x) const

**Definition:** generator.hpp:1451

stan::gm::write\_param\_names\_visgen::operator()

void operator()(const vector\_var\_decl &x) const

**Definition:** generator.hpp:2959

stan::gm::validate\_var\_decl\_visgen

**Definition:** generator.hpp:783

stan::gm::arg\_decl

**Definition:** ast.hpp:767

stan::gm::write\_param\_names\_visgen::operator()

void operator()(const nil &) const

**Definition:** generator.hpp:2952


---

[ Stan Home Page ]

*© 2011–2014,
Stan Development Team.*
